# Evolutionarily Diverged Regulation of X-chromosomal Genes as a Primal Event in Mouse Reproductive Isolation

**DOI:** 10.1371/journal.pgen.1004301

**Published:** 2014-04-17

**Authors:** Ayako Oka, Toyoyuki Takada, Hironori Fujisawa, Toshihiko Shiroishi

**Affiliations:** 1 Transdisciplinary Research Integration Center, Research Organization of Information and Systems, Toranomon, Tokyo, Japan; 2 Mammalian Genetics Laboratory, National Institute of Genetics, Mishima, Shizuoka, Japan; 3 The Institute of Statistical Mathematics, Tachikawa, Tokyo, Japan; University of North Carolina at Chapel Hill, United States of America

## Abstract

Improper gene regulation is implicated in reproductive isolation, but its genetic and molecular bases are unknown. We previously reported that a mouse inter-subspecific X chromosome substitution strain shows reproductive isolation characterized by male-specific sterility due to disruption of meiotic entry in spermatogenesis. Here, we conducted comprehensive transcriptional profiling of the testicular cells of this strain by microarray. The results clearly revealed gross misregulation of gene expression in the substituted donor X chromosome. Such misregulation occurred prior to detectable spermatogenetic impairment, suggesting that it is a primal event in reproductive isolation. The misregulation of X-linked genes showed asymmetry; more genes were disproportionally downregulated rather than upregulated. Furthermore, this misregulation subsequently resulted in perturbation of global transcriptional regulation of autosomal genes, probably by cascading deleterious effects. Remarkably, this transcriptional misregulation was substantially restored by introduction of chromosome 1 from the same donor strain as the X chromosome. This finding implies that one of regulatory genes acting in *trans* for X-linked target genes is located on chromosome 1. This study collectively suggests that regulatory incompatibility is a major cause of reproductive isolation in the X chromosome substitution strain.

## Author Summary

Reproductive isolation characterized by male sterility and decreased viability is important for speciation, because it suppresses free genetic exchange between two diverged populations and accelerates the genetic divergence. One of the reproductive isolation phenomena, hybrid sterility (sterility in hybrid animals), is possibly caused by deleterious interactions between diverged genetic factors brought by two distinct populations. The polymorphism not only in protein-coding sequences but also in transcriptional regulatory sequences can cause the genetic incompatibility in hybrid animals. However, the precise genetic mechanisms of hybrid sterility are mostly unknown. Here, we report that the expression of X-linked genes derived from one mouse subspecies was largely misregulated in the genetic background of another subspecies. The misregulated expression of the X-linked genes subsequently affected the global expression of autosomal genes. The results collectively indicate that hybrid sterility between the two mouse subspecies is caused by misregulation of gene expression due to genetic incompatibility in the transcriptional regulatory circuitry. Such genetic incompatibility in transcriptional regulation likely underlies reproductive isolation in general.

## Introduction

Reproductive isolation is a typical consequence of deleterious epistatic interactions between genes that have evolutionarily diverged in species or subspecies [1]–[3]. One of the most common types of postzygotic reproductive isolation is sterility of interspecific (or intersubspecific) hybrid progeny in F1 or later intercross or backcross generations. Although numerous sterility factors are mapped genetically, only a limited number of responsible genes have been cloned in mammals and non-mammalian vertebrates [4]. A confounding factor that makes it difficult to identify sterility-causing genes is that these genes function properly in their parental pure species (or subspecies), and deleterious interactions (i.e., genetic incompatibility) between them only occur in the hybrid genetic background [4]. Genetic incompatibility occurs in various levels, not only in physical interactions between responsible gene products (e.g., proteins), but also in the balance between expression levels of the responsible genes. Using hybrid animals between two mouse subspecies, Goncalves *et al.* [5] reported that the expression of approximately 30% of non-imprinted autosomal genes expressed in the liver is diverged by variants in *cis*-regulatory elements and variants in regulatory factors acting in *trans*. Furthermore, the rates of variants in *cis*-regulatory elements is higher than those in regulatory factors acting in *trans*. Such evolutionary divergence in transcriptional regulation may contribute to phenotypic differences and genetic incompatibilities underlying reproductive isolation.

Experimental genetic studies of animals including mice (*Mus musculus*) and fruit fly (*Drosophila*) suggest that the X chromosome often plays a central role in the sterility of hybrid males between species (or subspecies). The genetic loci that cause hybrid disruptions appear to be preferentially located on the X chromosome, which is referred to as the “large X-effect” [6]. The most convincing hypothesis to explain the large X-effect is that male reproductive genes preferentially accumulate on the X chromosome because of the immediate exposure of beneficial recessive mutations due to hemizygosity in males, and such X-linked reproductive genes evolve rapidly by positive selection [7], [8]. On the other hand, transcriptional inactivation during spermatogenic meiosis (i.e., meiotic sex chromosome inactivation [MSCI]) and postmeiotic stages (i.e., postmeiotic sex chromosome repression [PMSR]) influence the genetic content of the X chromosome. Thus, spermatogenic genes expressed during the premeiotic stage are preferentially enriched on the mouse X chromosome [9], [10] and the human X chromosome [11]. Despite the accumulation of premeiotically expressing genes on the X chromosome, many hybrid male mice show spermatogenic defects during meiotic and postmeiotic stages such as spermiogenesis [12]–[15]. Thus far, such discrepancy has not been fully understood.

Two mouse subspecies, *M. m domesticus* and *M. m. musculus*, diverged about 0.5 million years ago [16], and they manifest the early-stage of hybrid incompatibility phenotypes. Laboratory crosses between *M. m. domesticus* and *M. m. musculus* often yield fertile females, but sterile males [17]. The first mammalian hybrid sterility gene, PR domain containing 9 (*Prdm9*), was identified as the gene responsible for F1 hybrid sterility between *M. m. musculus* and *M. m. domesticus* [18]. Incompatibility between two *Prdm9* alleles alone is not sufficient to drive reproductive isolation. Instead, the gene dosage of *Prdm9* and combinations of particular *Prdm9* alleles in conjunction with functional incompatibility with other X-linked gene(s) are important factors [19].

Natural habitats of *M. m. musculus* and *M. m. domesticus* overlap in Europe forming a hybrid zone, where hybrid populations exhibit reduced fertility and barriers to gene flow. It is known that X chromosomal genes have more limited flow beyond the hybrid zone than autosomal ones, suggesting a major role for the X chromosome in the reproductive isolation between the two subspecies [20]–[24]. The prominent role of the X chromosome was also supported by laboratory studies using F2 male progeny between the strains derived from the two subspecies [25] and the chromosome substitution strains, in which the X chromosome of the host strains (C57BL/6J [B6], predominantly derived from *M. m. domesticus* or wild *M. m. domesticus*-derived LEWES/EiJ) is substituted by the counterparts of the wild *M. m. musculus*-derived strains [15], [26]. We also reported a similar male-specific reproductive phenotype characterized by complete sterility in another chromosome substitution strain, B6-ChrX^MSM^, in which the entire length of the X chromosome is substituted by the counterpart of the *M. m. molossinus*-derived MSM strain in the genetic background of the B6 strain [13], [27], [28]. *M. m. molossinus* is an evolutional hybrid between *M. m. musculus* and *M. m. castaneus*, and its nuclear genome is predominantly derived from *M. m. musculus* [29]–[32]. Therefore, these observations imply that the same or a similar genetic basis underlies the male sterility of the X chromosome substitution strains that carry *M. m. musculus*-derived or *M. m. molossinus*-derived X chromosome in the genetic background of *M. m. domesticus*.

The most prominent and first detectable phenotype in B6-ChrX^MSM^ males is a small number of primary spermatocytes during the first cycle of spermatogenesis, suggesting a defect in meiotic entry [13]. This phenotype ultimately results in a decreased number of meiotic and post-meiotic germ cells, and in turn contributes to a reduction of testis weight. Our previous quantitative trait locus (QTL) analysis mapped the responsible gene(s) for the reduced testis weight of adult males to the distal region of the X chromosome [27].

In this study, we report a sub-fertile phenotype in a newly established partial chromosome substitution strain, B6-ChrXT^MSM^, which has the MSM-derived distal half of the X chromosome in the genetic background of the B6 strain. To detect the primary event at the premeiotic stage, we conducted genome-wide expression profiling by microarray analyses of the testes of prepubertal B6-ChrX^MSM^ and B6-ChrXT^MSM^ males. Compared with the B6 strain, we found differential expression for 20% of MSM-derived X-linked genes that mostly show downregulation. Furthermore, the altered expression of X-linked genes subsequently evokes perturbation of genome-wide transcriptional regulation of autosomal genes. Notably, the differential expression in B6-ChrXT^MSM^ is substantially restored in F1 progeny generated by crossing B6-ChrXT^MSM^ females and B6-Chr1^MSM^ males, in which chromosome 1 is substituted by the counterpart of the MSM strain in the genetic background of the B6 strain. This observation suggests that chromosome 1 contains upstream regulatory genes for X-linked target genes and the genetic incompatibility between *trans*-acting regulatory genes on chromosome 1 and *cis*-regulatory elements of the X-linked target genes is involved in the differential gene expression in B6-ChrX^MSM^ and B6-ChrXT^MSM^ testes. Intriguingly, we found that the differential expression also occurs in genes on chromosome 1 of F1 male progeny, implying that a similar phenomenon may generally occur in any donor chromosome of the inter-subspecific chromosome substitution strains. Our data suggest that the transcriptional regulatory system has diverged at the whole genome level during mouse evolution. Furthermore, the incompatibility of the diverged gene regulation between the two mouse subspecies results in reproductive isolation of X chromosome substitution strains.

## Results

### Differential Expression of X-linked Genes in B6-ChrX^MSM^ Testes

In mice, spermatogenesis begins a few days after birth. Spermatogonia, which represent the mitotic stages of spermatogenic cells, occupy the basal compartment of seminiferous tubules, where they proliferate and differentiate to give rise to spermatocytes. Primary spermatocytes subsequently go through two rounds of meiotic division to form four haploid round spermatids. Finally, round spermatids transform into sperm. Primary spermatocytes are first detected at around 10 days postpartum (dpp), and the first meiotic prophase continues for 10–12 days [33].

Histological observation of the testes of B6-ChrX^MSM^ and B6-ChrXT^MSM^ strains showed no detectable impairment at 8 dpp, and a perceptible change was initially apparent at 10 dpp ([13] and Figure S1A). At 14 dpp, we noted a clear defect. Primary spermatocytes were abundant in the seminiferous tubules of control animals, but rarely observed in B6-ChrX^MSM^ and B6-ChrXT^MSM^ tubules ([13] and Figure S1A). To estimate the frequency of primary spermatocytes at prophase of meiosis I, testicular cells from 18-day-old males were immunostained with an antibody against SYCP3, a component of the synaptonemal complex and marker of early spermatocytes. The frequency of SYCP3-positive spermatocytes among all testicular cells was significantly low in B6-ChrX^MSM^ and B6-ChrXT^MSM^ males compared with that in B6 males, suggesting an impairment of meiotic entry in the two strains (B6, N = 4, 46.9±7.8%; B6-ChrX^MSM^, N = 4, 6.4±2.7%; B6-ChrXT^MSM^, N = 4, 18.0±4.0%; Bonferroni-corrected *P*<0.01 by two-tailed Student's *t*-test; [13] and Figure S1B). A small number of meiotic spermatocytes likely cause low production of sperm and a subsequent reduction of the testis weight. Consistent with this notion, adult B6-ChrX^MSM^ and B6-ChrXT^MSM^ males showed a significant reduction of testis weight compared with that of B6 males (weight of paired testes: B6, N = 8, 224.3±6.9 mg; B6-ChrX^MSM^, N = 3, 144.0±8.2 mg; B6-ChrXT^MSM^, N = 8, 138.1±11.1 mg; Bonferroni-corrected *P*<0.01 by two-tailed Student's *t*-test; [27] and Figure S1C).

Our previous composite interval mapping and newly performed interval mapping showed that the QTL responsible for the reduced testis weight was located at the distal region of the X chromosome with a significantly high LOD score ([27] and Figure S1D). The highest peak was detected at 147 Mb by the interval mapping (LOD score = 27.5). The degree of reduction in testis weight of B6-ChrX^MSM^ males was similar to that of B6-ChrXT^MSM^ males. Moreover, these two strains carry MSM alleles at the QTL, which affect the testis weight. Thus, the same biological basis may underlie the reduced testis weight and small number of meiotic spermatocytes in the two strains.

To detect the primary event that occurs in testicular cells prior to meiotic entry, we conducted whole-genome transcriptional profiling by microarray analysis of total RNA from whole testes of 5- and 7-day-old males. At these days of age, testicular histology of the two strains was indistinguishable from that of B6 ([13] and Figure S1A). The cellular composition of the testes at 5 dpp is relatively simple. Spermatogonia and Sertoli cells comprise 8.0% and 88.5% of the seminiferous epithelium, respectively [33]. At 7 dpp, a proportion of undifferentiated spermatogonia undergoes proliferation and gives rise to differentiated spermatogonia, resulting in a gradual increase of the proportion of spermatogonia [34], [35]. For transcriptional profiling, we used an Affymetrix Mouse Genome 430 2.0 array. The donor (MSM) chromosomes in the chromosome substitution strains were derived from *M. m. molossinus* in which the genome is significantly diverged from the laboratory mouse genome [36]. For this reason, it is not appropriate to use assignment of the presence or absence of the hybridization signal by comparison of the perfect match (PM) and mismatch (MM) probes. Therefore, we calculated the gene expression level with each probe set using the robust multichip average (RMA), as implemented in GeneSpring GX software, which considers only PM probes in its estimation of the expression level with each probe set [37]. Furthermore, single nucleotide polymorphisms in PM probes possibly cause mishybridization, which may lead to undercounting the expression signals of MSM-derived alleles. To avoid this occurrence, significantly polymorphic probe sets were excluded from the analysis. Similarities of all PM probe sequences of the Mouse Genome 430 2.0 array were searched against the MSM sequence reads (DRA000194) by Megablast [36]. To evaluate polymorphisms in the probe sets, we performed scoring using a classifier based on the number of identified probes in the MSM genome and the number of perfectly matched probes with the MSM sequence. The polymorphism score and number of probe sets for each polymorphism score are shown in Figure S2. Probe sets with polymorphism scores of ≤10 were judged as “conserved” probe sets, and “polymorphic” probe sets (a polymorphism score of >10) were excluded from the analysis. Polymorphism score for all probe sets is available on the FTP site of the NIG Mouse Genome Database (ftp://anonymous@molossinus.lab.nig.ac.jp/pub/msmdb/Affy_Probe_Info_MSM.zip). The polymorphic probe sets excluded from the present analysis were listed in Table S1.

**Figure 1:**
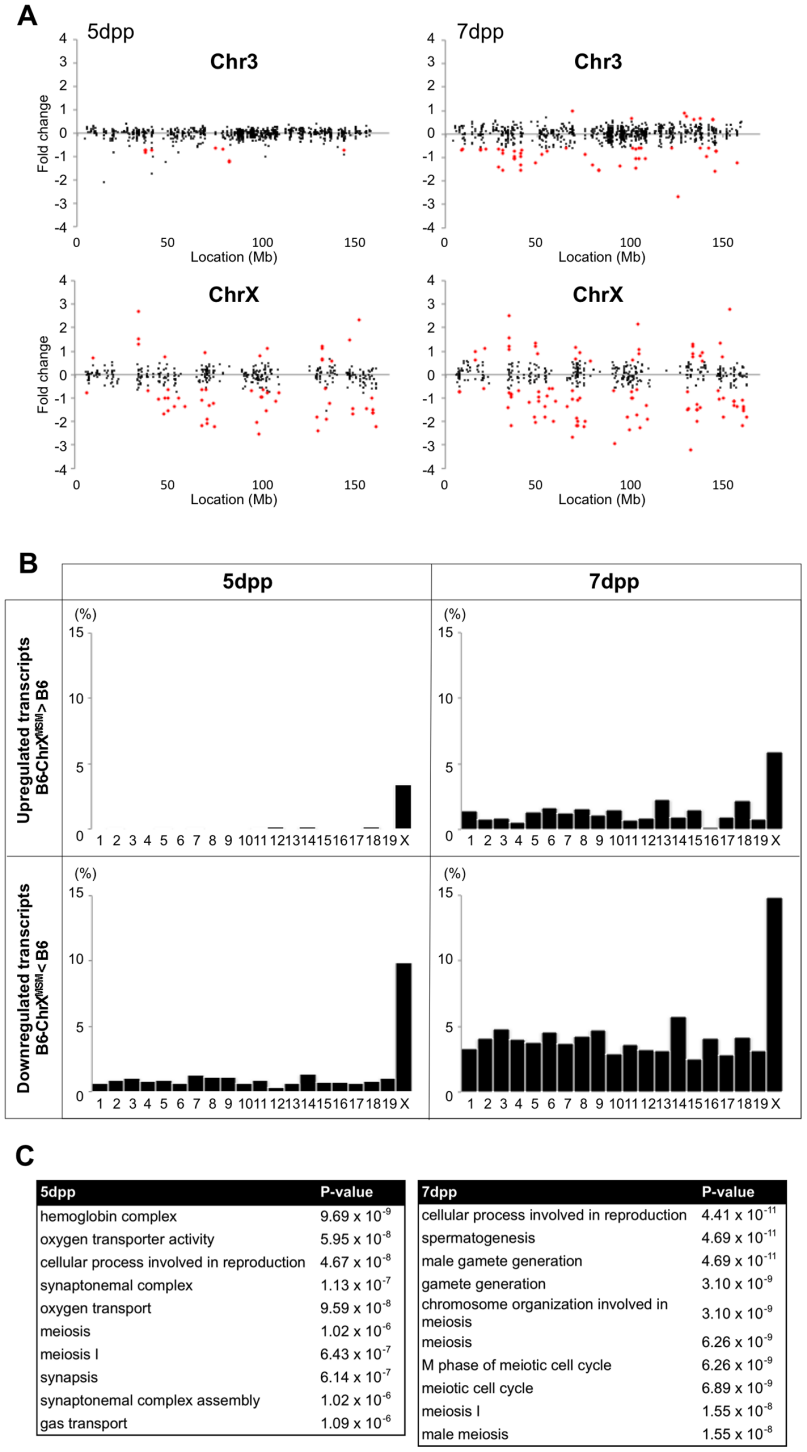
Differential expression of X-linked genes in B6-ChrX^MSM^ testes. (A) Gene expression on control chromosome 3 and the X chromosome in B6-ChrX^MSM^ testes. Each dot indicates the expression intensity of the transcript detected by a probe set on the microarray. The fold change (relative signal intensity of B6-ChrX^MSM^ to that of B6) is indicated by the log_2_ scale. Transcripts with a significantly different expression level are indicated in red (Benjamini-Hochberg FDR corrected moderate *t*-test, *P*<0.05; fold change ≥1.50). Left and right graphs show the results from 5 dpp and 7 dpp samples, respectively. Note that transcripts detected by X-linked probe sets showed a large variance of fold change compared with chromosome 3. The variance of fold change was larger at 7 dpp. (B) Frequency of upregulated and downregulated transcripts of each chromosome in B6-ChrX^MSM^ testes. The percentage of differentially expressed transcripts among all expressed transcripts is shown. Chromosomes are indicated below the graphs. A higher frequency of upregulated and downregulated transcripts was observed in the X chromosome than that in the autosomes. Downregulated genes were more common than upregulated genes. Differential expression relative to B6 was predominately genome-wide at 7 dpp. (C) GO categories and *P*-values of differentially expressed genes. Left and right tables show the results at 5 dpp and 7 dpp, respectively.

We performed the transcriptional profiling using microarray expression data of B6-ChrX^MSM^ testes at 5 dpp. Of the 1,376 probe sets for genes located in the genomic region from 5,341,800 to 163,344,914 bp on the X chromosome, 373 polymorphic probe sets were excluded from the analysis. Of the 43,678 probe sets for autosomal and non-polymorphic X chromosomal genes, 16,546 remained after filtering by the expression signal intensity (see Methods). To investigate the effect of the MSM-derived X chromosome on genome-wide gene expression, we plotted base-2 logarithms of the fold changes of expression levels for 16,546 transcripts in B6-ChrX^MSM^ testes relative to those in the B6 strain. Results of the transcriptional profiling of the X chromosome and control chromosome 3, which has a physical size similar to that of the X chromosome, are shown in Figure 1A. Results of other chromosomes are shown in Figure S3. We next applied filtering to volcano plots that are constructed using fold change values and statistical significance by the Benjamini-Hochberg FDR corrected moderated *t*-test (fold change ≥1.50; *P*<0.05). Transcripts with significantly differential expression detected by filtered probe sets are indicated by red dots in Figure 1A and S3. The frequency of such transcripts in each chromosome is shown in Figure 1B. Both upregulated and downregulated transcripts relative to the B6 strain were notably enriched on the X chromosome. In B6-ChrX^MSM^ testes, upregulated and downregulated transcripts from the X chromosome were detected by 3.4% (15/437) and 9.84% (43/437) of the probe sets, respectively. In contrast, upregulated and downregulated transcripts from autosomes were detected by 0.95% and 0.84% of the probe sets, respectively (Table 1 and Figure 1B). Interestingly, the direction of differential expression in B6-ChrX^MSM^ testes was asymmetric as indicated by a disproportionately higher number of downregulated transcripts. This asymmetry was also detected for transcripts from the autosomes, although it was less obvious than that for transcripts from the X chromosome.

**Table 1:** Frequency of downregulated and upregulated transcripts for each chromosome in B6-ChrX^MSM^ testes.

Chr	All probe sets	5 dpp			7 dpp		
		expressed probe sets	upregulated (%)	downregulated (%)	expressed probe sets	upregulated (%)	downregulated (%)
1	2839	1141	0 (0.00)	7 (0.61)	1215	17 (1.40)	40 (3.29)
2	3540	1345	1 (0.07)	12 (0.89)	1475	12 (0.81)	60 (4.07)
3	2292	869	0 (0.00)	9 (1.04)	927	8 (0.86)	45 (4.85)
4	2648	1029	0 (0.00)	8 (0.78)	1113	6 (0.54)	45 (4.04)
5	2769	1033	1 (0.10)	9 (0.87)	1125	15 (1.33)	43 (3.82)
6	2472	811	0 (0.00)	5 (0.62)	895	15 (1.68)	41 (4.58)
7	2941	1016	1 (0.10)	13 (1.28)	1103	14 (1.27)	41 (3.72)
8	2145	819	0 (0.00)	9 (1.10)	889	14 (1.57)	38 (4.27)
9	2349	886	0 (0.00)	10 (1.13)	971	11 (1.13)	46 (4.74)
10	2088	789	0 (0.00)	5 (0.63)	850	13 (1.53)	25 (2.94)
11	3410	1363	1 (0.07)	12 (0.88)	1447	10 (0.69)	52 (3.59)
12	1709	661	1 (0.15)	2 (0.30)	716	6 (0.84)	23 (3.21)
13	1804	659	0 (0.00)	4 (0.61)	704	16 (2.27)	22 (3.13)
14	1624	600	1 (0.17)	8 (1.33)	641	6 (0.94)	37 (5.77)
15	1782	668	0 (0.00)	5 (0.75)	721	11 (1.53)	18 (2.50)
16	1543	585	0 (0.00)	4 (0.68)	634	1 (0.16)	26 (4.10)
17	1919	750	0 (0.00)	5 (0.67)	811	8 (0.99)	23 (2.84)
18	1287	513	1 (0.19)	4 (0.78)	547	12 (2.19)	23 (4.20)
19	1514	572	0 (0.00)	6 (1.05)	634	5 (0.79)	20 (3.15)
X	1003	437	15 (3.43)	43 (9.84)	472	28 (5.93)	70 (14.83)

We examined whether fold change variance was different between X chromosomal and autosomal transcripts by the Ansari-Bradley test for centralized data, which is a commonly used rank-based test [38]. The results showed that the fold change variance of the X chromosomal transcripts was significantly larger than that of the autosomes (Ansari-Bradley test, *P*≤1.00×10^−16^). To test whether the different variances of the fold changes were independent of the expression level of the transcripts, we classified the probe sets into three groups by the raw signal intensity. The Ansari-Bradley test showed that the variance of the fold change for the X chromosome and autosomes was significantly different for both the low expression group (raw signal intensity between 10 and 100) and the intermediate expression group (raw signal intensity between 100 and 1000) (Table S2). We could not test for the high expression group (raw signal intensity of >1000), because such signal intensities were not observed for the X chromosome. For autosomes, we found 208 transcripts in the high expression group, among which more than half (125/208 transcripts) encode nuclear or mitochondrial ribosomal proteins. Because the haploinsufficiency of ribosomal protein genes immediately affects translational activity in many mutant mice, only a small number of ribosomal genes are located on the X chromosome, which is protected from X-inactivation in female somatic cells and male germ cells, such as MSCI and PMSR [39], [40].

We next performed transcriptional profiling of B6-ChrX^MSM^ testes at 7 dpp. Of the 43,678 autosomal and non-polymorphic X chromosomal probe sets, 17,890 remained after filtering by the expression signal intensity. Similar to 5 dpp, differential expression of X chromosomal genes relative to the B6 strain was observed in B6-ChrX^MSM^ testes at 7 dpp. Upregulated and downregulated transcripts were detected by 5.93% (28/472) and 14.83% (70/472) of the probe sets, respectively (Figure 1A, 1B and S4 and Table 1). In contrast, upregulated and downregulated transcripts from autosomes were detected by 1.19% and 3.83% of the probe sets, respectively. Differential expression detected by the X chromosomal probe sets was more significant than that detected by the autosomal probe sets, which was irrespective of the expression levels (Ansari-Bradley test, *P*≤1.00×10^−16^; Table S2). The differential expression of both X chromosomal and autosomal transcripts became more obvious at 7 dpp compared with that at 5 dpp (Ansari-Bradley test, autosomes, *P*≤1.00×10^−16^; X chromosome, *P* = 2.98×10^−5^). Differentially expressed transcripts in 5- and 7-day-old B6-ChrX^MSM^ testes are listed in Tables S3 and S4. These data indicate that transcripts with highly differential expression are preferentially located on the X chromosome. Gene ontology (GO) analysis revealed that differentially expressed transcripts on autosomes and the X chromosome were drastically biased toward those involved in meiotic processes such as synapsis, synaptonemal complex, M-phase of the meiotic cell cycle, and meiotic chromosome organization as well as general male gamete production (Figure 1C).

**Figure 2:**
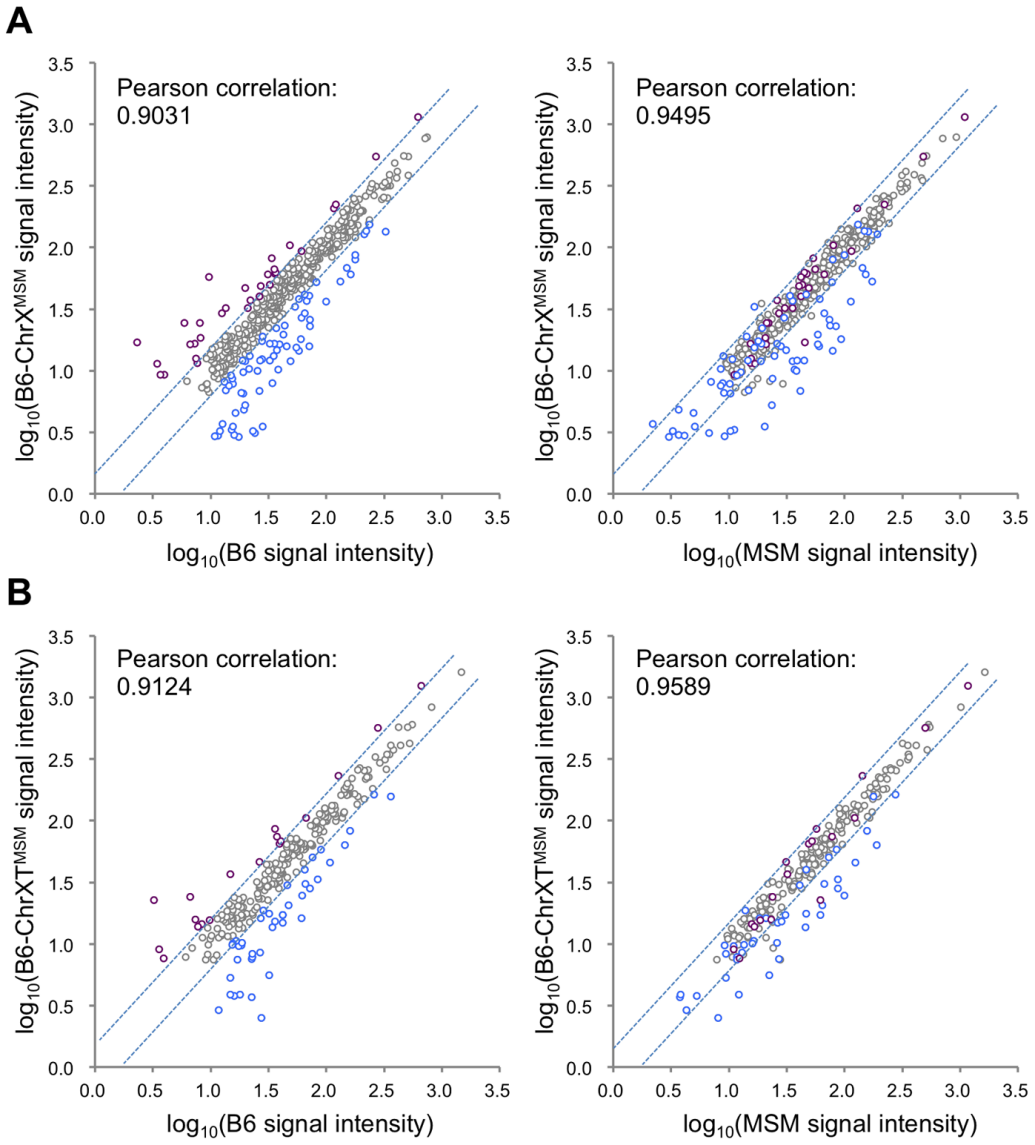
Comparison of signal intensities of MSM-derived X-linked transcripts in two X chromosome substitution strains with those in B6 or MSM. (A) Y-axis is the logarithm transformed signal intensity of B6-ChrX^MSM^, and X-axis is the logarithm transformed signal intensity of B6 (left) or MSM (right). Transcripts upregulated or downregulated in B6-ChrX^MSM^ testes relative to those in B6 are indicated by purple and blue circles, respectively. Dotted blue lines represent borders of a 1.5-fold change for upregulation or downregulation. Note that almost all upregulated transcripts were converged at a less than 1.5-fold change relative to the intensity of MSM. (B) Comparison of two strains for the logarithm transformed signal intensities of distal-X-linked transcripts in B6-ChrXT^MSM^ testes.

Next, to examine whether MSM-derived *cis*-regulatory elements are responsible for the differential expression in B6-ChrX^MSM^, we compared the expression signal intensities of all X-linked genes expressed in B6-ChrX^MSM^ testes with that in B6 or MSM testes. The Pearson correlation coefficient of B6-ChrX^MSM^ to B6 and B6-ChrX^MSM^ to MSM was 0.9031 and 0.9495, respectively (Figure 2A). We tested whether the correlation coefficient of B6-ChrX^MSM^ to MSM is significantly larger than that of B6-ChrX^MSM^ to B6 or not. We applied the logarithm transformation of the data to approximate the distribution of the data by the normal distribution and then obtained the approximate *P*-value by the test using the Fisher's z-transformation. The results showed that the correlation coefficient of B6-ChrX^MSM^ to MSM was significantly larger than that of B6-ChrX^MSM^ to B6 (*P* = 6.97×10^−8^). When the signal intensity of B6-ChrX^MSM^ was compared with that of MSM, 93.5% (29/31) of upregulated transcripts (indicated in purple dots in Figure 2A) and 45.7% (32/70) of downregulated transcripts (indicated in blue dots in Figure 2A) were converged in a less than 1.5-fold change. This result suggests that these transcripts might reflect transcriptional regulation by MSM-derived *cis*-regulatory elements on the X chromosome.

### Differential Expression of Genes on the Distal Half of the X Chromosome in B6-ChrXT^MSM^ Testes

**Figure 3:**
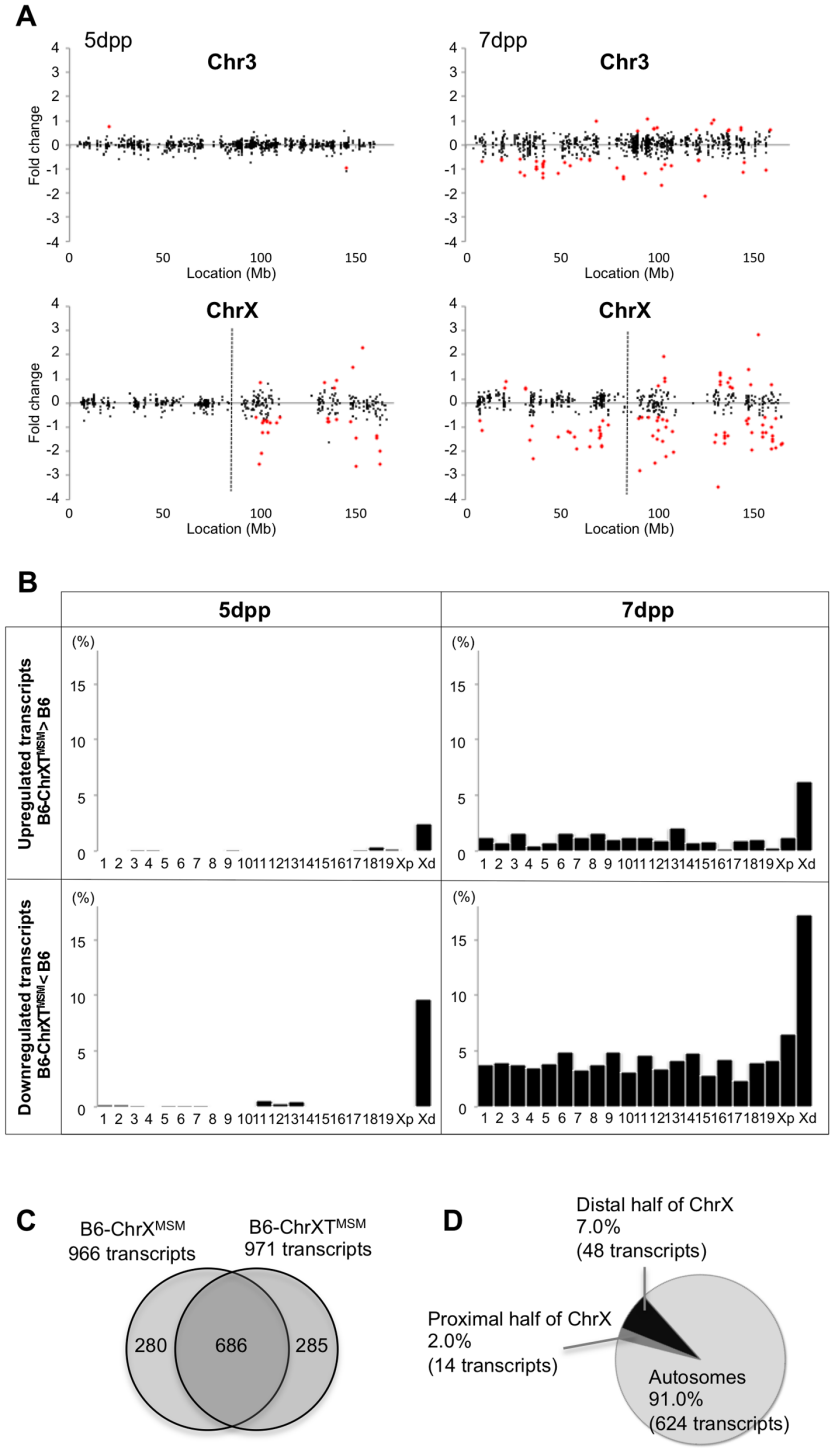
Differential expression of MSM-derived genes in B6-ChrXT^MSM^ testes. (A) Gene expression on control chromosome 3 and the X chromosome in B6-ChrXT^MSM^ testes. Vertical dotted line in the bottom panels indicates the boundary of recombination. The MSM-derived region is distal to the boundary. Note that the variance of fold change in the MSM-derived region was larger than that in the other chromosomal region. Transcripts in red showed a significantly different expression level by the Benjamini-Hochberg FDR corrected moderate *t*-test (*P*<0.05; fold change ≥1.50). (B) Frequency of upregulated and downregulated transcripts for each chromosome in B6-ChrXT^MSM^ testes. Chromosomes are indicated below the graphs. Xp and Xd represent B6-derived proximal and MSM-derived distal regions on the X chromosome, respectively. Significant enrichment of differentially expressed transcripts was observed in the distal X-chromosomal region. Differential expression was predominately genome wide at 7 dpp. (C) Venn diagram of differentially expressed transcripts in B6-ChrX^MSM^ and B6-ChrXT^MSM^ testes at 7 dpp. A large proportion of differentially expressed transcripts were common in B6-ChrX^MSM^ and B6-ChrXT^MSM^ samples. (D) Genome location of the common differentially expressed transcripts.

The B6-ChrXT^MSM^ strain has a MSM-derived genome between 86,497,454 and 165,344,914 bp of the X chromosome. Consequently, we could investigate how the subspecies origin of the genomic region is attributable to the differential expression of the X chromosome. We performed transcriptional profiling of B6-ChrXT^MSM^ testes at 5 dpp. Of the 697 probe sets on the distal half of the X chromosome, 176 polymorphic probe sets were excluded from the analysis. Of the remaining 43,888 probe sets for autosomal and X chromosomal transcripts, 17,109 were used after filtering by the expression signal intensity. We plotted base-2 logarithms of the fold changes of transcript expression levels in B6-ChrXT^MSM^ testes relative to those in the B6 strain. The variance of fold changes of the transcripts from genes located on the distal half of the X chromosome was significantly larger compared with that of other chromosomal regions including the proximal half of the X chromosome (Ansari-Bradley test, *P*≤1.00×10^−16^; Figures 3A and 5). The upregulated and downregulated transcripts were detected by 2.51% (6/239) and 9.62% (23/239) of the probe sets, respectively, for the distal half of the X chromosome, whereas differentially expressed transcripts for autosomes and the proximal half of the X chromosome were detected at a apparently lower frequency (Figure 3B and Table 2).

**Table 2:** Frequency of downregulated and upregulated transcripts for each chromosome in B6-ChrXT^MSM^ testes.

Chr	All probe sets	5 dpp			7 dpp		
		expressed probe sets	upregulated (%)	downregulated (%)	expressed probe sets	upregulated (%)	downregulated (%)
1	2839	1169	0 (0.00)	2 (0.17)	1250	15 (1.20)	47 (3.76)
2	3540	1383	0 (0.00)	2 (0.14)	1503	11 (0.73)	60 (3.99)
3	2292	890	1 (0.11)	1 (0.11)	944	15 (1.59)	36 (3.81)
4	2648	1058	1 (0.09)	0 (0.00)	1154	5 (0.43)	40 (3.47)
5	2769	1070	0 (0.00)	1 (0.09)	1155	9 (0.78)	45 (3.90)
6	2472	838	0 (0.00)	1 (0.12)	918	15 (1.63)	45 (4.90)
7	2941	1038	0 (0.00)	1 (0.10)	1135	14 (1.23)	38 (3.35)
8	2145	847	0 (0.00)	0 (0.00)	917	15 (1.64)	35 (3.82)
9	2349	918	1 (0.11)	0 (0.00)	996	10 (1.00)	49 (4.92)
10	2088	804	0 (0.00)	0 (0.00)	861	11 (1.28)	27 (3.14)
11	3410	1400	0 (0.00)	8 (0.57)	1493	19 (1.27)	70 (4.69)
12	1709	678	0 (0.00)	2 (0.29)	731	7 (0.96)	25 (3.42)
13	1804	679	0 (0.00)	3 (0.44)	718	15 (2.09)	30 (4.18)
14	1624	612	0 (0.00)	0 (0.00)	661	5 (0.76)	32 (4.84)
15	1782	686	0 (0.00)	0 (0.00)	740	6 (0.81)	21 (2.84)
16	1543	597	0 (0.00)	0 (0.00)	655	1 (0.15)	28 (4.27)
17	1919	775	1 (0.13)	0 (0.00)	836	8 (0.96)	20 (2.39)
18	1287	529	2 (0.38)	0 (0.00)	552	6 (1.09)	22 (3.99)
19	1514	602	1 (0.17)	0 (0.00)	652	2 (0.31)	27 (4.14)
proX*	687	297	0 (0.00)	0 (0.00)	323	4 (1.24)	21 (6.50)
disX*	521	239	6 (2.51)	23 (9.62)	255	16 (6.27)	44 (17.25)

*ProX and disX indicate the proximal and distal half of the X chromosome, respectively.

We next performed microarray analysis of testes from a congenic strain in which only a 37.8 Mb interval between 125,512,711 and 163,344,520 bp on a more distal region of the X chromosome was substituted by the MSM-derived genome in the B6 background. We observed similar differential expression only in the MSM-derived genome, as was observed in B6-ChrX^MSM^ and B6-ChrXT^MSM^ strains (Figure S6). This result indicated that the differential expression is strictly restricted to the genomic region derived from the MSM strain.

When we performed transcriptional profiling of B6-ChrXT^MSM^ testes at 7 dpp, 18,449 probe sets remained after filtering by the expression signal intensity. Consistent with B6-ChrX^MSM^ testes, we found genome-wide differential expression in B6-ChrXT^MSM^ testes at 7 dpp was more obvious compared with that at 5 dpp (Ansari-Bradley test, autosomes and the proximal half of the X chromosome, *P*≤1.00×10^−16^, the distal half of the X chromosome, *P* = 2.61×10^−4^; Figures 3A and S7). The frequency of the differentially expressed transcripts detected by the probe sets for each chromosome is shown in Figure 3B and Table 2. Such transcripts in 5- and 7-day-old B6-ChrXT^MSM^ testes are listed in Tables S5 and S6, respectively. At 7 dpp, differentially expressed transcripts in B6-ChrXT^MSM^ testes largely overlapped with those in B6-ChrX^MSM^ testes (70.6%, 686/971) (Figure 3C). They consisted of not only transcripts from the distal half of the X chromosome (7.0%), but also those from autosomal regions (91.0%) (Figure 3D and Table S7). This finding implies that differential expression in the distal half of the X chromosome subsequently affects genome-wide transcription at 7 dpp.

We also compared the signal intensity of all transcripts from the distal-half of the X chromosome in B6-ChrXT^MSM^ testes with that in B6 or MSM strains. The Pearson correlation coefficient of B6-ChrXT^MSM^ to B6 was 0.9124, whereas that of B6-ChrXT^MSM^ to MSM was 0.9589 (Figure 2B). The correlation coefficient of B6-ChrXT^MSM^ to MSM was significantly larger than that of B6-ChrXT^MSM^ to B6 by the test using the Fisher's z-transformation (*P* = 4.02×10^−6^). When the expression in B6-ChrXT^MSM^ was compared with that in MSM, 77.8% (14/18) and 40.5% (17/42) of upregulated (purple dots in Figure 2B) and downregulated transcripts (blue dots in Figure 2B), respectively, were converged in a less than 1.5-fold change.

### Restoration of the Differential Expression in B6-ChrXT^MSM^ Testes by Introduction of Chromosome 1 from the MSM Strain

**Figure 4:**
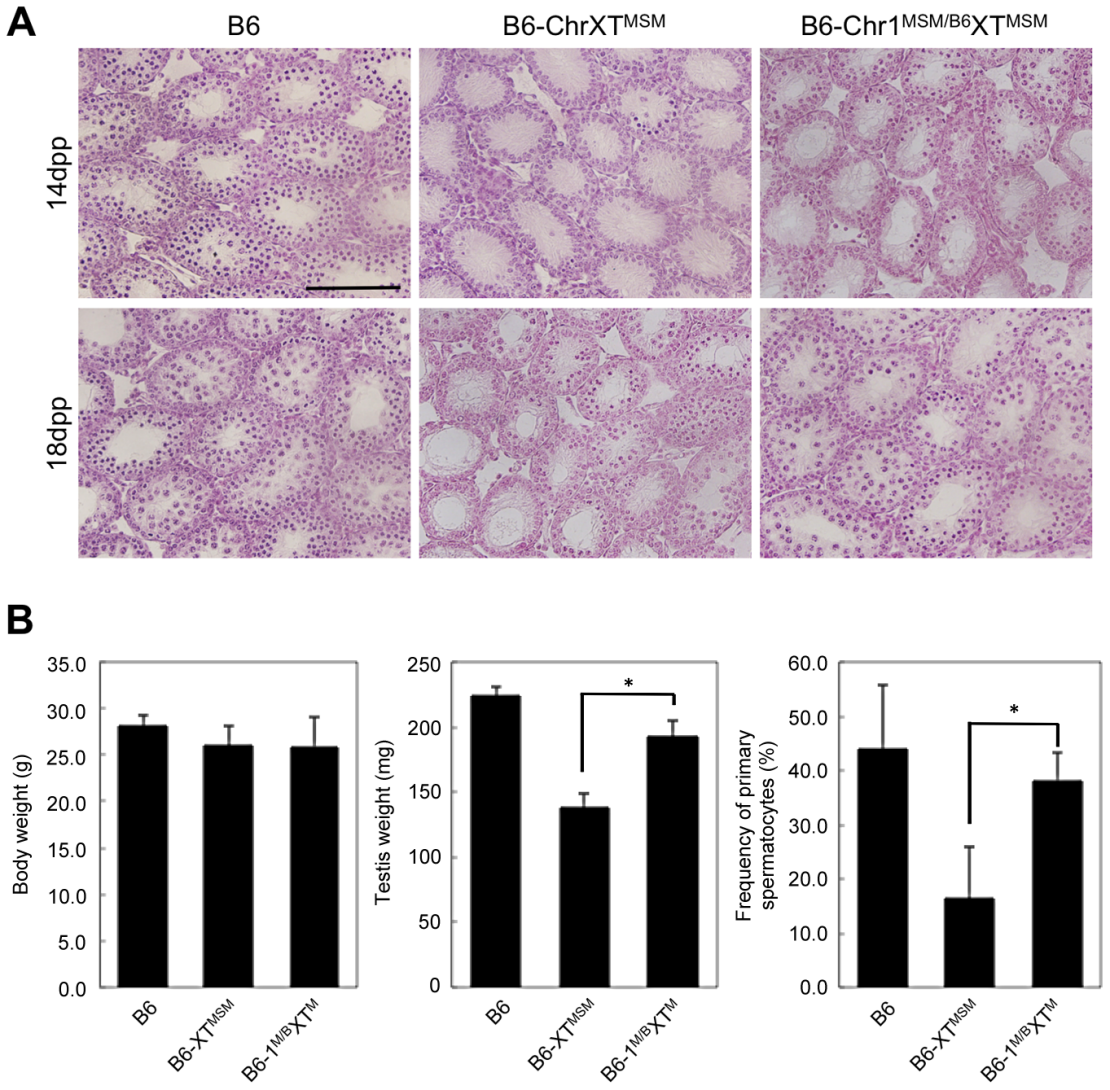
Restoration of reproductive phenotypes in B6-Chr1^MSM/B6^XT^MSM^ testes. (A) Histology of the testis of each strain. Meiotic spermatocytes were rarely observed in B6-Chr1^MSM/B6^XT^MSM^ testes similar to that in B6-ChrXT^MSM^ testes at 14 dpp. Note that abundant meiotic spermatocytes were located in the seminiferous tubules of B6-Chr1^MSM/B6^XT^MSM^ testes, as observed in the control B6 testes at 18 dpp. Scale bar: 250 µm (B) Restoration of testis weight and the frequency of primary spermatocytes in B6-Chr1^MSM/B6^XT^MSM^ males. Body and paired testis weights of adult males and the frequency of spermatocytes in the testes of 18 dpp males are indicated. *P<0.01, two-tailed Student's *t*-test.

To assign a chromosome that interacts with X chromosomal genes and is responsible for the genetic incompatibility in B6-ChrXT^MSM^ males, we produced F1 male progeny generated from crosses of B6-ChrXT^MSM^ females with males of other chromosomal substitution strains, and investigated their reproductive phenotypes. We generated crosses with a total of 22 chromosome substitution strains of which 13 crosses showed significant restoration of testis weight (Bonferroni-corrected *P*<0.05 by the two-tailed Student's *t*-test; Figure S8). Among F1 male progeny from crosses with chromosomal substitution strains, (B6-ChrXT^MSM^×B6-Chr1^MSM^)F1 males (hereafter abbreviated as B6-Chr1^MSM/B6^XT^MSM^) exhibited the most effective restoration of testis weight (Figure S8). To determine whether the restoration of testis weight is caused by proper spermatogenesis or other reasons including hyperplasia of Leydig cells, we investigated the phenotype of B6-Chr1^MSM/B6^XT^MSM^ testes. The testicular histology showed that meiotic spermatocytes observed in B6-Chr1^MSM/B6^XT^MSM^ testes were not abundant as observed in B6-ChrXT^MSM^ testes at 14 dpp, but spermatocytes were increased in B6-Chr1^MSM/B6^XT^MSM^ testes at 18 dpp (Figure 4A). This result indicated that the progression of spermatogenesis in B6-Chr1^MSM/B6^XT^MSM^ males is delayed at the early stage, but becomes similar to that of the B6 strain at a later stage. Immunostaining analysis showed that the frequency of spermatocytes was restored significantly in B6-Chr1^MSM/B6^XT^MSM^ testes at 18 dpp (Figure 4B).

**Figure 5:**
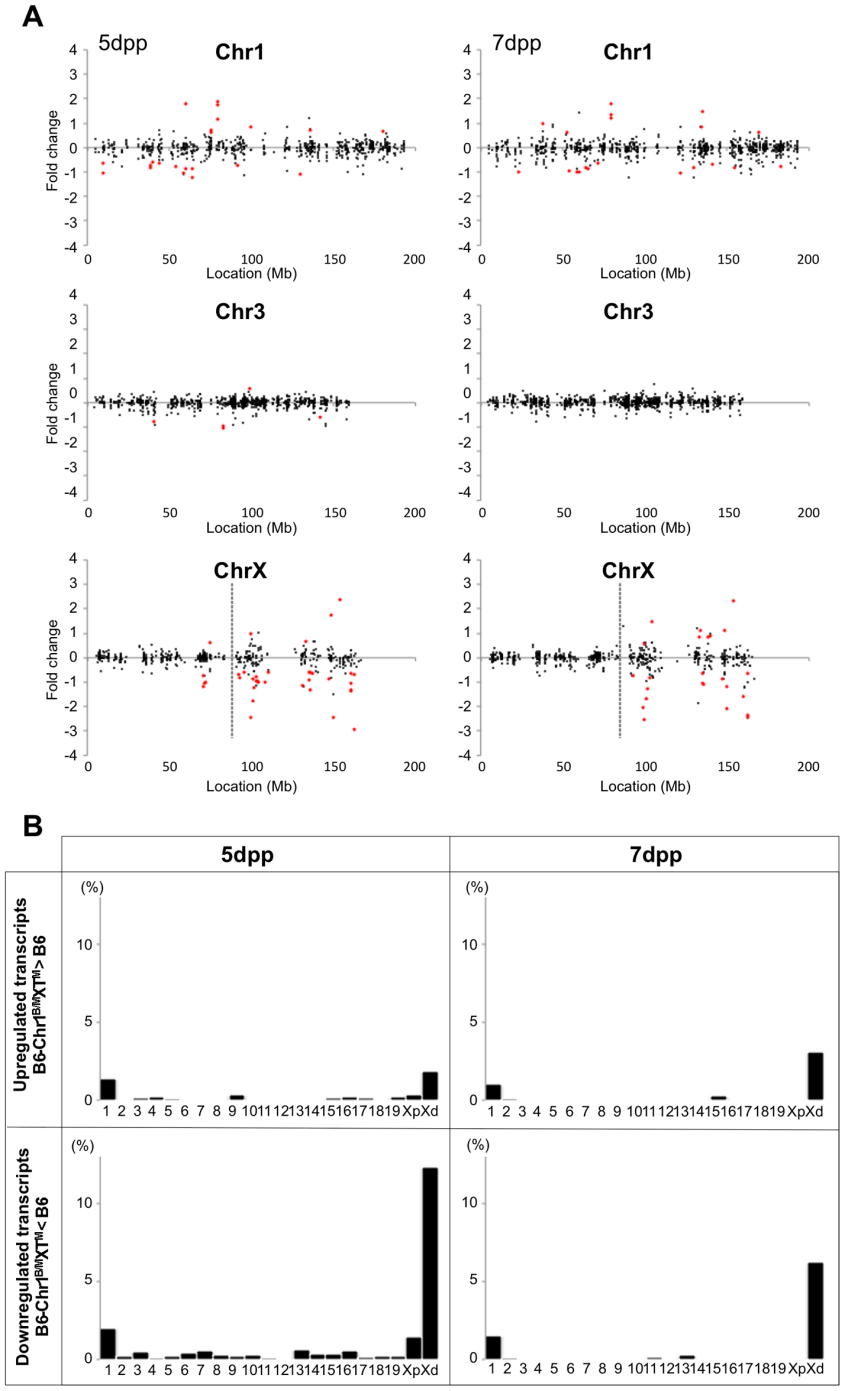
Restoration of genome-wide differential gene expression in B6-Chr1^MSM/B6^XT^MSM^. (A) Gene expression on chromosome 1, 3, and the X chromosome in B6-Chr1^MSM/B6^X^MSM^ testes. Vertical dotted line in the bottom panels indicates the boundary between B6- and MSM-derived genomic regions. Note that the variance of fold changes was extremely large on chromosome 1 and the distal half of the X chromosome. Transcripts in red showed significantly different expression by the Benjamini-Hochberg FDR corrected moderate *t*-test (*P*<0.05; fold change ≥1.50). (B) Frequency of upregulated and downregulated transcripts for each chromosome in B6-Chr1^MSM/B6^XT^MSM^ testes. Chromosomes are indicated below the graphs. Xp and Xd represent B6-derived proximal and MSM-derived distal regions on the X chromosome, respectively. Genome-wide differential expression was not observed in B6-Chr1^MSM/B6^XT^MSM^ testes at 7 dpp.

To examine the timing of spermatogenesis restoration in B6-Chr1^MSM/B6^XT^MSM^ testes, we performed transcriptional profiling by microarray analysis of testes from B6-Chr1^MSM/B6^XT^MSM^ males at 5 and 7 dpp. Because the B6-Chr1^MSM/B6^XT^MSM^ strain was heterozygous for B6 and MSM alleles on chromosome 1, polymorphic probe sets on chromosome 1 were excluded from this microarray analysis. Of the remaining 42,781 probe sets, 15,377 probe sets were used after filtering by the expression signal intensity using 5 dpp samples. Fold changes of expression signals in B6-Chr1^MSM/B6^XT^MSM^ testes relative to that in the B6 strain revealed that the genes on the distal half of the X chromosome were expressed differentially as was observed in B6-ChrXT^MSM^ testes (Figures 5A and 5B, Table 3, and Figure S9). The differentially expressed transcripts in 5 dpp B6-Chr1^MSM/B6^XT^MSM^ testes are listed in Table S8.

**Table 3:** Frequency of downregulated and upregulated transcripts for each chromosome in B6-Chr1^MSM/B6^X^MSM^ testes.

Chr	All probe sets	5 dpp			7 dpp		
		expressed probe sets	upregulated (%)	downregulated (%)	expressed probe sets	upregulated (%)	downregulated (%)
1	1768	646	9 (1.39)	13 (2.01)	795	8 (1.01)	12 (1.51)
2	3540	1276	0 (0.00)	3 (0.24)	1578	1 (0.06)	1 (0.06)
3	2292	831	1 (0.12)	4 (0.48)	977	0 (0.00)	0 (0.00)
4	2645	981	2 (0.20)	1 (0.10)	1204	0 (0.00)	0 (0.00)
5	2767	977	1 (0.10)	2 (0.20)	1184	0 (0.00)	0 (0.00)
6	2472	769	0 (0.00)	3 (0.39)	959	0 (0.00)	0 (0.00)
7	2941	965	0 (0.00)	5 (0.52)	1175	0 (0.00)	0 (0.00)
8	2145	781	0 (0.00)	2 (0.26)	951	0 (0.00)	0 (0.00)
9	2342	844	3 (0.36)	2 (0.24)	1038	0 (0.00)	0 (0.00)
10	2088	744	0 (0.00)	2 (0.27)	911	0 (0.00)	0 (0.00)
11	3410	1299	0 (0.00)	1 (0.08)	1556	0 (0.00)	2 (0.13)
12	1709	635	0 (0.00)	0 (0.00)	763	0 (0.00)	0 (0.00)
13	1789	633	0 (0.00)	4 (0.63)	742	0 (0.00)	2 (0.27)
14	1624	578	0 (0.00)	2 (0.35)	685	0 (0.00)	0 (0.00)
15	1782	625	1 (0.16)	2 (0.32)	769	2 (0.26)	0 (0.00)
16	1543	555	1 (0.18)	3 (0.54)	671	0 (0.00)	0 (0.00)
17	1915	714	1 (0.14)	1 (0.14)	872	0 (0.00)	0 (0.00)
18	1287	481	0 (0.00)	1 (0.21)	573	0 (0.00)	0 (0.00)
19	1514	543	1 (0.18)	1 (0.18)	676	0 (0.00)	0 (0.00)
proX*	687	281	1 (0.36)	4 (1.42)	332	0 (0.00)	0 (0.00)
disX*	521	219	4 (1.83)	27 (12.33)	258	8 (3.10)	16 (6.20)

*ProX and disX indicate the proximal and distal half of the X chromosome, respectively.

**Figure 6:**
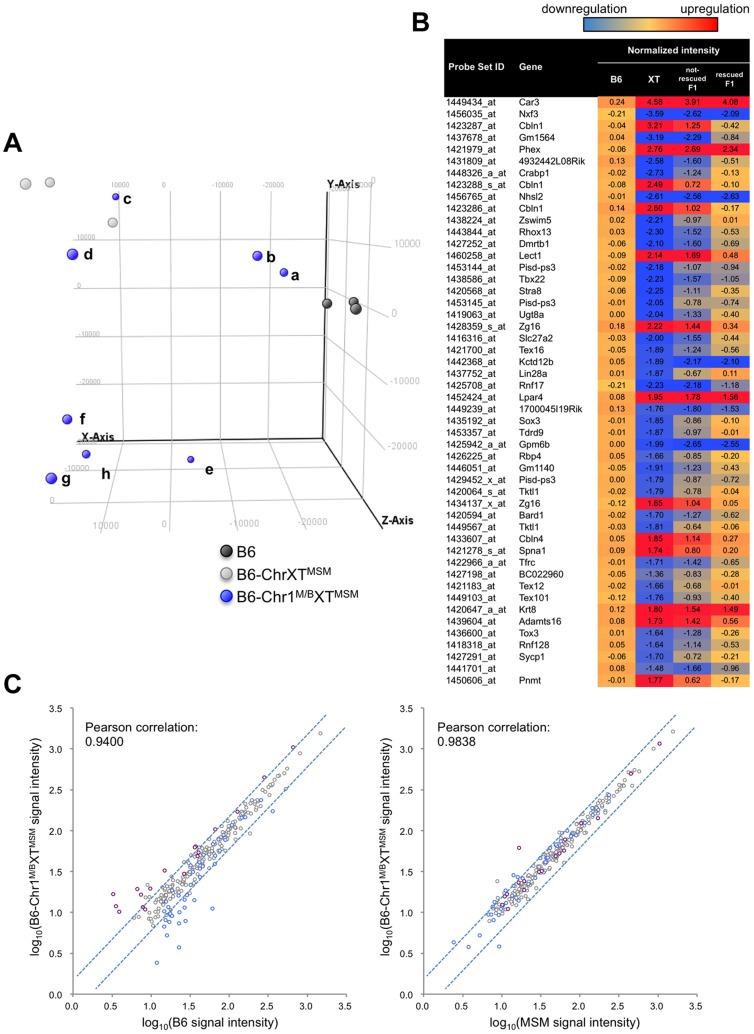
Transcriptional profiling of B6-Chr1^MSM/B6^XT^MSM^ testes. (A) PCA score plots using global gene expression profiles of B6, B6-ChrXT^MSM^ and B6-Chr1^MSM/B6^XT^MSM^ individuals. Coordinate values of B6-Chr1^MSM/B6^XT^MSM^ individuals (a∼h) were dispersed between two clustered values of the B6 and B6-ChrXT^MSM^ individuals. Note that two B6-Chr1^MSM/B6^XT^MSM^ individuals (a and b) were located near the B6 individuals. X-axis = PC1: PCA Component 1 (19.6% variance); Y-axis = PC2: PCA Component 2 (17.9% variance); Z-axis = PC3: PCA Component 3 (11.1% variance). (B) Top 50 ranking of transcripts in B6-ChrXT^MSM^ testes with expression levels that were different from those in B6 testes. Heatmap shows the transcripts that were downregulated (blue) or upregulated (red) relative to those in B6. Each number indicates the fold change of expression relative to B6 controls. Two types of B6-Chr1^MSM/B6^XT^MSM^, restored and not-restored types, showed different levels of restoration. (C) Comparison of the logarithm transformed signal intensities of distal-X-linked transcripts in B6-Chr1^MSM/B6^XT^MSM^ testes with those in B6 or MSM. Y-axis is signal intensity of B6-Chr1^MSM/B6^XT^MSM^, and X-axis is signal intensity of B6 (left) or MSM (right). Upregulated or downregulated transcripts in B6-ChrXT^MSM^ testes relative to B6 are indicated by purple and blue circles, respectively. Dotted blue lines represent the borders of a 1.5 fold change for upregulation or downregulation.

When we performed the transcriptional profiling with 7 dpp B6-Chr1^MSM/B6^XT^MSM^ testes, we found that the expression patterns were noticeably variable among the individuals. We conducted principal component analysis (PCA), as implemented in GeneSpring GX software, using expression data for all genes of three individuals each of B6 and B6-ChrXT^MSM^, and eight individuals of B6-Chr1^MSM/B6^XT^MSM^. The results showed that the coordinate values were clustered for B6 and B6-ChrXT^MSM^ individuals (Figure 6A). In contrast, the coordinate values of the eight B6-Chr1^MSM/B6^XT^MSM^ individuals were largely scattered between the clusters of B6 and B6-ChrXT^MSM^. Two B6-Chr1^MSM/B6^XT^MSM^ individuals (a and b in Figure 6A) were classified into the same cluster of B6, suggesting that genome-wide transcriptional regulation was restored in these individuals. The other two B6-Chr1^MSM/B6^XT^MSM^ individuals (c and d in Figure 6A) were rather close to the cluster of B6-ChrXT^MSM^, indicating that they remained in the original state. Another four B6-Chr1^MSM/B6^XT^MSM^ individuals belonged to neither B6 nor B6-ChrXT^MSM^ clusters.

To characterize the restoration of B6-Chr1^MSM/B6^XT^MSM^ testes in more detail, the B6-Chr1^MSM/B6^XT^MSM^ individuals were classified as “restored” or “non-restored” types. Individuals a, b, and e belonged to the restored type and were relatively close to the B6 cluster on the x-axis of principal component 1. The remaining individuals belonged to the non-restored type. Of the 42,781 probe sets, 18,669 remained for analysis after filtering by the expression signal intensity. Notably, the high degree of genome-wide differential expression that occurred in B6-ChrX^MSM^ and B6-ChrXT^MSM^ testes at 7 dpp was not observed in the restored type of B6-Chr1^MSM/B6^XT^MSM^ individuals (Figure 5A and 5B, Table 3, and Figure S10). The Ansari-Bradley test showed that the variance of the fold changes in restored B6-Chr1^MSM/B6^XT^MSM^ individuals was significantly smaller than that in B6-ChrXT^MSM^ individuals, which was irrespective of the chromosomal regions (autosomes and the proximal half of the X chromosome, *P*≤1.00×10^−16^; the distal half of the X chromosome, *P* = 5.25×10^−5^). This result suggested that the gene expression pattern of the restored B6-Chr1^MSM/B6^XT^MSM^ individuals was recovered to some extent from the differential expression toward the B6 type. When we examined the expression pattern of the top 50 differentially expressed genes in B6-ChrXT^MSM^ testes at 7 dpp, we found that the expression levels of these genes were considerably restored in B6-Chr1^MSM/B6^XT^MSM^ testes (Figure 6B). In contrast, when the fold changes of expression for transcripts in non-restored B6-Chr1^MSM/B6^XT^MSM^ males relative to B6 males were plotted, the variance of the transcripts remained to be similar to B6-ChrXT^MSM^ males (Figure S11). Although B6-Chr1^MSM/B6^XT^MSM^ individuals showed variable expression profiles, all tested adult B6-Chr1^MSM/B6^XT^MSM^ individuals (N = 14) indicated the restoration of testis weight (Figure S8). Thus, theses observations suggested that the developmental stage at 7 dpp might be a transient period of change from the differential expression profile of B6-Chr1^MSM/B6^XT^MSM^ testes to the B6 type.

Although the general expression profile was re-established in the restored type of B6-Chr1^MSM/B6^XT^MSM^ individuals, transcripts from the distal half of the X chromosome appeared to remain in a differentially expressed state (Figure 5A and 5B, and Table S9). Next, we compared the signal intensity of transcripts of B6-Chr1^MSM/B6^XT^MSM^ on the distal half of the X chromosome with that of B6 or MSM. The Pearson correlation coefficient of B6-Chr1^MSM/B6^XT^MSM^ to B6 was 0.9400, and that of B6-Chr1^MSM/B6^XT^MSM^ to MSM was 0.9838 (Figure 6C). The correlation coefficient of B6-Chr1^MSM/B6^XT^MSM^ to MSM was significantly larger than that of B6-Chr1^MSM/B6^XT^MSM^ to B6 by the test using the Fisher's z-transformation (*P* = 1.27×10^−14^). This result suggests that genetic incompatibilities in the transcriptional regulation of differentially expressed X-linked genes are resolved to some extent by MSM alleles of upstream regulatory genes on chromosome 1.

Interestingly, we noticed a high frequency of differentially expressed genes on chromosome 1 as compared to those on other autosomes in B6-Chr1^MSM/B6^XT^MSM^ individuals at both 5 and 7 dpp, although they carried MSM alleles heterozygously throughout chromosome 1 (Figures 5A and 5B). The Ansari-Bradley test showed that the variance of fold changes of transcripts from chromosome 1 was significantly larger than that of other chromosomal regions except the distal X chromosome (5 dpp, *P*≤1.00×10^−16^; 7 dpp, *P*≤1.00×10^−16^). This finding suggests that genes in the MSM-derived genome on chromosome 1 are also differentially expressed in B6-Chr1^MSM/B6^XT^MSM^ testes.

To re-examine the expression pattern of X-linked genes in B6-ChrXT^MSM^ and B6-Chr1^MSM/B6^XT^MSM^ testes, the expression levels of selected genes were measured by real-time quantitative RT-PCR. The expression levels of eight genes that locate near by the QTL responsible for reduced testis weight were significantly low in the B6-ChrXT^MSM^ testes than in the B6 testes, which was consistent with the microarray data (Figure S12). The expression levels of these genes were restored in B6-Chr1^MSM/B6^XT^MSM^ testes, and their expression levels tended to shift toward those of MSM testes rather than B6 testes. The eight genes included three candidate genes responsible for genetic incompatibility: TAF7-like RNA polymerase II, TATA box binding protein (TBP)-associated factor (*Taf7l*), and two nuclear mRNA export factors (*Nxf2* and *Nxf3*).

To identify the genes responsible for the restoration of differential expression in B6-Chr1^MSM/B6^XT^MSM^ testes, we performed QTL analysis. We produced F1 male progeny from crosses between B6-Chr1^MSM^ females and wild-type B6 males. Then, the (B6-Chr1^MSM^×B6)F1 males were crossed with B6-ChrXT^MSM^ females to obtain 314 male progeny for the QTL analysis (Figure S13A). Because the male progeny had recombination at various sites in chromosome 1 between B6 and MSM, we could map the QTLs affecting testis weight. As a result, we found continuously high LOD scores over the large region of chromosome 1 by single marker analysis and interval mapping (Figure S13B and S13C). The highest peak was observed at 64.5 Mb by the interval mapping (LOD score = 21.8). This indicates that at least one QTL responsible for the genetic incompatibility are located possibly at the region of 40–80 Mb on chromosome 1.

## Discussion

We clearly show that approximately 20% of genes located in MSM-derived X chromosomal regions are differentially expressed in the genetic background of the B6 strain. The most important finding in this study is that such differential expression in prepubertal testes occurs prior to the reproductive phenotype of the X chromosome substitution strains. Furthermore, perturbation of genome-wide gene expression possibly caused by the differential X-linked gene expression might enhance the reproductive phenotype. Thus, the ultimate phenotype of the reproductive isolation is the sum of deleterious effects by the differential expression of many genes.

Previous studies showed extensive overexpression of X-chromosomal genes in testes of sterile F1 males generated from cross of *M. m. musculus* females and *M. m. domesticus* males due to a disruption of MSCI [41], [42]. By contrast, our study showed that the differential expression of MSM-derived genes in the X chromosome substitution strains occurs at premeiotic stage of spermatogenesis, and the differential expression is bidirectional, upregulation and downregulation. Moreover, it was observed not only for the X chromosomal genes but also for genes on chromosome 1. Thus, mechanisms underlying the overexpression of X-chromosomal genes in the F1 males might be different from that in the X chromosome substitution strains.

The cell population in seminiferous tubules of the prepubertal testis consists of undifferentiated spermatogonia called spermatogonial stem cells (SSCs), differentiated spermatogonia, and their supporting Sertoli cells. To identify which cell type showed differential expression in the B6-ChrX^MSM^ (or B6-ChrXT^MSM^) testis, we referred to a previous study by Yang *et al.* [35]. In their study, microarray analyses were carried out using OCT4-positive SSCs and OCT4-negative cells, including differentiated spermatogonia and somatic cells, which were isolated from 7 dpp testes. OCT4 is an important transcription factor involved in maintenance of stem cell pluripotency. We found that the differentially expressed transcripts in the X chromosome substitution strains were present in the gene lists enriched in both OCT4-positive and OCT4-negative cells (Figure S14), suggesting that the differential gene expression occurs in both SSCs and non-SSC cells. Interestingly, all differentially expressed transcripts that were enriched in SSCs were downregulated, whereas those enriched in non-SSC cells showed preferential upregulation (Figure S14). The implication of the observed skew in differential expression is unknown at present. We could not exclude the possibility of delayed spermatogonial development, though the testes appeared to be similar histology in B6 and the X chromosome substitution strains, which might influence the transcription profiles. To refer to the generality of misregulation in each type of testicular cells, further analysis using isolated cell population will be required.

**Figure 7:**
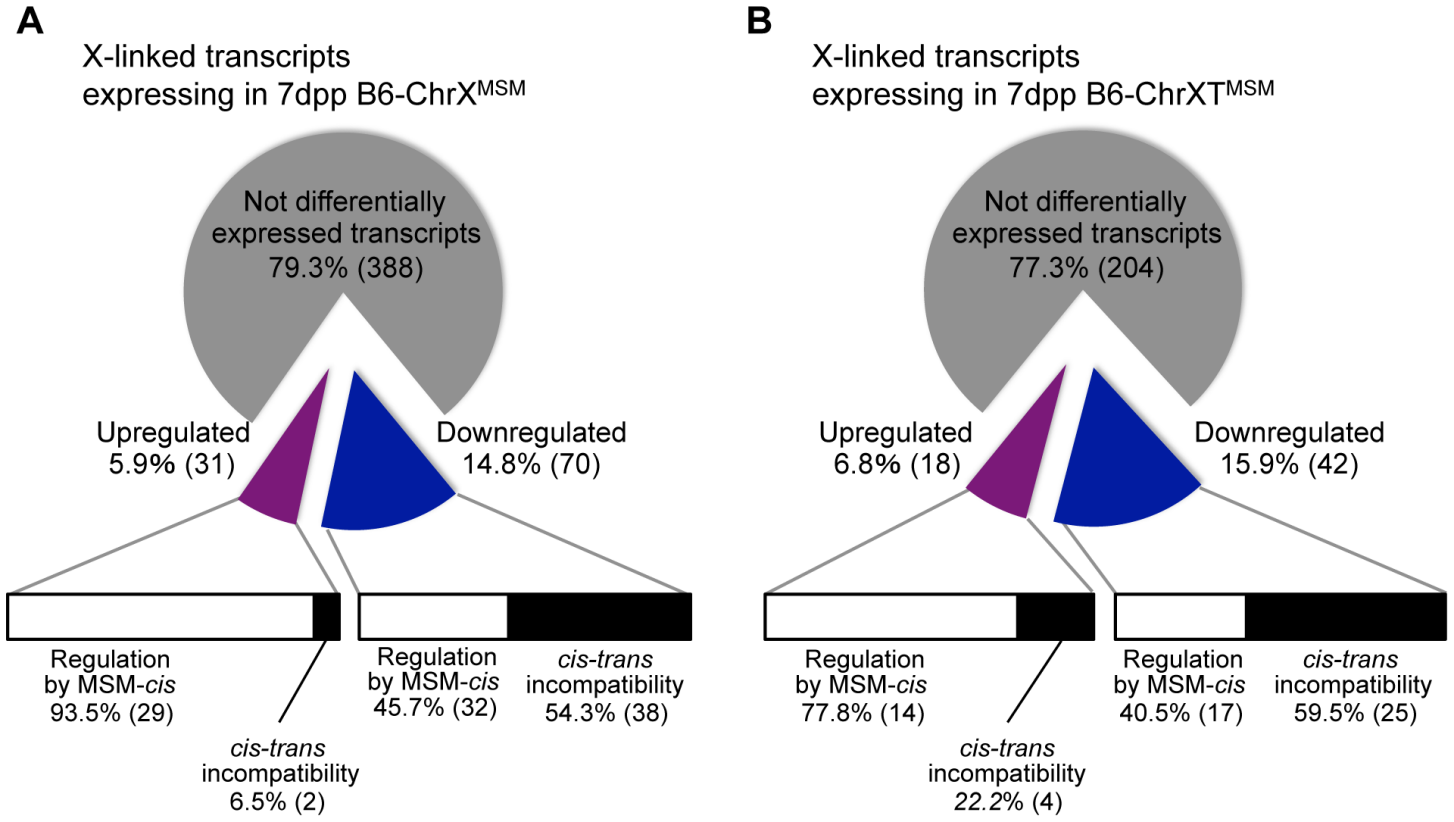
Classification of MSM-derived X-linked transcripts in B6-ChrX^MSM^ and B6-ChrXT^MSM^. Pie charts depict the frequency of upregulated (purple) and downregulated (blue) transcripts in B6-ChrX^MSM^ (A) and B6-ChrXT^MSM^ (B) testes relative to B6 expression at 7 dpp. Horizontal bars depict the proportion for each type of transcriptional regulation; differential expression by MSM-derived *cis*-regulatory elements (white bar) and misregulation caused by the genetic incompatibility between *cis*-regulatory elements and *trans*-acting factors (black bar).

For transcripts from X-linked genes in B6-ChrX^MSM^ and B6-ChrXT^MSM^, approximately 77–79% transcripts were not differentially expressed relative to the B6 strain, implying that the transcriptional regulation of these genes is conserved between MSM and B6 strains (Figure 7A and 7B). Approximately 15–16% of the transcripts were downregulated, while the remaining 6–7% showed upregulation. By comparing the expression signal intensities of differentially expressed genes in X chromosome substitution strains with those of the same genes in the MSM strain, we found that these genes could be classified into two types. The first type showed expression levels comparable to those in the MSM strain, whereas the second type showed expression levels that were different from those in the MSM strain (Figure 7A and 7B and Table S10 and S11). The first type likely reflects transcriptional regulation by MSM-derived *cis*-regulatory elements on the X chromosome. The second type might reflect the transcriptional misregulation possibly caused by the incompatibility between B6-derived *trans*-acting regulators and MSM-derived *cis*-regulatory elements for X-linked target genes. Notably, the proportions of these two types were significantly different between upregulated and downregulated transcripts. In B6-ChrX^MSM^ (or B6-ChrXT^MSM^), 80–90% of upregulated transcripts belonged to the first type (Figure 7A and 7B). In contrast, more than 50% of downregulated transcripts belonged to the second type. It is plausible that the genetic incompatibility of the transcriptional regulation readily causes a decrease of gene expression rather than an increase.

In this study, we found that a number of genes on the MSM-derived X chromosome and chromosome 1 were differentially expressed in the genetic background of the B6 strain. This observation suggests that genome-wide transcriptional (or post-transcriptional) regulation has evolutionarily diverged during mouse subspeciation, and that the diverged expression is not specific to the X chromosome, but rather a general phenomenon observed for genes in substituted chromosomes. Despite the diverged expression of genes on chromosome 1, the parental B6-Chr1^MSM^ strain does not exhibit any reproductive phenotypes [43], and GO analysis showed that differentially expressed genes were not preferentially categorized into reproductive GO classes (data not shown). Male reproductive genes preferentially accumulate on the X chromosome, because beneficial X-linked genes are readily selected because of the hemizygosity in males [8], [44], [45]. Moreover, genes expressed during premeiotic stages are not subjected to sex chromosome inactivation. All these conditions accelerate accumulation of male reproduction-related genes on the X chromosome, which are expressed at the early stage of spermatogenesis. Consequently, substitution of an X chromosome from different subspecies might have disproportionally more significant effects on premeiotic spermatogenesis than that of autosomes.

Although a set of differentially expressed genes on the X chromosome and autosomes is responsible for the reproductive phenotype of X chromosome substitution strains, some genes may exert predominant effects on spermatogenic and cellular processes. Our QTL analysis of the reduced testis weight detected high LOD score on the distal one-third of the X chromosome. This region contains multiple differentially expressed genes that are known to be involved in spermatogenesis and housekeeping functions. We then explored X-linked genes that are differentially expressed in the testes of X chromosome substitution strains relative to that in B6 testes, and are thought to predominantly affect the testis weight. Based on the functional gene annotations, we focused on three genes in the relevant region. First, *Taf7l* is reduced in abundance of the mRNA in the B6-ChrX^MSM^ and B6-ChrXT^MSM^ testes to approximately one-third of that in B6 testes. *Taf7l* encodes a male germ cell-specific paralogue of the transcription factor IID (TFIID) subunit TAF7. TFIID is a highly conserved general transcription factor that is required for transcription of protein-coding genes by RNA polymerase II. *Taf7l* knockout male mice exhibit age-dependent spermatogenic defects, a decreased testis weight, and significantly low production of sperm [46]. Biochemical studies have demonstrated that TAF7L is closely associated with the TFIID subunit TBP in meiotic and postmeiotic male germ cells [47]. Thus, a reduction of *Taf7l* mRNA may affect the transcriptional activity of genes related to meiotic and postmeiotic processes in germ cells. Two other genes were nuclear mRNA export factors, *Nxf2* and *Nxf3*, which are specifically expressed in germ cells and Sertoli cells in the testis, respectively [48], [49]. Our data showed that the gene expression level of *Nxf2* and *Nxf3* in B6-ChrX^MSM^ and B6-ChrXT^MSM^ testes was decreased to one-third and one-tenth of that in B6 testes, respectively. *Nxf2*-deficient male mice exhibit meiotic arrest and are sterile [48]. NXF family members are known to play roles in not only nuclear mRNA export but also various aspects of post-transcriptional mRNA metabolism [50]. It is notable that the above three genes, which were significantly downregulated in B6-ChrX^MSM^ and B6-ChrXT^MSM^ testes and restored the expression in B6-Chr1^MSM/B6^XT^MSM^ testes, were categorized as the second type in terms of expression levels relative to the MSM strain. Coevolution between *cis*-regulatory elements and *trans*-acting factors is more frequently observed in sex-specific genes than in other genes [51], which might accelerate the transcriptional divergence of these genes.

The differential gene expression was observed genome-widely at 7 dpp in the testes of B6-ChrX^MSM^ and B6-ChrXT^MSM^ strains. At this stage, 70% of the differentially expressed genes were common to B6-ChrX^MSM^ and B6-ChrXT^MSM^ testes, and 90% of the common genes were located on autosomes. These data suggest that the significantly reduced expression of these predominant genes located on the distal half of the X chromosome affect global gene expression at the later stages (7 dpp onward) in B6-ChrX^MSM^ and B6-ChrXT^MSM^ testes. GO analysis revealed that many of the genome-wide differentially expressed genes are involved in meiosis, including stimulated by retinoic acid gene 8 (*Stra8*) on chromosome 6 and cellular retinoic acid binding protein I (*Crabp1*) on chromosome 9. The abundance of these transcripts was significantly reduced in B6-ChrX^MSM^ and B6-ChrXT^MSM^ testes. Retinoic acid (RA) is known to be essential for germ cells to enter meiosis in both the ovary and testis. RA stimulates *Stra8* expression to induce expression of downstream genes required for transition into meiosis [52], [53]. CRABP1 is a cellular RA-binding protein that is expressed in spermatogonia [54]. The reduced expression of these master regulatory genes required for meiotic entry is a likely cause of the significant reduction of meiotic spermatocytes in prepubertal B6-ChrX^MSM^ and B6-ChrXT^MSM^ testes. We also found other genes downregulated in the X chromosomal substitution strains, which are known to be involved in meiosis. These genes included three synaptonemal complex proteins genes (*Sycp1*, *Sycp2*, and *Sycp3*), DMC1 dosage suppressor of mck1 homolog (*Dmc1*) that functions in meiosis-specific homologous recombination, testis-expressed genes (*Tex11* and *Tex15*), maelstrom homolog (*Mael*), and structural maintenance of chromosomes 1B (*Smc1b*). The reduced expression of these genes might explain the defect of synapsis observed in early spermatocytes of B6-ChrX^MSM^ and B6-ChrXT^MSM^ testes [13]. Thus, the misregulated gene expression in premeiotic spermatogonia may influence the cellular processes in germ cells at meiotic and possibly postmeiotic stages.

In B6-Chr1^MSM/B6^XT^MSM^ testes, the introduction of MSM-derived chromosome 1 restored the reproductive phenotypes and transcriptional misregulation. At 5 dpp, the misregulation was not restored in B6-Chr1^MSM/B6^XT^MSM^ testes, and the delayed restoration began at 7 dpp, which is reminiscent of the fact that emergence of meiotic spermatocytes was delayed in B6-Chr1^MSM/B6^XT^MSM^ testes. However not all X-linked transcripts were restored in B6-Chr1^MSM/B6^XT^MSM^ testes. These findings imply that genetic factor(s) on chromosome 1 are not sufficient for complete restoration of B6-ChrXT^MSM^ phenotypes. Consistent with this notion, the reduced testis weight in B6-ChrXT^MSM^ strain was restored to some extent by the introduction of other MSM-derived autosomes. Thus, an intricate genetic mechanism is involved in the reproductive isolation between mouse subspecies.

In summary, our study provides comprehensive characterization of the transcriptional profile in X chromosome substitution strains, demonstrating that transcriptional regulation divergence between the two mouse subspecies contributes to the improper gene expression in the genetic background of different subspecies. Evolutionary divergence in transcriptional regulation explains the phenotypic differences between subspecies and occasionally causes incompatibilities attributed to diseases and reproductive disorders that contributes to reproductive isolation. Our study has revealed an insight into gene expression divergence in mammals and the occurrence of speciation.

## Materials and Methods

### Mouse Strains

The MSM strain was established and maintained at the National Institute of Genetics (NIG), Mishima, Japan. B6 mice were purchased from CLEA Japan (Tokyo, Japan), and maintained at NIG. The full set of chromosome substitution strains was established using MSM as the chromosome donor and B6 as the host (background) strain [43], and were maintained at NIG. Almost the entire length (5.3–166.3 Mb) and distal region (86.5–163.3 Mb) of the X chromosome were substituted by the counterparts of the MSM strain in B6-ChrX^MSM^ and B6-XT^MSM^ strains, respectively (Figure S15) [27], [43]. In the B6-Chr1^MSM^ strain, almost the entire length (3.2–193.4 Mb) of chromosome 1 was substituted by the counterpart of the MSM strain (Figure S15) [43]. All animal experiments were approved by the Animal Care and Use Committee of NIG.

### RNA Preparation and Microarray Experiments

After euthanasia of prepubertal males, their testes were dissected, immersed in RNAlater (Ambion, Austin, TX), and stored at −20°C. Total RNA from the whole testis was extracted using QIAzol Lysis Reagent and an RNeasy Mini kit (Qiagen. Valencia, CA). DNase digestion of the purified RNA with RNase-free DNase (Qiagen) was performed according to the manufacturer's protocol. RNA quality was checked with an Agilent Bioanalyzer 2100 (Agilent Technologies, Santa Clara, CA), and only RNA samples with >9 RNA integrity number were used in experiments. Biotinylated amplified RNA was generated from 100 ng total RNA using an Affymetrix GeneChip 3′IVT Express Kit, and then hybridized to an Affymetrix Mouse Genome 430 2.0 array (Affymetrix, Santa Clara, CA) following the manufacturer's instructions. For each strain or genotype, three distinct mice were tested separately. For the B6-Chr1^MSM/B6^XT^MSM^ strain, a total of eight mice were tested, because they showed variable expression profiles.

### Microarray Analysis

Microarray data were analyzed with GeneSpring GX software (Agilent Technologies). The expression data were normalized using the RMA method. Because genotypes at boundary regions of each chromosome substitution strain are not defined precisely, probe sets in these regions were excluded from the expression analysis (Figure S15). We also excluded probe sets with an unknown chromosome location. Chromosomal locations for the mouse reference genome (mm9) were obtained from the Ensembl genome browser (http://asia.ensembl.org/index.html). Y-linked probe sets were omitted because their number was extremely small. As mentioned in the Results, polymorphic probe sets were filtered out. Only probe sets with a raw signal intensity of >10 in at least one of the paired strains were used for the analysis. GO analysis and calculation of *P*-value were performed by GeneSpring GX software. The Ansari-Bradley test and the Fisher's z-transformation were conducted by the R package. The expression data was deposited in the NCBI Gene Expression Omnibus (GSE50687).

### Evaluation of Male Reproductive Phenotypes

The testes of 3- to 4-month-old euthanized males were dissected and weighed. For histological analysis, the testes were placed in fresh Bouin's fixative at room temperature. Excess fixative was removed with 70% ethanol. The tissues were then dehydrated and embedded in paraffin for microtome sectioning. The sections (6 µm) were stained with hematoxylin and eosin. To count meiotic spermatocytes, we used 18-day-old males. Immunocytochemistry was performed as described previously [13] using an antibody against SYCP3 (Novus Biologicals, Littleton, CO). After immunocytochemistry, the slides were counterstained with Hoechst 33258. The proportion of meiotic spermatocytes was measured by the frequency of SYCP3-positive spermatocytes among all testicular cells.

### QTL Analysis

A total of 314 male progeny from crosses between B6-ChrXT^MSM^ females and (B6-Chr1^MSM^×B6) F1 males were used to map quantitative trait loci for testis weight and relative testis weight per body weight (RTW). All males were genotyped at 80 single nucleotide polymorphism (SNP) markers on chromosome 1 by using the Sequenom MassARRAY iPLEX Gold Assay (Sequenom) as previously described [36]. Genotype and trait data from all male progeny were indicated in Table S12. Single marker analysis and interval mapping (walk speed: 1 Mb) were performed by using QTL Cartographer (http://statgen.ncsu.edu/qtlcart/).

## Supporting Information

Figure S1 Reproductive phenotypes of B6-ChrX^MSM^ and B6-ChrXT^MSM^ testes. (A) Histology of the testes at developmental stages during the first cycle of spermatogenesis. Meiotic spermatocytes emerged in the seminiferous tubules of the B6 testis at 10 dpp, whereas they were rarely observed in B6-ChrX^MSM^ and B6-ChrXT^MSM^ testes. A decrease in the number of spermatocytes became obvious in B6-ChrX^MSM^ and B6-ChrXT^MSM^ testes at subsequent stages after 14 dpp. Scale bar: 250 µm (B) Frequency of primary spermatocytes among all testicular cells in each strain at 18 dpp. Both B6-ChrX^MSM^ and B6-ChrXT^MSM^ testes showed a significant decrease in the frequency of meiotic spermatocytes. *P<0.01, two-tailed Student's *t*-test. (C) Comparison of body and paired testis weights. B6-ChrX^MSM^ and B6-ChrXT^MSM^ testes showed a significant decrease in testis weight. (D) Result from interval mapping for testis weight at adulthood. The significant threshold level is shown as a dotted line.
(PDF)

Figure S2 Criteria for polymorphic probe sets used in the microarray analysis. Polymorphism scores of the Affymetrix Mouse 430 2.0 probe sets are indicated in upper table. Polymorphism scores were defined by the number of probes identified in the MSM genome and the number of polymorphic probes per probe set. In this study, probe sets with polymorphism scores of ≤10 were used in the analysis as conserved probe sets (black square). The numbers of probe sets for each polymorphism score are indicated in lower table.
(PDF)

Figure S3 Gene expression in B6-ChrX^MSM^ testes at 5 dpp. Fold changes of gene expression in B6-ChrX^MSM^ relative to that in B6 is indicated in a log_2_ scale. Transcripts in red show significantly different expression by the Benjamini-Hochberg FDR corrected moderate *t*-test (*P*<0.05; fold change ≥1.50).
(PDF)

Figure S4 Gene expression in B6-ChrX^MSM^ testes at 7 dpp. Fold changes of gene expression in B6-ChrX^MSM^ relative to that in B6 is indicated in a log_2_ scale. Transcripts in red show significantly different expression by the Benjamini-Hochberg FDR corrected moderate *t*-test (*P*<0.05; fold change ≥1.50).
(PDF)

Figure S5 Gene expression in B6-ChrXT^MSM^ testes at 5 dpp. Fold changes of gene expression in B6-ChrXT^MSM^ relative to that in B6 is indicated in a log_2_ scale. Transcripts in red show significantly different expression by the Benjamini-Hochberg FDR corrected moderate *t*-test (*P*<0.05; fold change ≥1.50).
(PDF)

Figure S6 Gene expression in the testes of X chromosome congenic mouse at 5 dpp. Vertical dotted line indicates the boundary of recombination. The distal region from the boundary was derived from MSM. Fold changes of gene expression in the X-chromosomal congenic strain relative to that in B6 is indicated in a log_2_ scale. Transcripts in red show significantly different expression by the Benjamini-Hochberg FDR corrected moderate *t*-test (*P*<0.05; fold change ≥1.50).
(PDF)

Figure S7 Gene expression in B6-ChrXT^MSM^ testes at 7 dpp. Fold changes of gene expression in B6-ChrXT^MSM^ relative to that in B6 is indicated in a log_2_ scale. Transcripts in red show significantly different expression by the Benjamini-Hochberg FDR corrected moderate *t*-test (*P*<0.05; fold change ≥1.50).
(PDF)

Figure S8 Testis weight of F1 hybrid males from crosses between B6-ChrXT^MSM^ females and males of a cohort of chromosome substitution strains. Weights of paired testes (upper) and relative testis weight normalized to body weight (lower) are indicated. Parental strains are represented by the black bar and F1 male progeny are represented by the gray bar. Numbers in parenthesis are the number of tested samples. *Bonferroni-corrected *P*<0.05, two-tailed Student's *t*-test.
(PDF)

Figure S9 Gene expression in B6-Chr1^MSM/B6^XT^MSM^ testes at 5 dpp. Fold changes of gene expression in B6-Chr1^MSM/B6^XT^MSM^ relative to that in B6 is indicated in a log_2_ scale. Transcripts in red show significantly different expression by the Benjamini-Hochberg FDR corrected moderate *t*-test (*P*<0.05; fold change ≥1.50).
(PDF)

Figure S10 Gene expression in restored B6-Chr1^MSM/B6^XT^MSM^ testes at 7 dpp. Fold changes of gene expression in B6-Chr1^MSM/B6^XT^MSM^ relative to that in B6 is indicated in a log_2_ scale. Transcripts in red show significantly different expression by the Benjamini-Hochberg FDR corrected moderate *t*-test (*P*<0.05; fold change ≥1.50).
(PDF)

Figure S11 Gene expression in non-restored B6-Chr1^MSM/B6^XT^MSM^ testes at 7 dpp. Fold changes of gene expression in B6-Chr1^MSM/B6^XT^MSM^ relative to that in B6 is indicated in a log_2_ scale. Transcripts in red show significantly different expression by the Benjamini-Hochberg FDR corrected moderate *t*-test (*P*<0.05; fold change ≥1.50).
(PDF)

Figure S12 Validation by RT-PCR of eight downregulated X-linked genes. Expression values of B6-ChrXT^MSM^, B6-Chr1^MSM/B6^XT^MSM^, and MSM testes relative to those in B6 controls are shown. Left graphs (black) and right graphs (gray) are results from RT-PCR and microarray experiments, respectively. Values of gene expression in RT-PCR analysis were normalized to *β-actin* gene expression. XT and F1 indicate B6-ChrXT^MSM^ and B6-Chr1^MSM/B6^XT^MSM^, respectively. Significant restoration of values in B6-Chr1^MSM/B6^XT^MSM^ testes relative to those in B6-ChrXT^MSM^ testes is indicated by single and double asterisks (two-tailed Student's *t*-test, *P<0.05; **P<0.01). Primers for RT-PCR are indicated above graphs for each gene. Primer for *β*-actin; F, 5′-ATG ACG ATA TCG CTG CGC TGG T-3′; R, 5′-ATA GGA GTC CTT CTG ACC CAT TCC-3′; Cano DA *et al*, Development 2004;131(14):3457-67.
(PDF)

Figure S13 Genetic analysis to detect responsible QTLs on chromosome 1 for the restoration of testis weight. (A) Mating scheme used in the QTL analysis. (B, C) Results of the QTL analysis of chromosome 1. Single marker analysis (B) and interval mapping (C) were performed using 80 SNP markers on chromosome 1 and 314 male progeny. Two traits, testis weight and ratio of testis weight to body weight (RTW), were used.
(PDF)

Figure S14 Venn diagram of misregulated SSC-enriched and non-SSC-enriched gene transcripts in B6-ChrX^MSM^ (upper) and B6-ChrXT^MSM^ (lower). SSC-enriched genes show the expression levels higher than 3-fold in non-SSC cells, and non-SSC enriched genes show the expression levels higher than 3-fold in SSCs in a report by Yang *et al*. Note that all SSC-enriched misregulated genes were downregulated in B6-ChrX^MSM^ and B6-ChrXT^MSM^.
(PDF)

Figure S15 X chromosomal subregion substituted by the MSM-derived counterpart for each strain. The subregions of B6 and MSM genomes are indicated in gray and orange, respectively. Numbers indicate the positions of genetic markers for genotyping at the boundaries. Because the genotypes in stripe subregions are undefined, probe sets in these regions were excluded from the analysis.
(PDF)

Table S1 Polymorphic probe sets on chromosome 1 and X.
(XLSX)

Table S2 Ansari-Bradley test for grouped B6-ChrX^MSM^ samples by signal intensity.
(XLSX)

Table S3 Differentially expressed transcripts in B6-ChrX^MSM^ testes as compared to B6 testes at 5 dpp.
(XLSX)

Table S4 Differentially expressed transcripts in B6-ChrX^MSM^ testes as compared to B6 testes at 7 dpp.
(XLSX)

Table S5 Differentially expressed transcripts in B6-ChrXT^MSM^ testes as compared to B6 testes at 5 dpp.
(XLSX)

Table S6 Differentially expressed transcripts in B6-ChrXT^MSM^ testes as compared to B6 testes at 7 dpp.
(XLSX)

Table S7 Differentially expressed probe sets shared by B6-ChrX^MSM^ and B6-ChrXT^MSM^ samples by Venn diagram.
(XLSX)

Table S8 Differentially expressed probes in B6-Chr1^MSM/B6^XT^MSM^ testes as compared to B6 testes at 5 dpp.
(XLSX)

Table S9 Differentially expressed probes in restored B6-Chr1^MSM/B6^XT^MSM^ testes as compared to B6 testes at 7 dpp.
(XLSX)

Table S10 List of X-linked transcripts expressing in 7 dpp B6-ChrX^MSM^ and transcriptional regulation relative to B6 or MSM.
(XLSX)

Table S11 List of distal-X-linked transcripts expressing in 7 dpp B6-ChrXT^MSM^ and transcriptional regulation relative to B6 or MSM.
(XLSX)

Table S12 Trait and genotype data of male progeny used in QTL analysis.
(XLSX)
