# Supplementary figures and images for: Evolutionarily Diverged Regulation of X-chromosomal Genes as a Primal Event in Mouse Reproductive Isolation
Source: PLoS Genet. 2014 Apr 17;10(4):e1004301. doi: 10.1371/journal.pgen.1004301 (PMC3990516; doi:10.1371/journal.pgen.1004301)

Figure S1

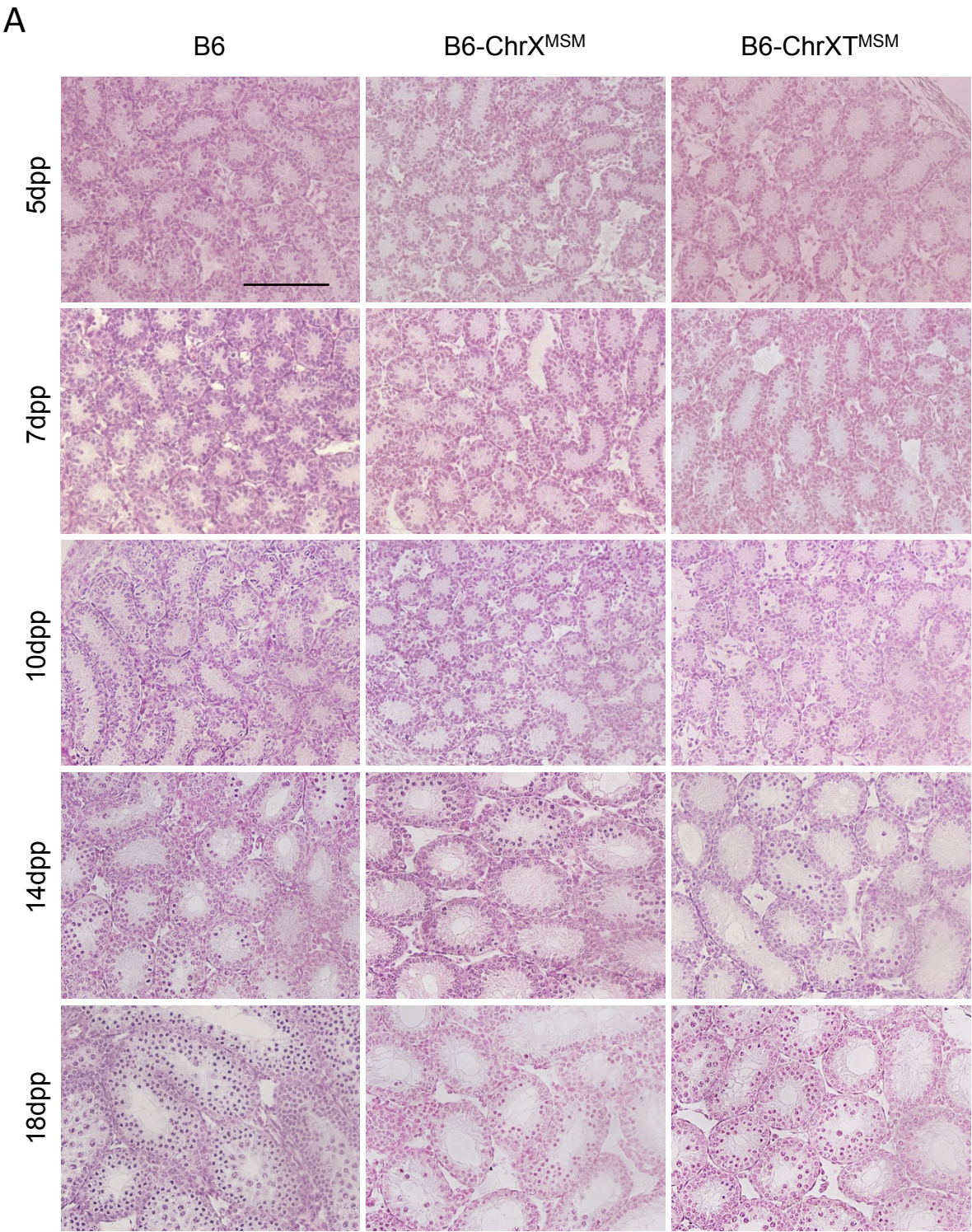

B

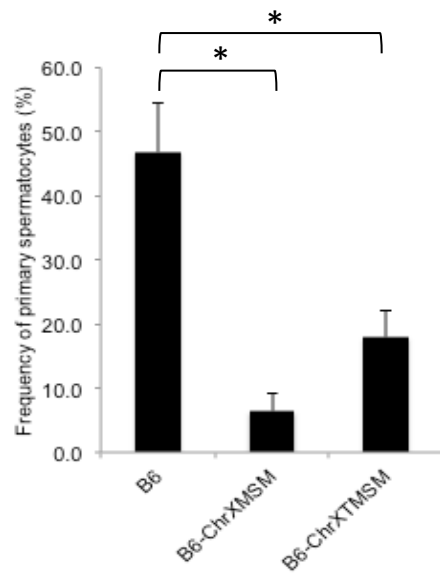

C

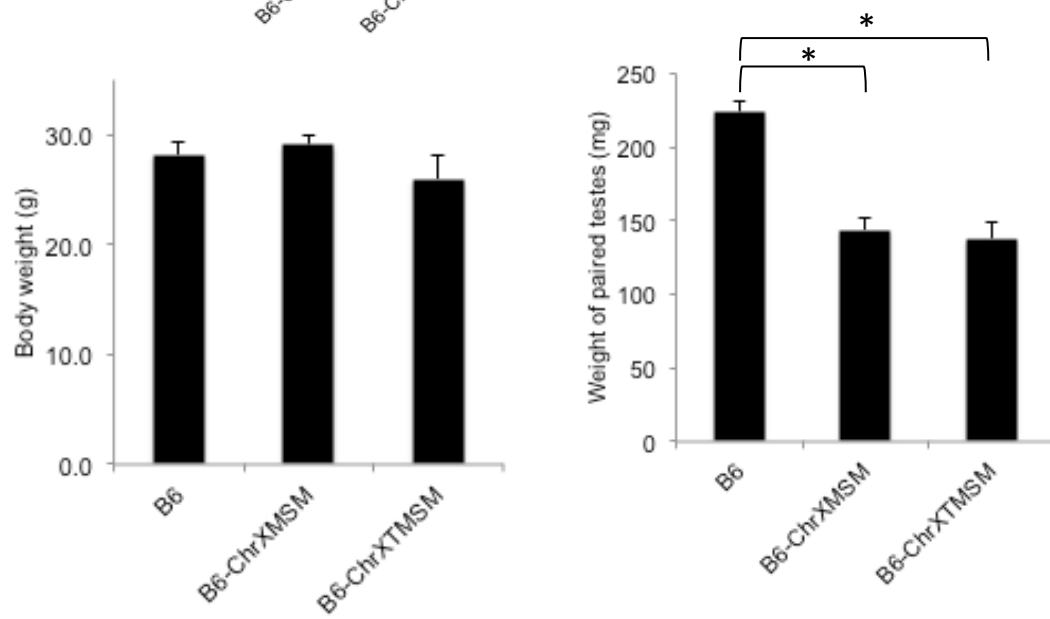

D

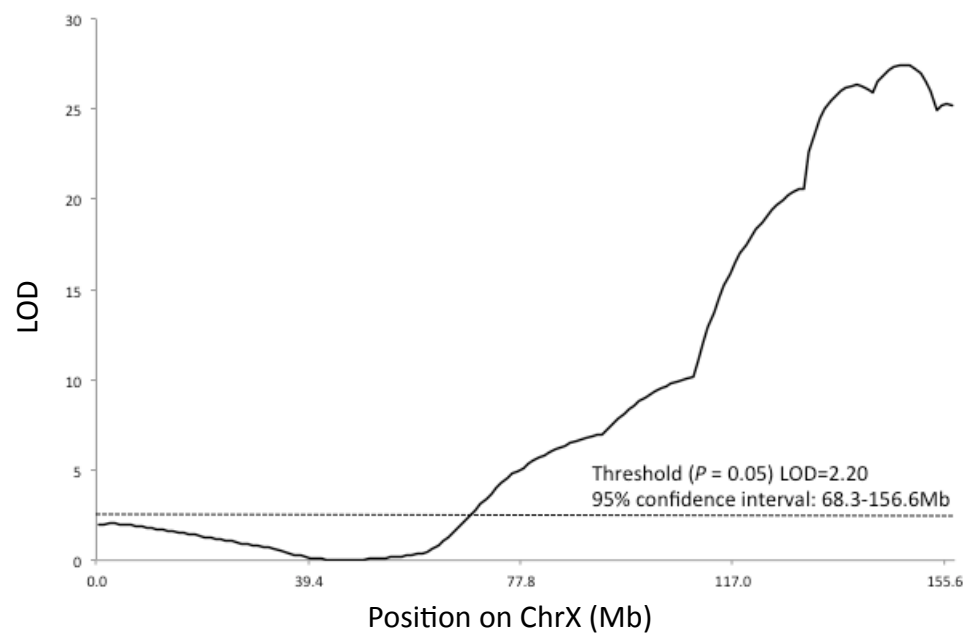

Supplement: Figure S1 — Reproductive phenotypes of B6-ChrXMSM and B6-ChrXTMSM testes. (A) Histology of the testes at developmental stages during the first cycle of spermatogenesis. Meiotic spermatocytes emerged in the seminiferous tubules of the B6 testis at 10 dpp, whereas they were rarely observed in B6-ChrXMSM and B6-ChrXTMSM testes. A decrease in the number of spermatocytes became obvious in B6-ChrXMSM and B6-ChrXTMSM testes at subsequent stages after 14 dpp. Scale bar: 250 µm (B) Frequency of primary spermatocytes among all testicular cells in each strain at 18 dpp. Both B6-ChrXMSM and B6-ChrXTMSM testes showed a significant decrease in the frequency of meiotic spermatocytes. *P<0.01, two-tailed Student's t-test. (C) Comparison of body and paired testis weights. B6-ChrXMSM and B6-ChrXTMSM testes showed a significant decrease in testis weight. (D) Result from interval mapping for testis weight at adulthood. The significant threshold level is shown as a dotted line. (PDF) [file pgen.1004301.s001.pdf]

Figure S3

5 dpp B6-ChrX<sup>MSM</sup>

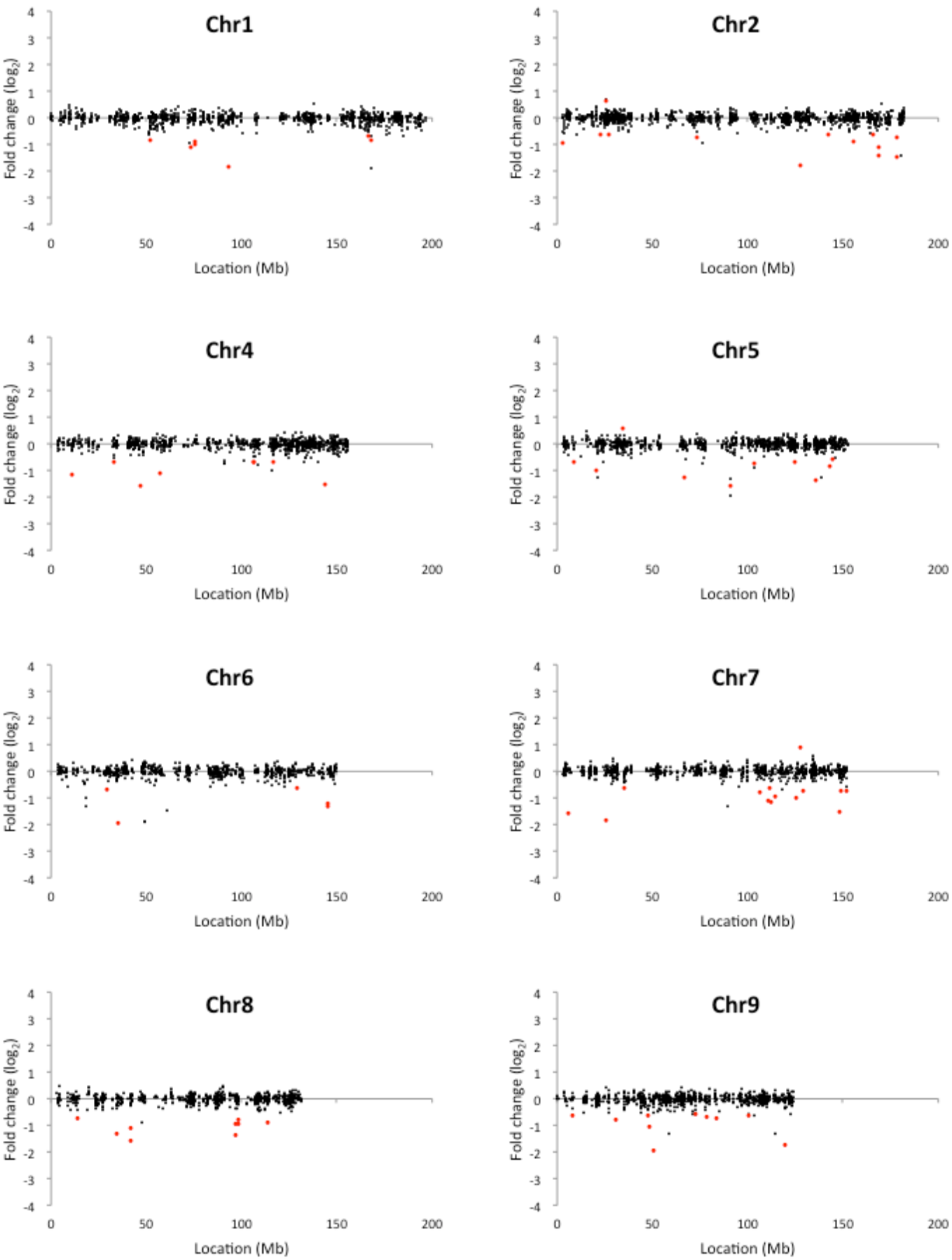

5 dpp B6-ChrX<sup>MSM</sup>

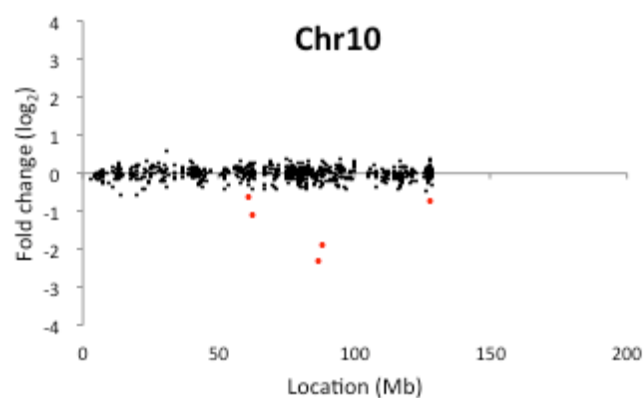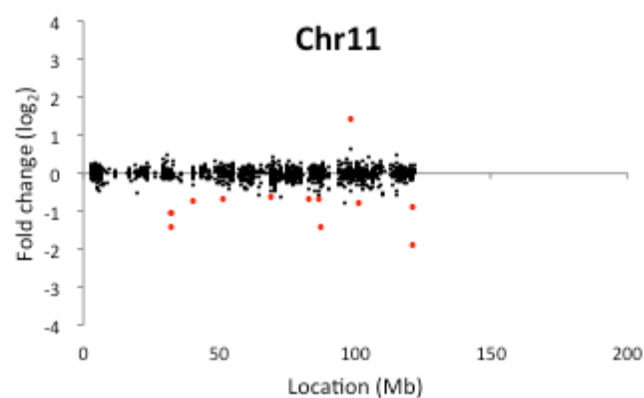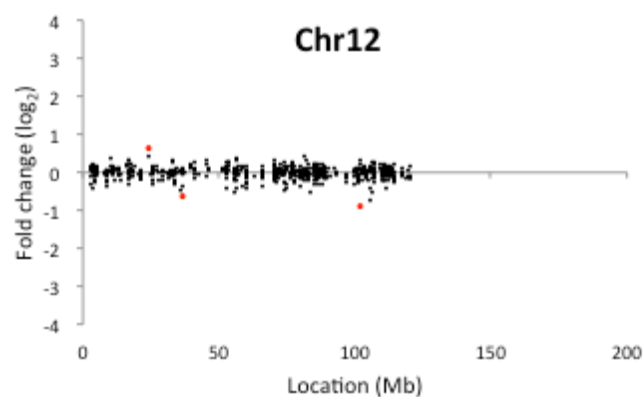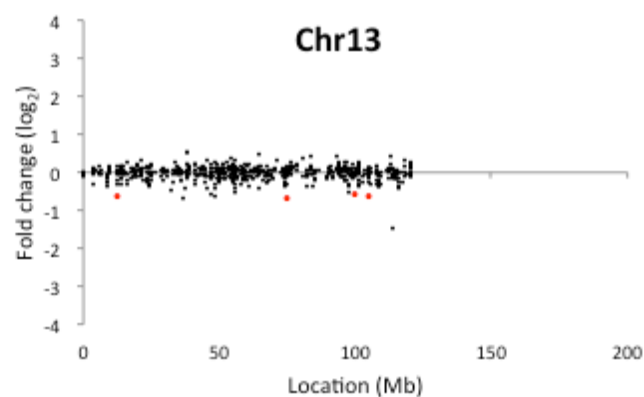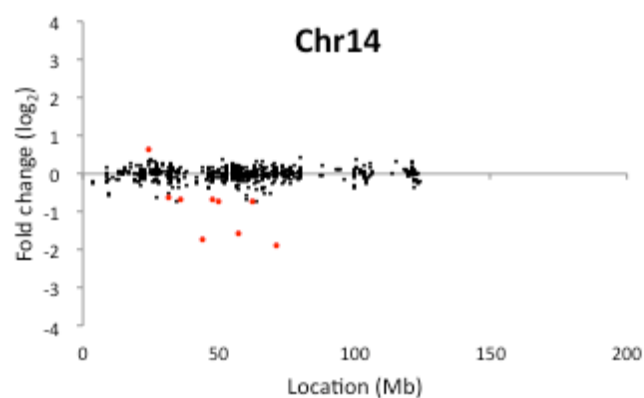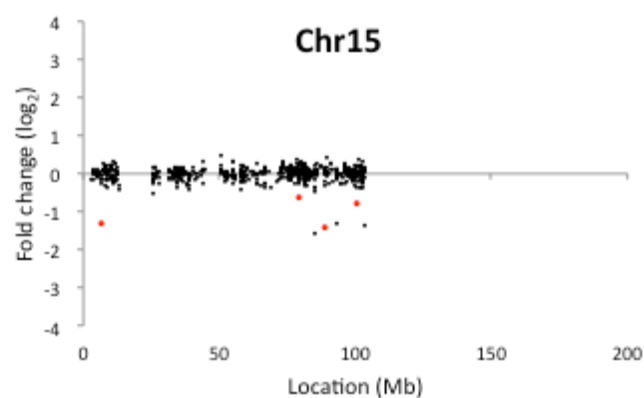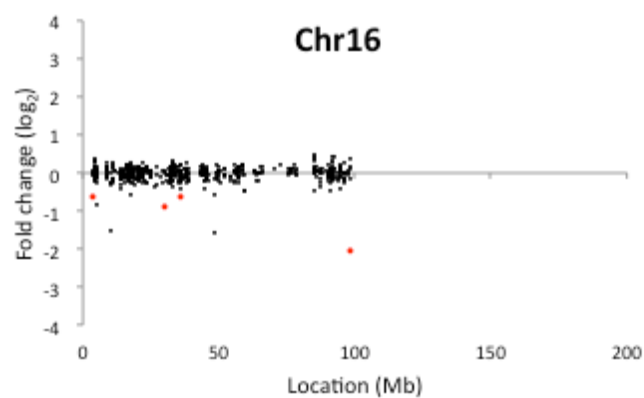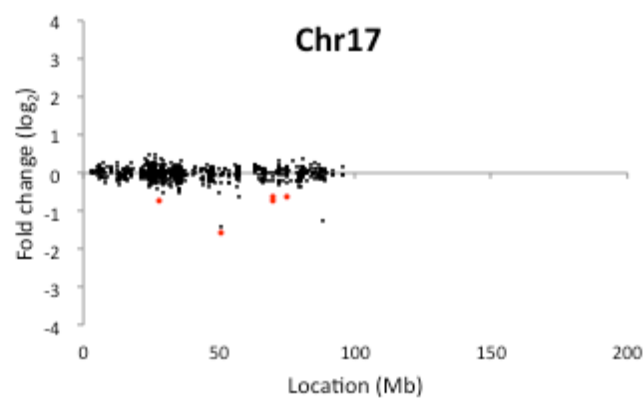

5 dpp B6-ChrX<sup>MSM</sup>

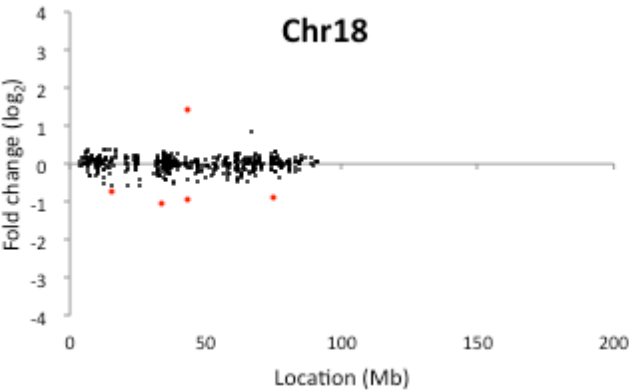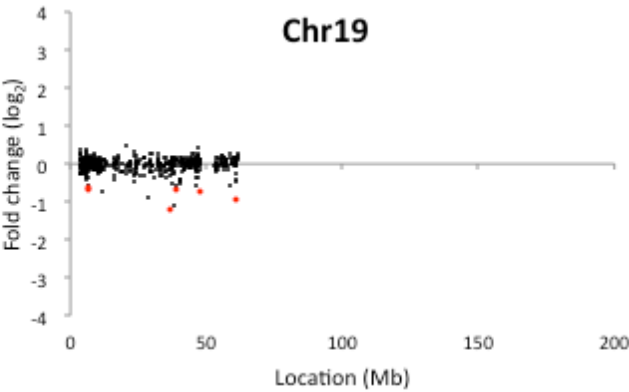

Supplement: Figure S3 — Gene expression in B6-ChrXMSM testes at 5 dpp. Fold changes of gene expression in B6-ChrXMSM relative to that in B6 is indicated in a log2 scale. Transcripts in red show significantly different expression by the Benjamini-Hochberg FDR corrected moderate t-test (P<0.05; fold change ≥1.50). (PDF) [file pgen.1004301.s003.pdf]

Figure S4

7 dpp B6-ChrX<sup>MSM</sup>

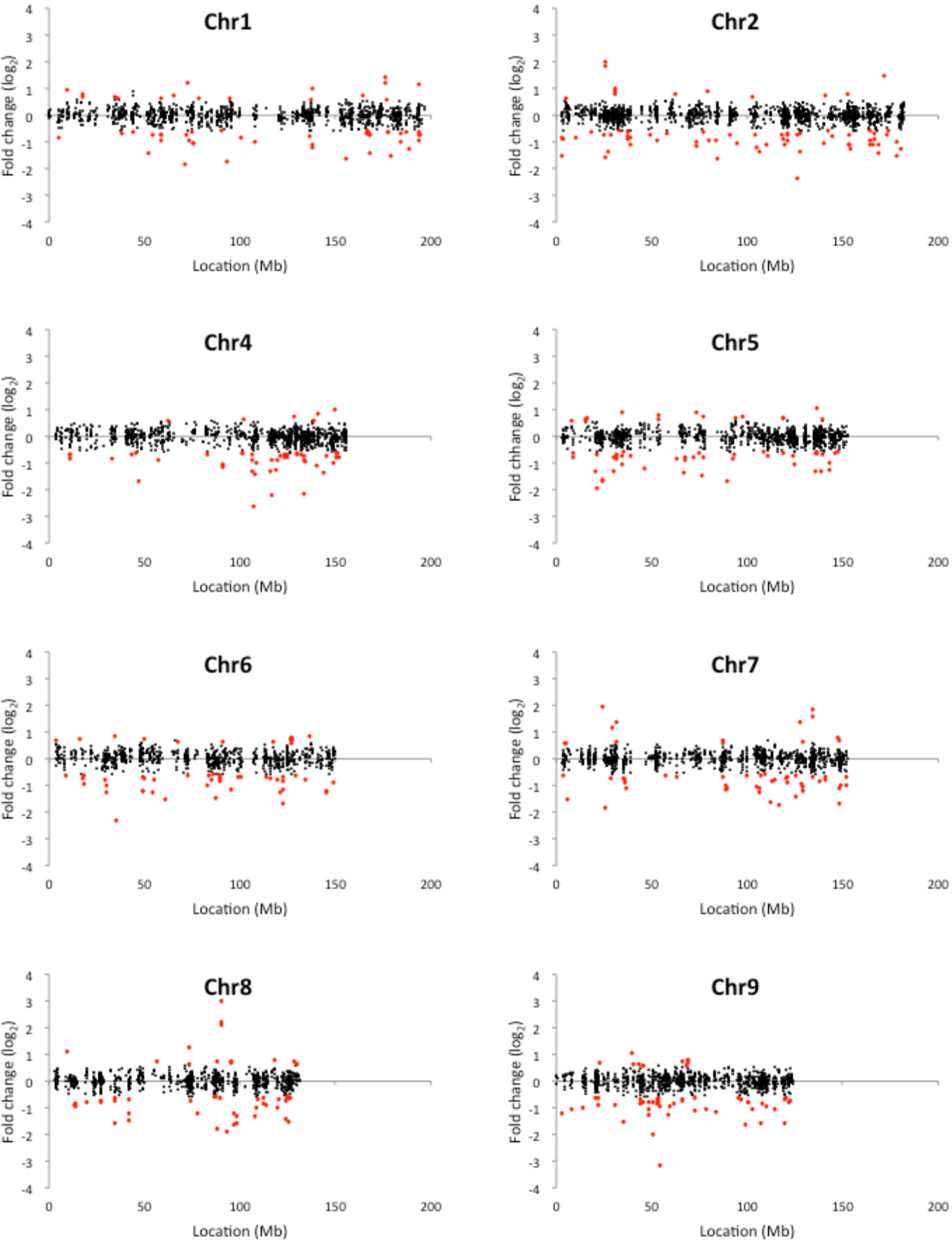

7 dpp B6-ChrX<sup>MSM</sup>

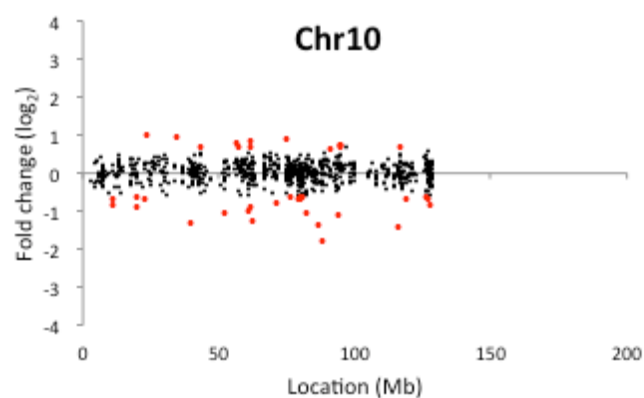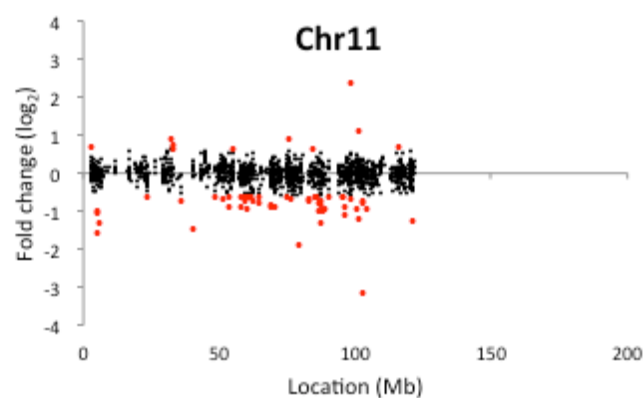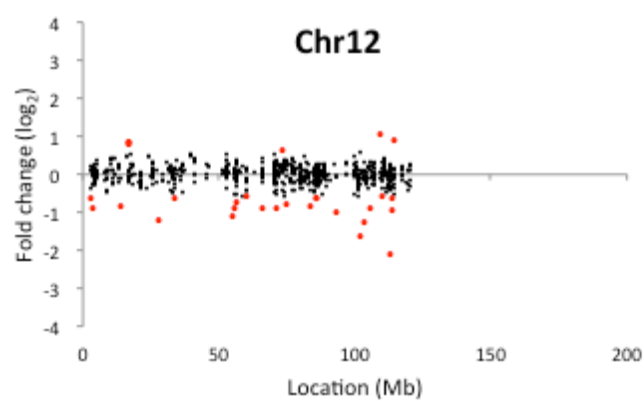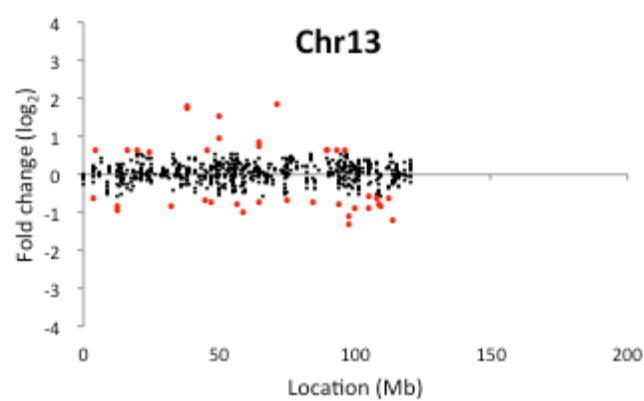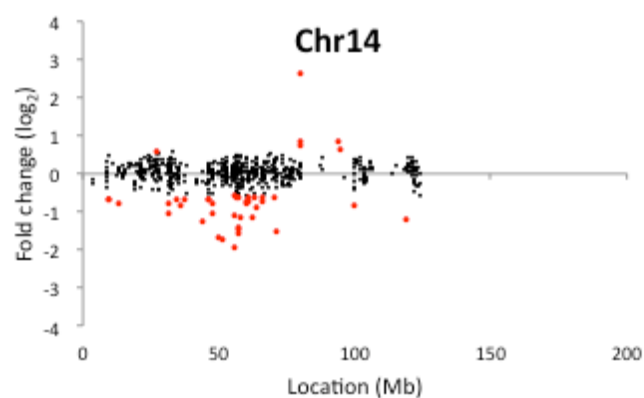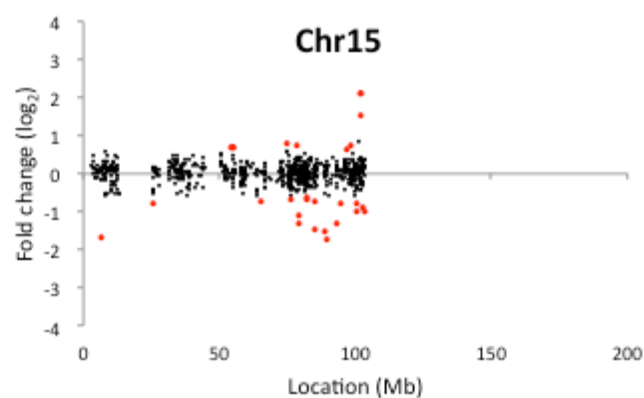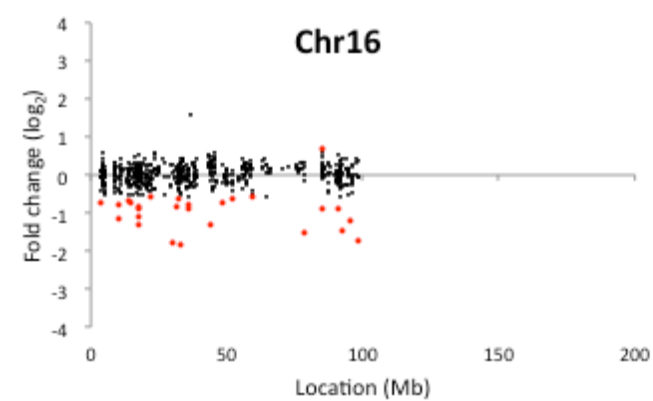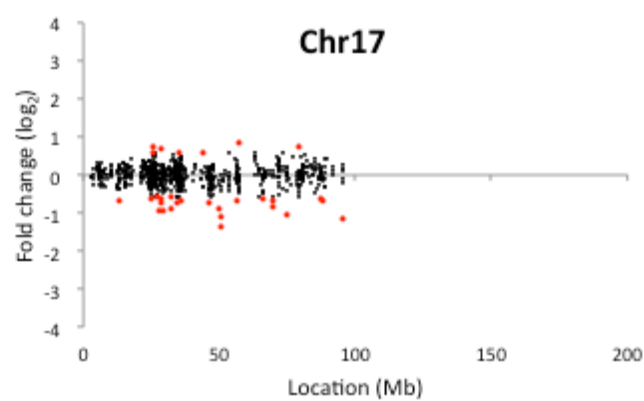

7 dpp B6-ChrX<sup>MSM</sup>

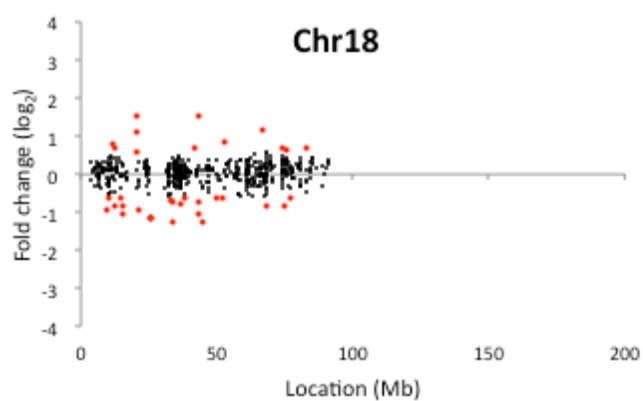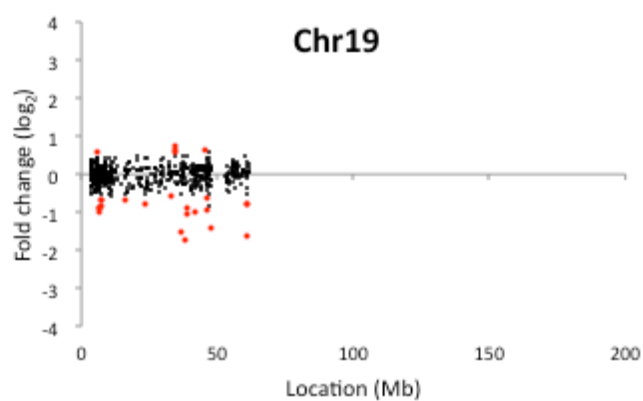

Supplement: Figure S4 — Gene expression in B6-ChrXMSM testes at 7 dpp. Fold changes of gene expression in B6-ChrXMSM relative to that in B6 is indicated in a log2 scale. Transcripts in red show significantly different expression by the Benjamini-Hochberg FDR corrected moderate t-test (P<0.05; fold change ≥1.50). (PDF) [file pgen.1004301.s004.pdf]

Figure S5

5 dpp B6-ChrXT<sup>MSM</sup>

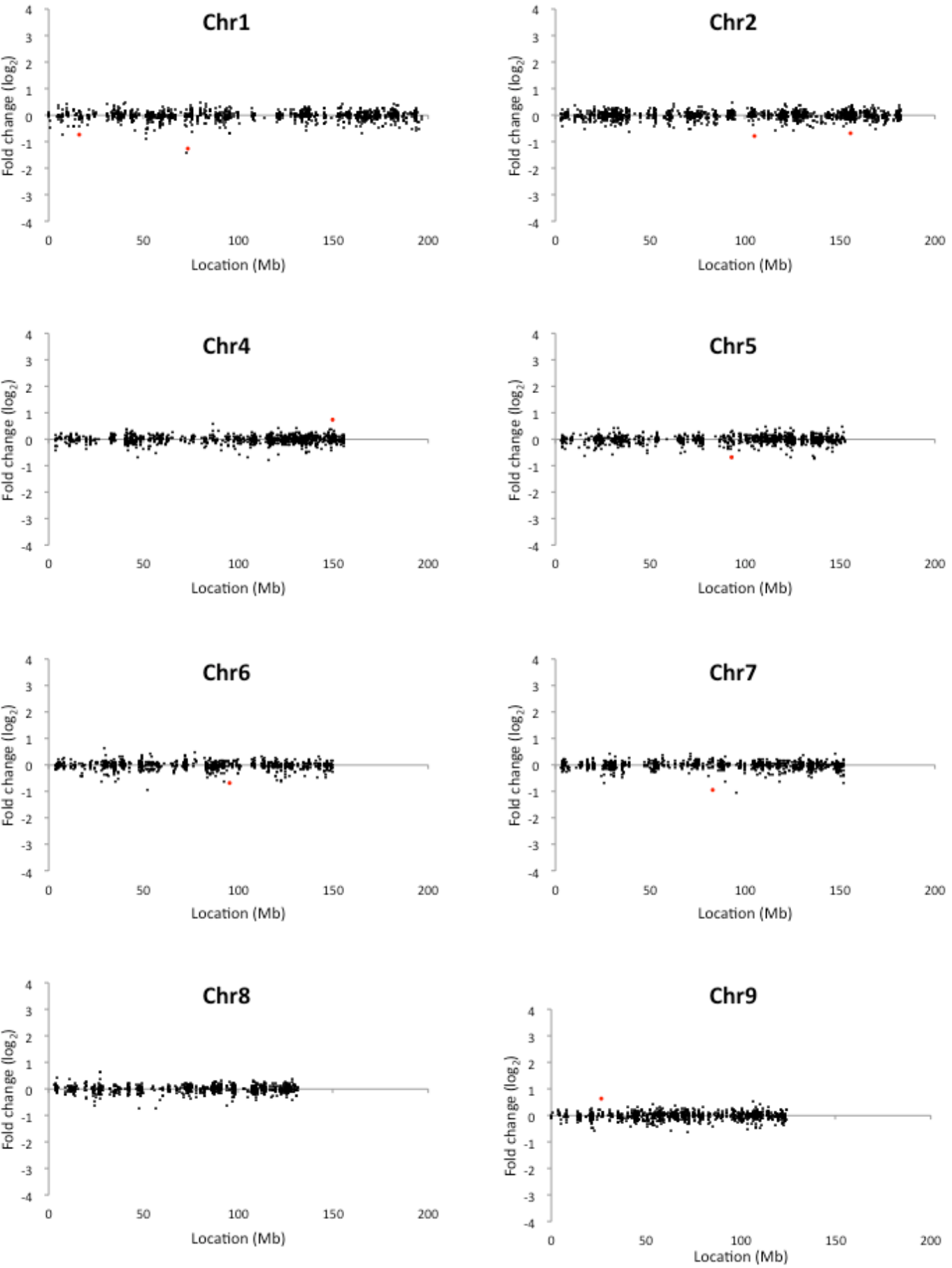

5 dpp B6-ChrXT<sup>MSM</sup>

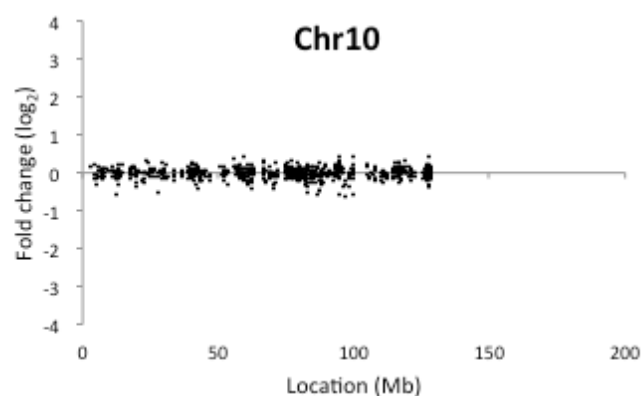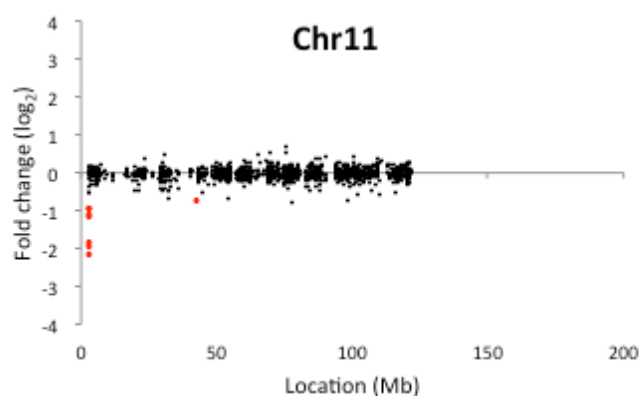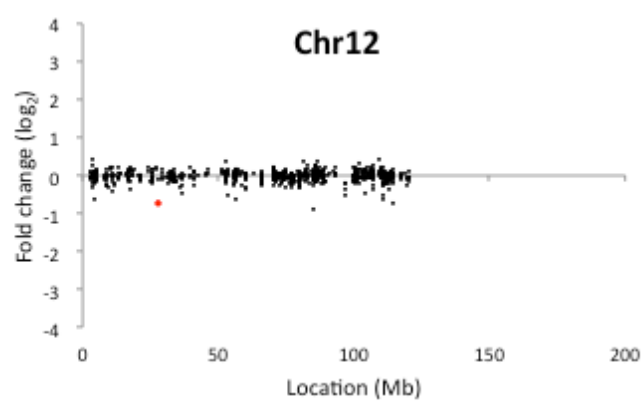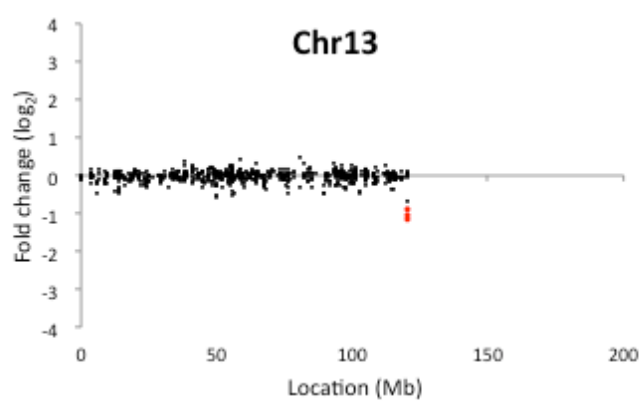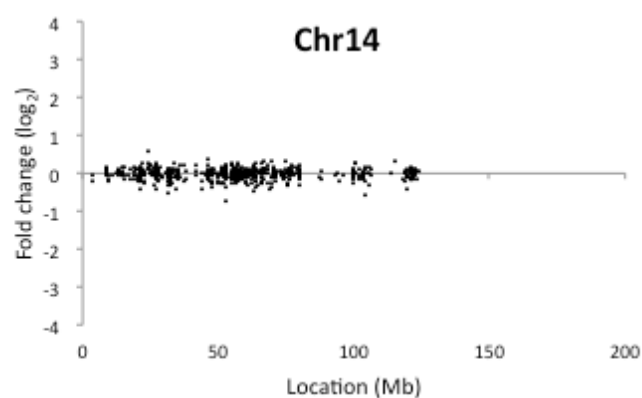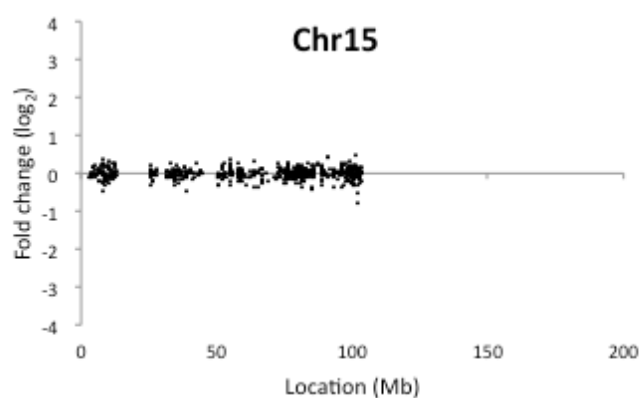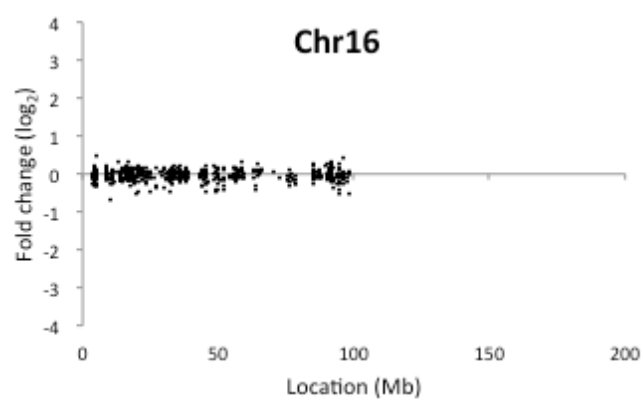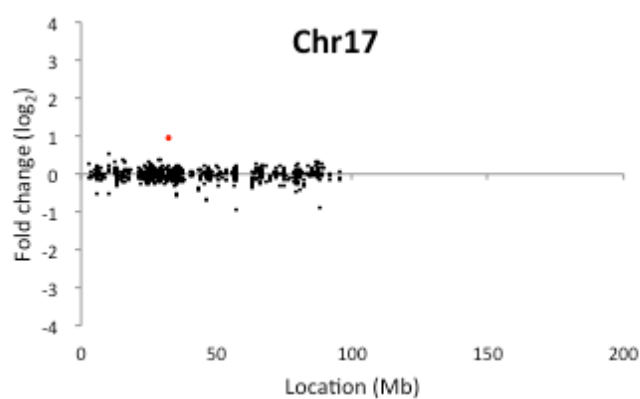

5 dpp B6-ChrXT<sup>MSM</sup>

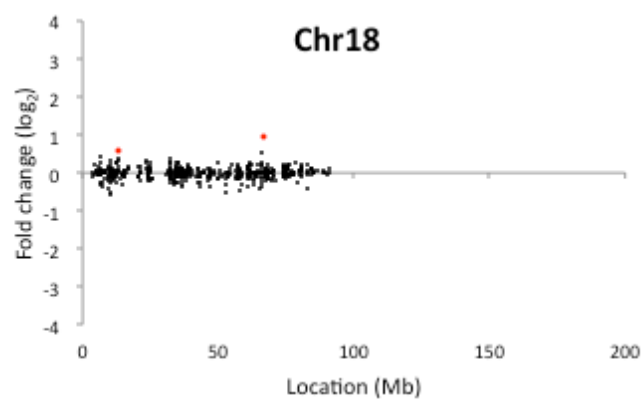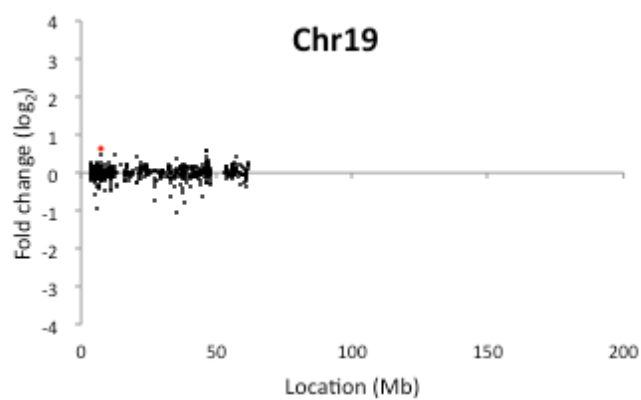

Supplement: Figure S5 — Gene expression in B6-ChrXTMSM testes at 5 dpp. Fold changes of gene expression in B6-ChrXTMSM relative to that in B6 is indicated in a log2 scale. Transcripts in red show significantly different expression by the Benjamini-Hochberg FDR corrected moderate t-test (P<0.05; fold change ≥1.50). (PDF) [file pgen.1004301.s005.pdf]

Figure S6

5 dpp congenic strain

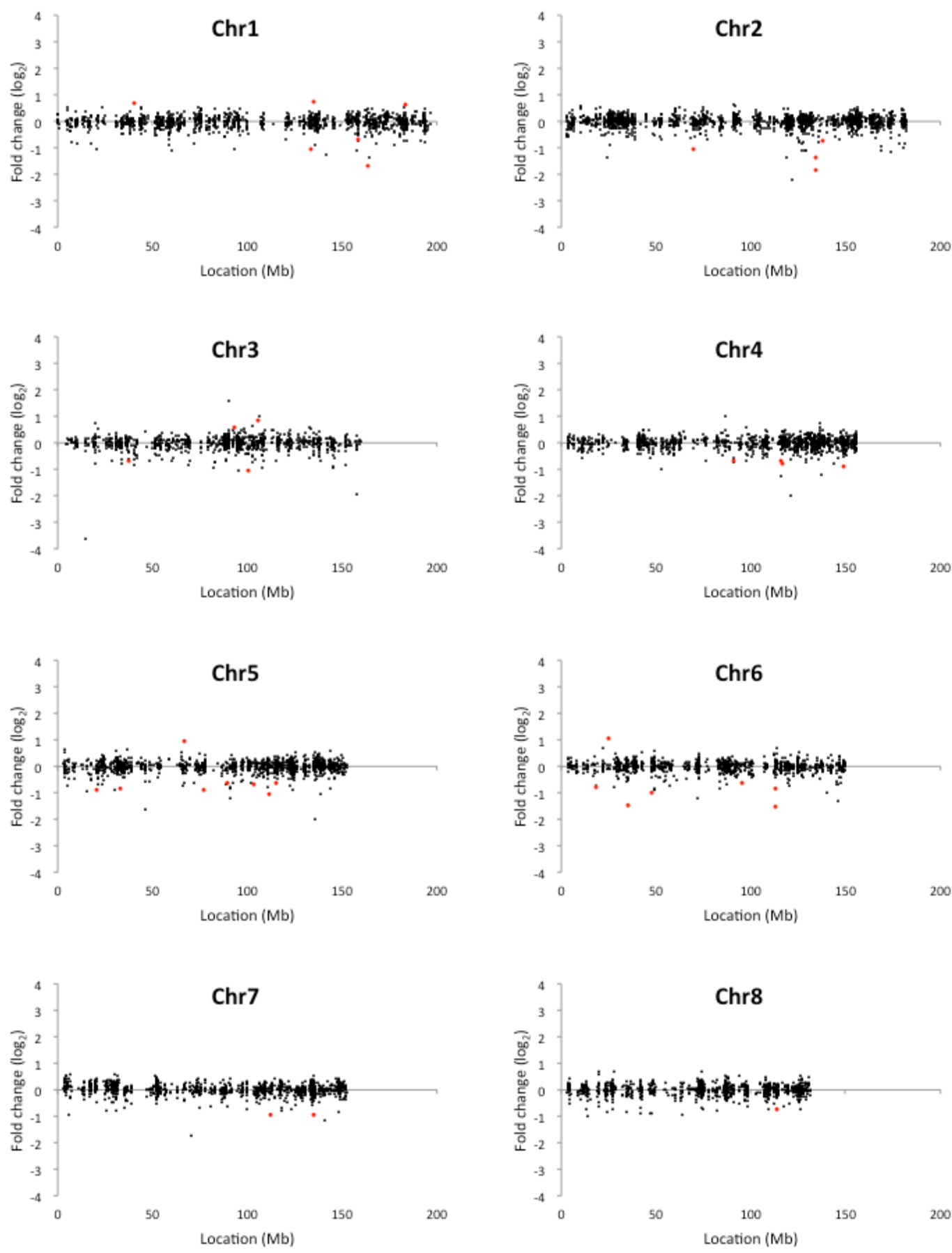

5 dpp B6-ChrXT<sup>MSM</sup>

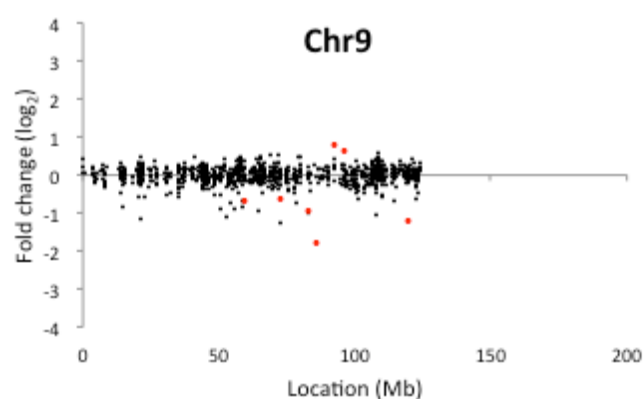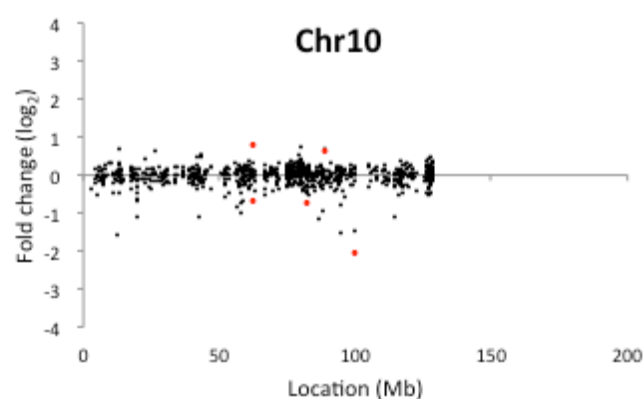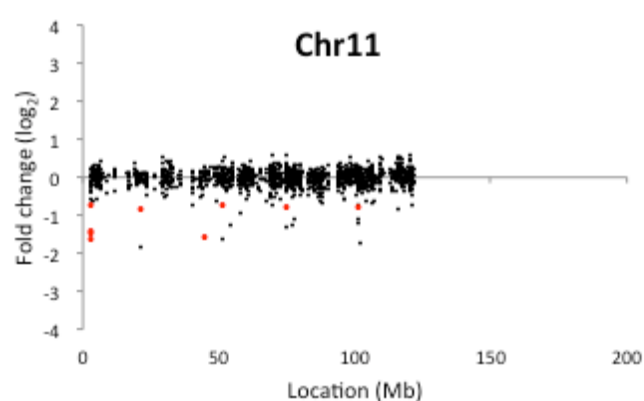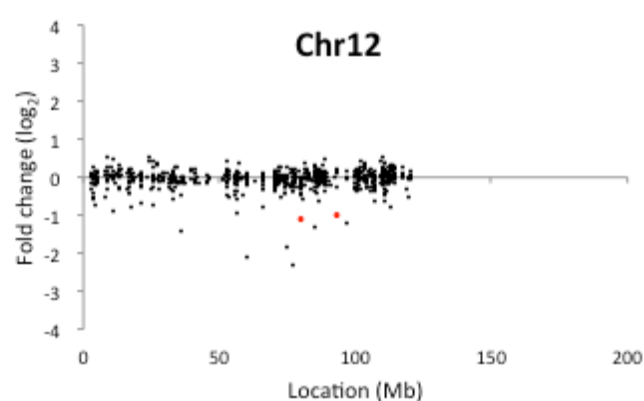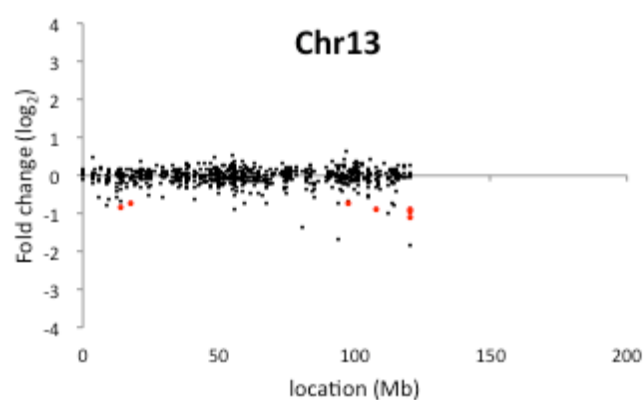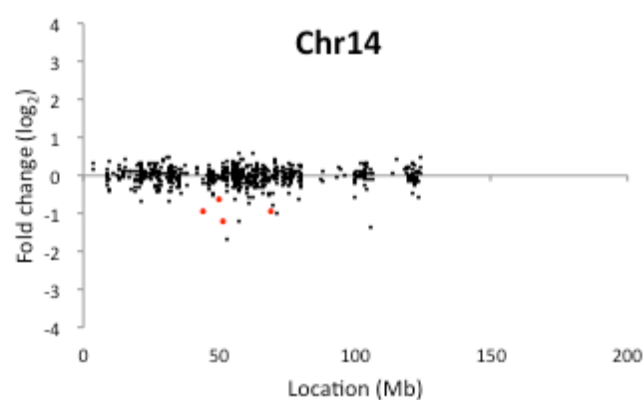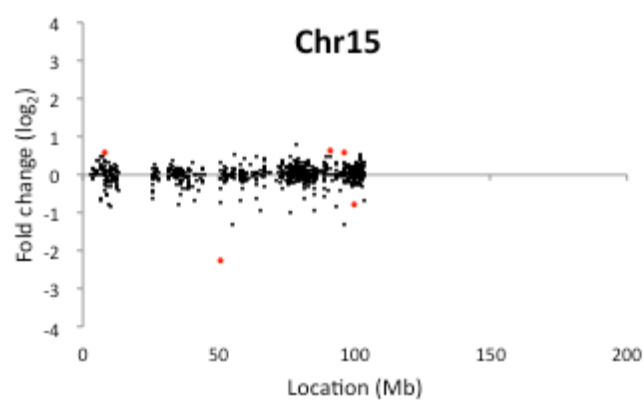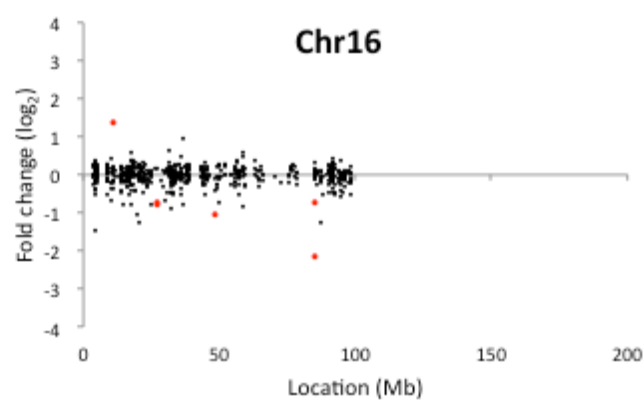

5 dpp B6-ChrXT<sup>MSM</sup>

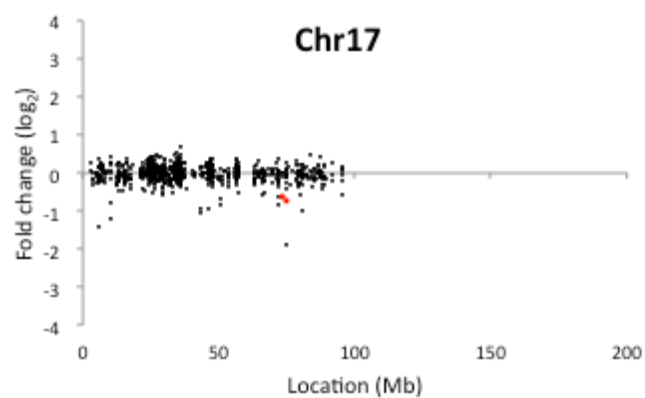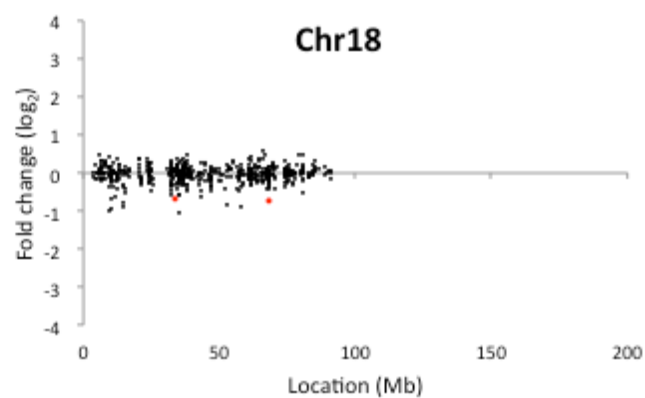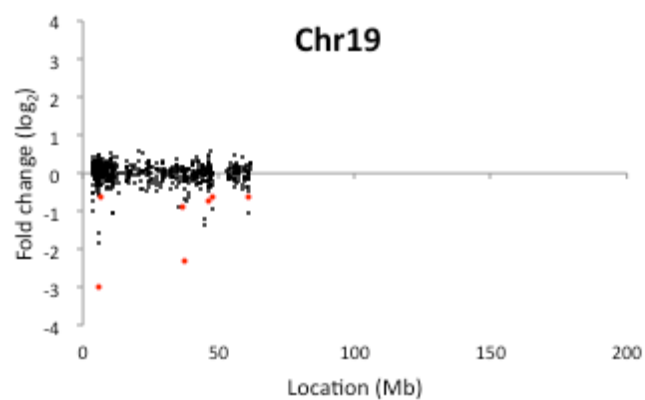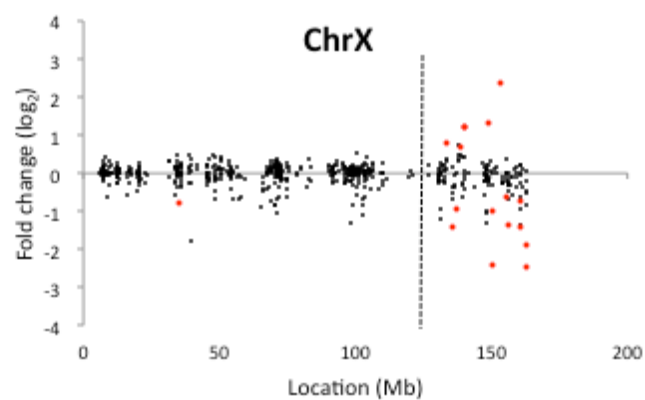

Supplement: Figure S6 — Gene expression in the testes of X chromosome congenic mouse at 5 dpp. Vertical dotted line indicates the boundary of recombination. The distal region from the boundary was derived from MSM. Fold changes of gene expression in the X-chromosomal congenic strain relative to that in B6 is indicated in a log2 scale. Transcripts in red show significantly different expression by the Benjamini-Hochberg FDR corrected moderate t-test (P<0.05; fold change ≥1.50). (PDF) [file pgen.1004301.s006.pdf]

Figure S7

7 dpp B6-ChrXT<sup>MSM</sup>

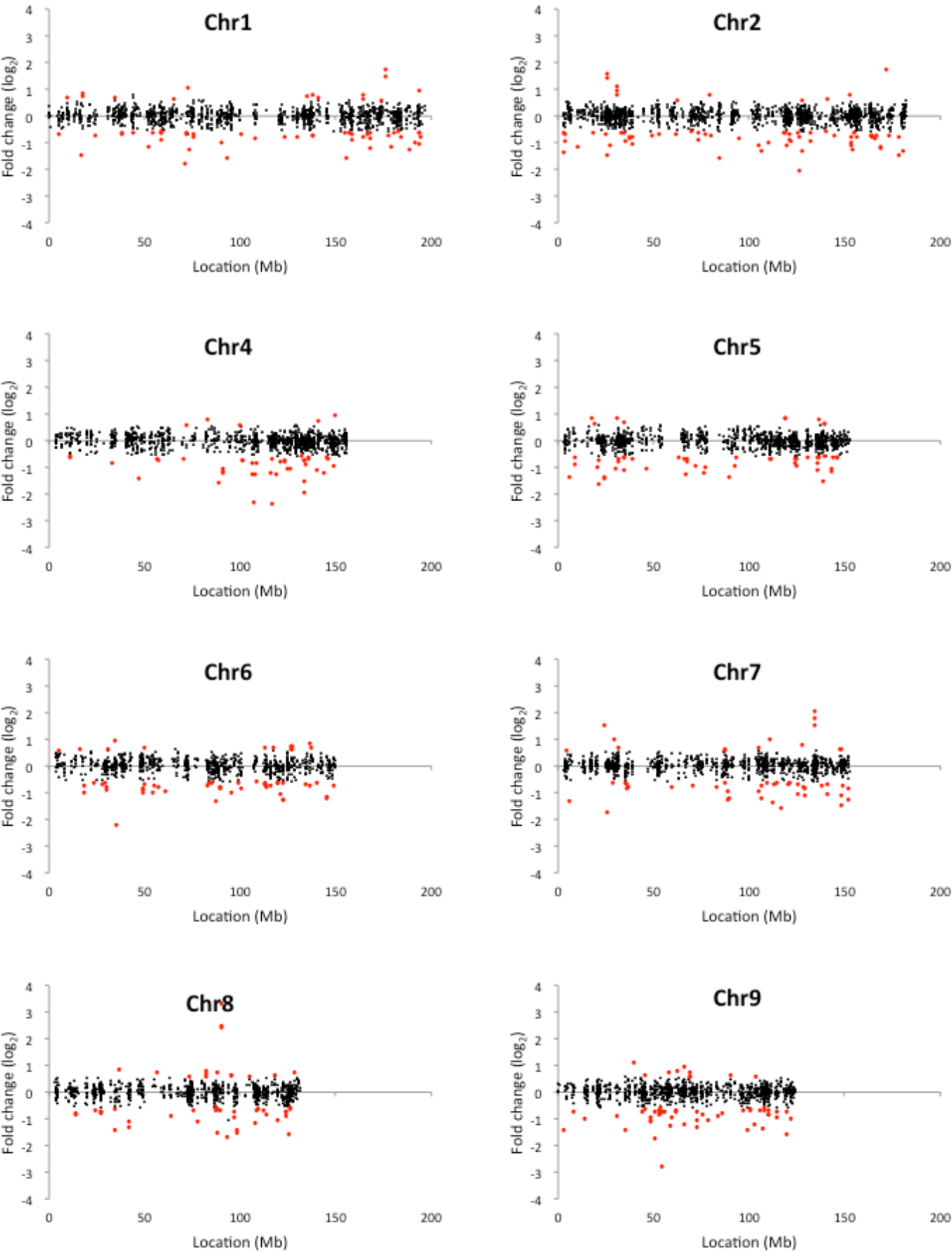

7 dpp B6-ChrXT<sup>MSM</sup>

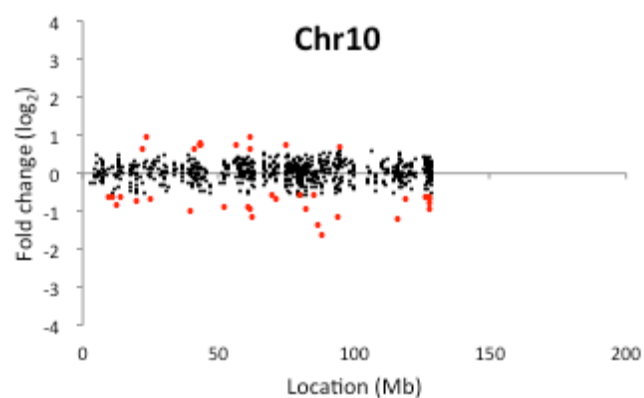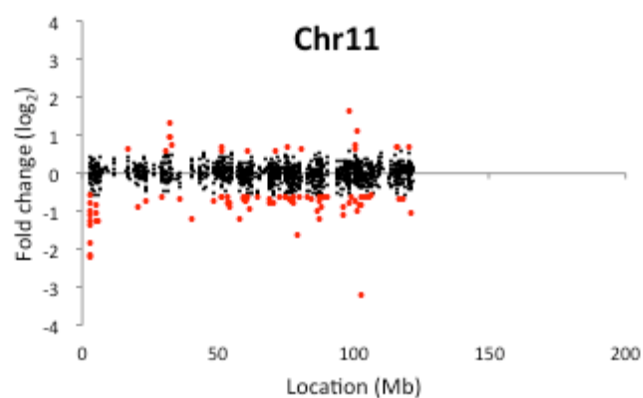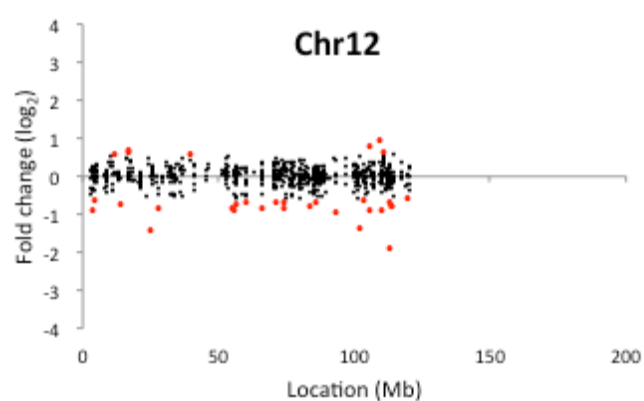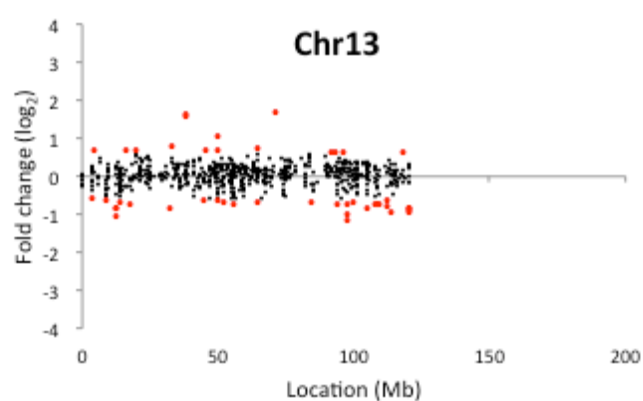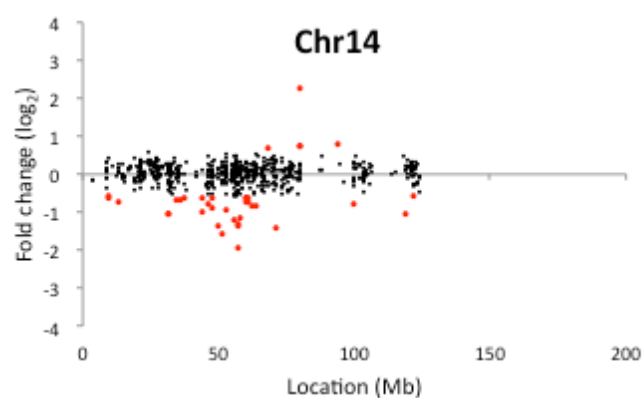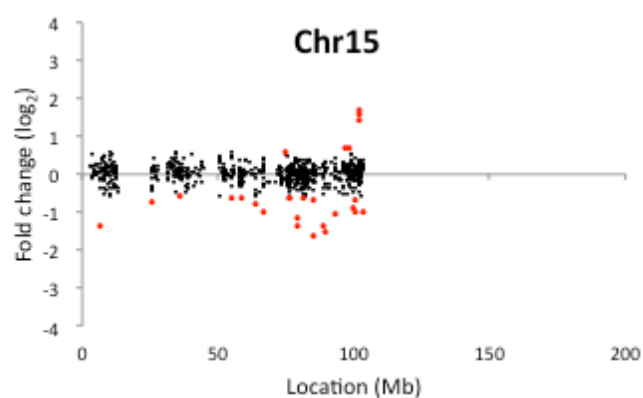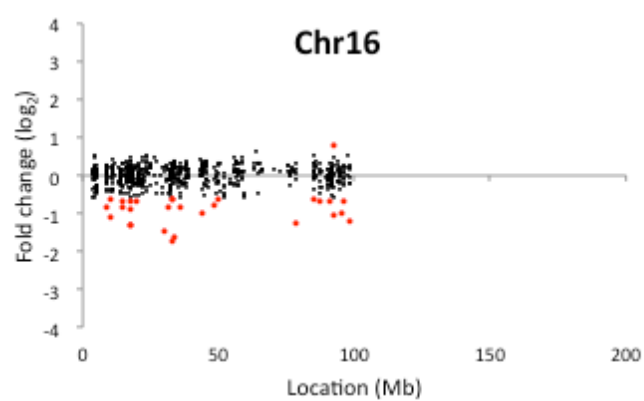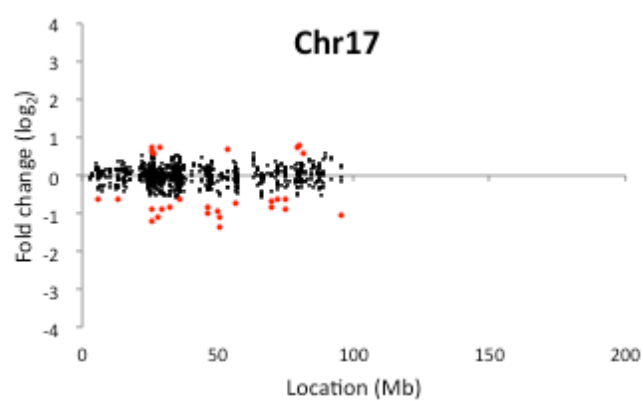

7 dpp B6-ChrXT<sup>MSM</sup>

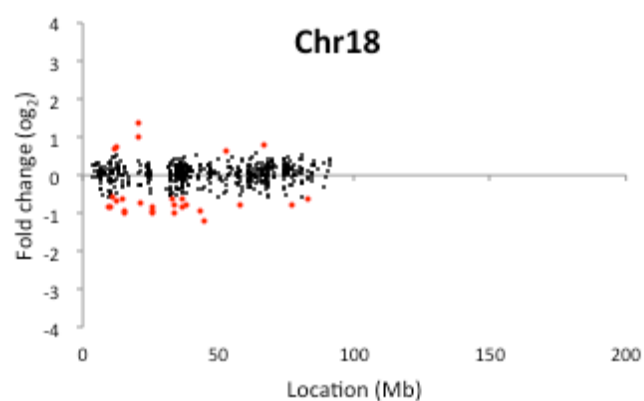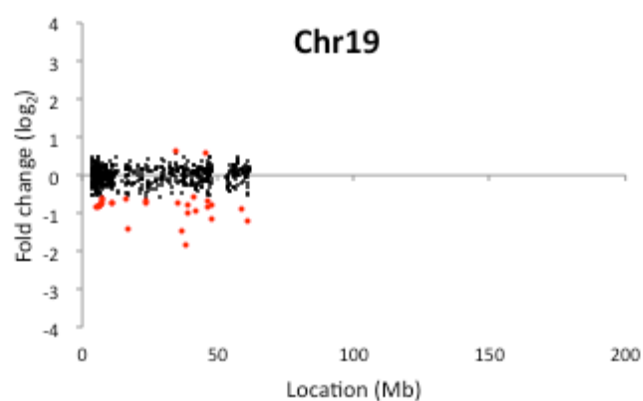

Supplement: Figure S7 — Gene expression in B6-ChrXTMSM testes at 7 dpp. Fold changes of gene expression in B6-ChrXTMSM relative to that in B6 is indicated in a log2 scale. Transcripts in red show significantly different expression by the Benjamini-Hochberg FDR corrected moderate t-test (P<0.05; fold change ≥1.50). (PDF) [file pgen.1004301.s007.pdf]

Figure S8

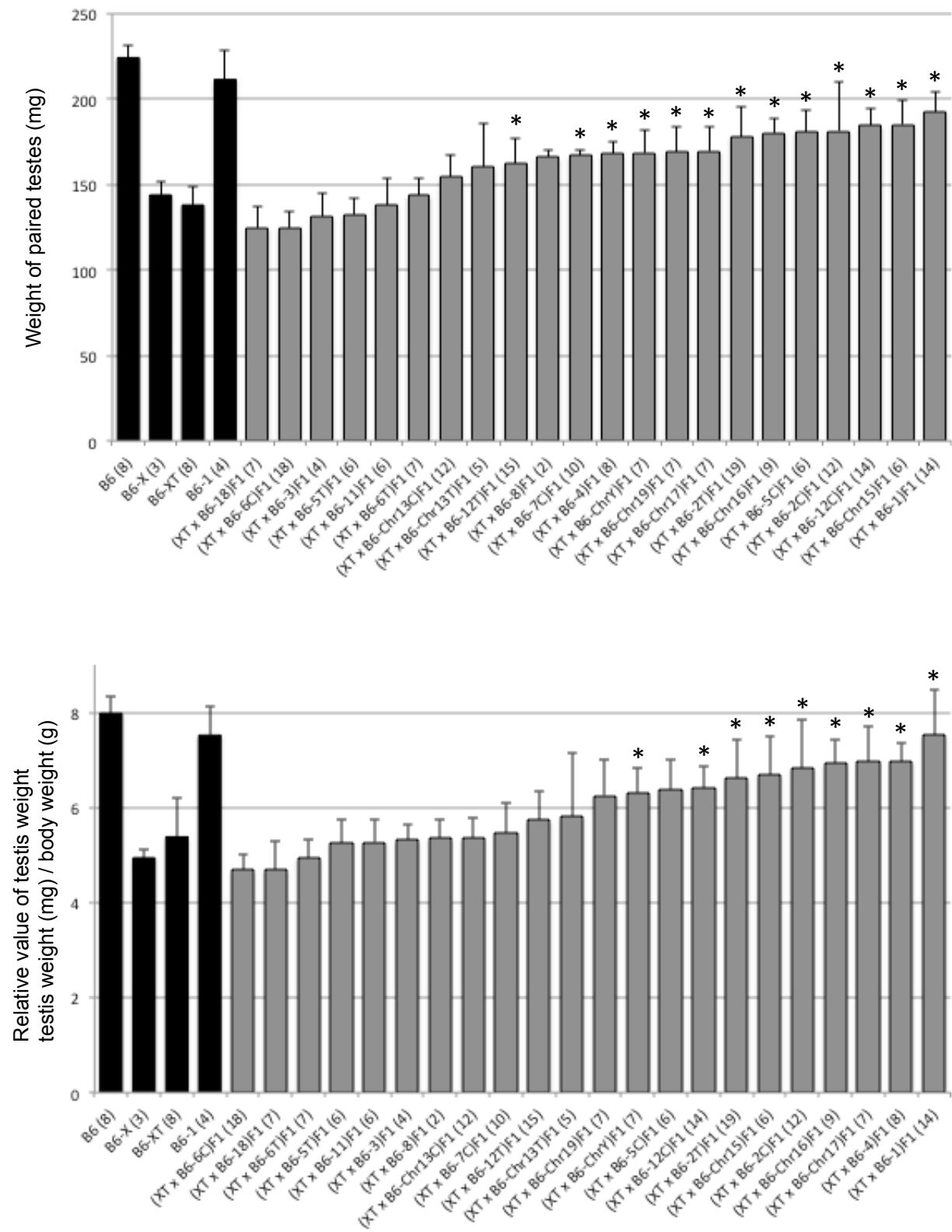

Supplement: Figure S8 — Testis weight of F1 hybrid males from crosses between B6-ChrXTMSM females and males of a cohort of chromosome substitution strains. Weights of paired testes (upper) and relative testis weight normalized to body weight (lower) are indicated. Parental strains are represented by the black bar and F1 male progeny are represented by the gray bar. Numbers in parenthesis are the number of tested samples. *Bonferroni-corrected P<0.05, two-tailed Student's t-test. (PDF) [file pgen.1004301.s008.pdf]

Figure S13

A

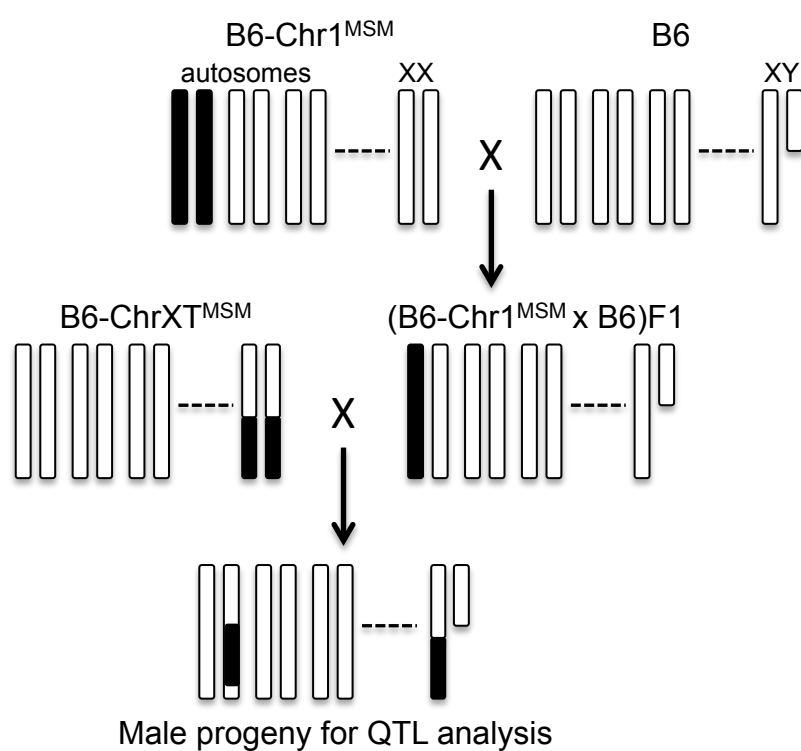

B

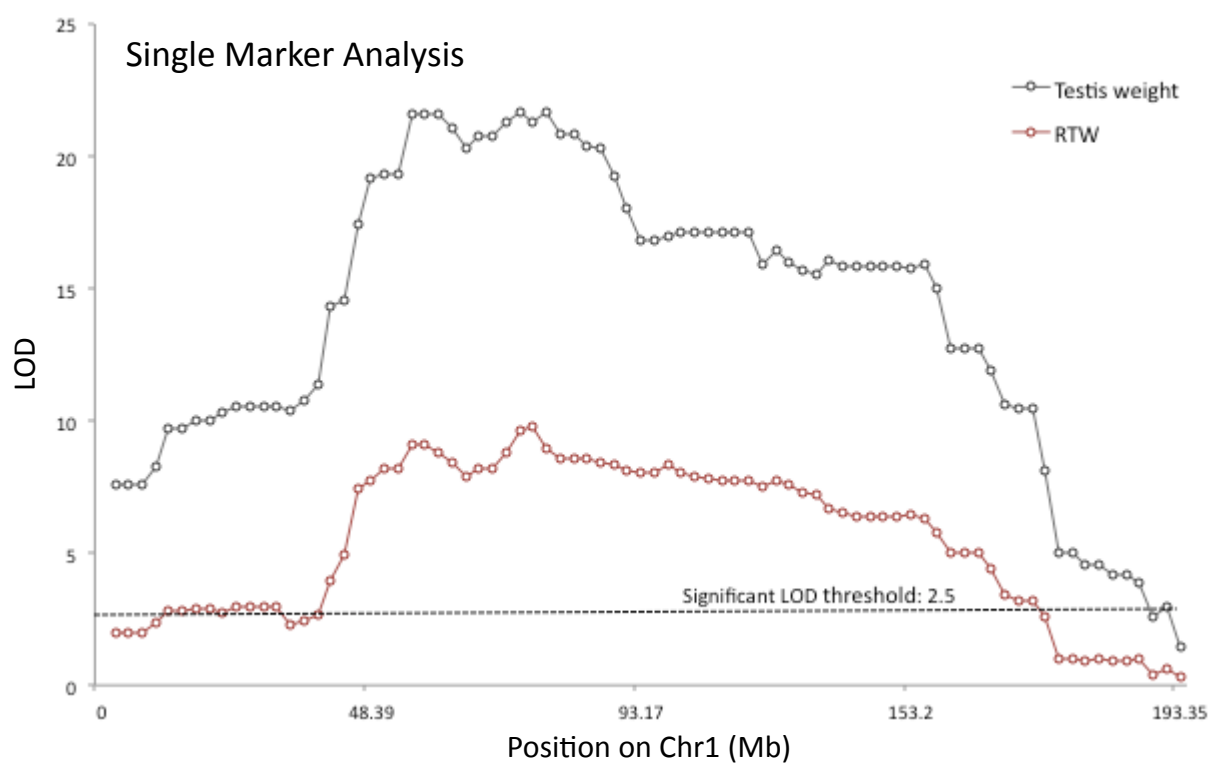

C

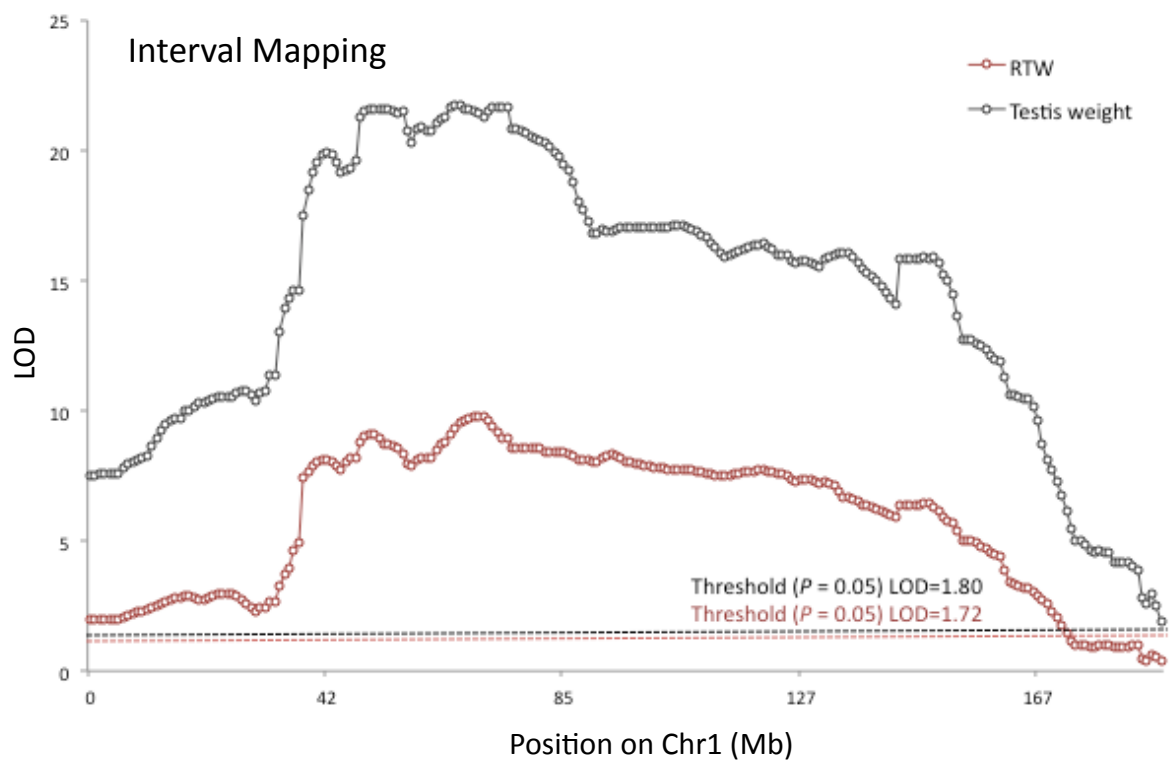

Supplement: Figure S13 — Genetic analysis to detect responsible QTLs on chromosome 1 for the restoration of testis weight. (A) Mating scheme used in the QTL analysis. (B, C) Results of the QTL analysis of chromosome 1. Single marker analysis (B) and interval mapping (C) were performed using 80 SNP markers on chromosome 1 and 314 male progeny. Two traits, testis weight and ratio of testis weight to body weight (RTW), were used. (PDF) [file pgen.1004301.s013.pdf]

Figure S14

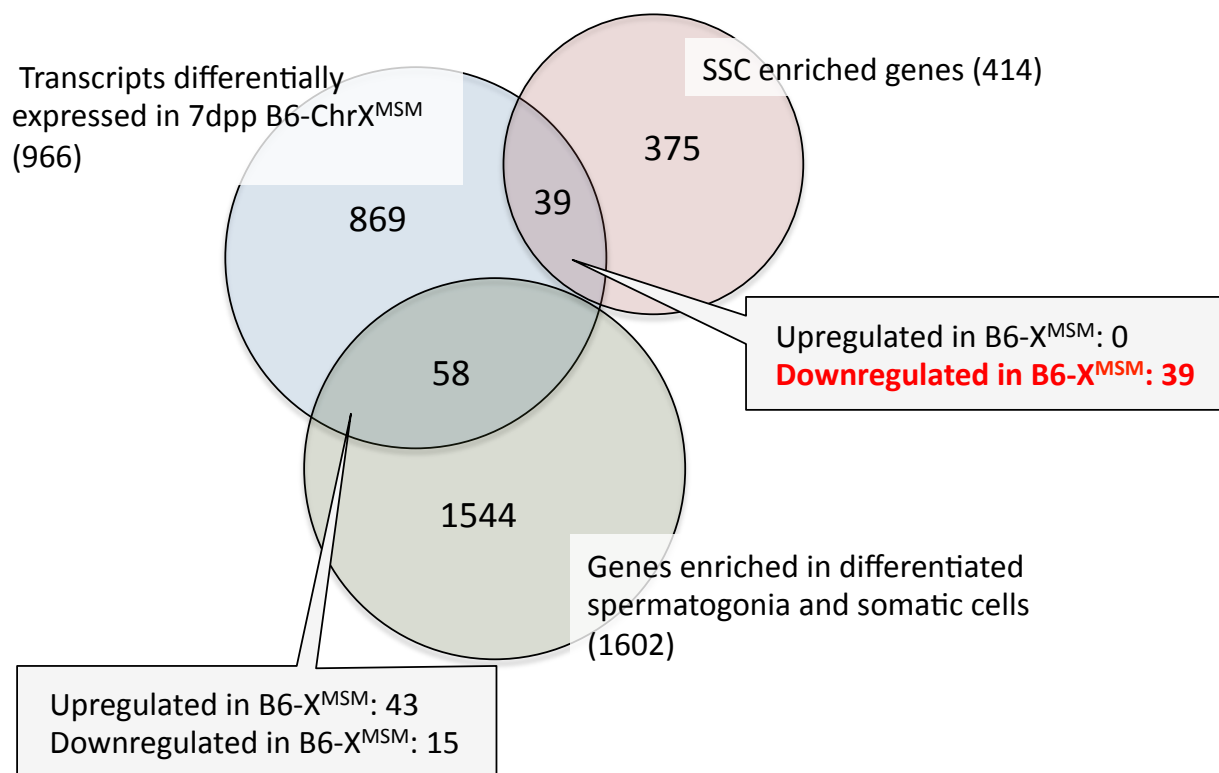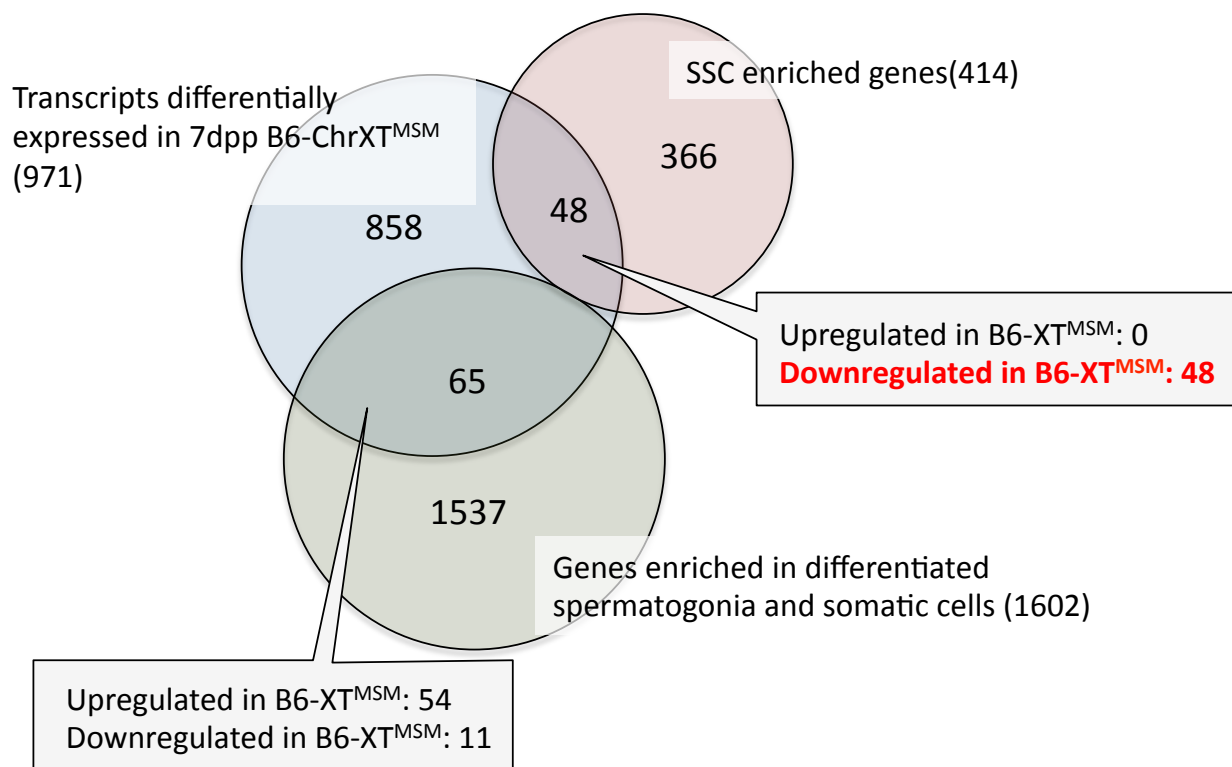

Supplement: Figure S14 — Venn diagram of misregulated SSC-enriched and non-SSC-enriched gene transcripts in B6-ChrXMSM (upper) and B6-ChrXTMSM (lower). SSC-enriched genes show the expression levels higher than 3-fold in non-SSC cells, and non-SSC enriched genes show the expression levels higher than 3-fold in SSCs in a report by Yang et al. Note that all SSC-enriched misregulated genes were downregulated in B6-ChrXMSM and B6-ChrXTMSM. (PDF) [file pgen.1004301.s014.pdf]

Figure S15

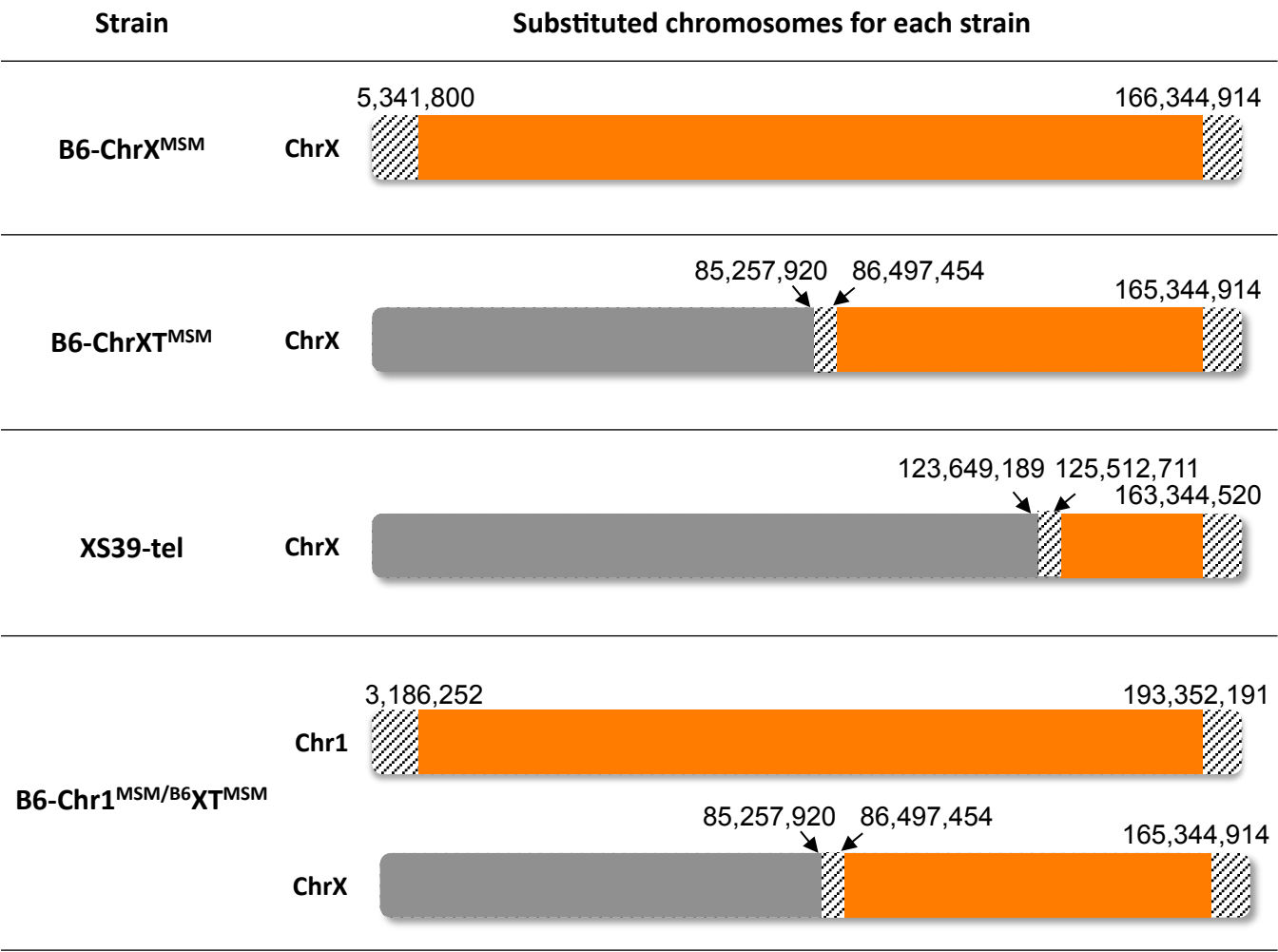

Supplement: Figure S15 — X chromosomal subregion substituted by the MSM-derived counterpart for each strain. The subregions of B6 and MSM genomes are indicated in gray and orange, respectively. Numbers indicate the positions of genetic markers for genotyping at the boundaries. Because the genotypes in stripe subregions are undefined, probe sets in these regions were excluded from the analysis. (PDF) [file pgen.1004301.s015.pdf]
