# Supplementary material for: Evolutionarily Diverged Regulation of X-chromosomal Genes as a Primal Event in Mouse Reproductive Isolation
Source: PLoS Genet. 2014 Apr 17;10(4):e1004301. doi: 10.1371/journal.pgen.1004301 (PMC3990516; doi:10.1371/journal.pgen.1004301)
Supplement: Figure S2 — Criteria for polymorphic probe sets used in the microarray analysis. Polymorphism scores of the Affymetrix Mouse 430 2.0 probe sets are indicated in upper table. Polymorphism scores were defined by the number of probes identified in the MSM genome and the number of polymorphic probes per probe set. In this study, probe sets with polymorphism scores of ≤10 were used in the analysis as conserved probe sets (black square). The numbers of probe sets for each polymorphism score are indicated in lower table. (PDF) [file pgen.1004301.s002.pdf]

Figure S2

Polymorphism scores of the Affymetrix Mouse 430 2.0 probe sets

Number of polymorphic probes per probe set (columns); Number of probes identified in MSM genome per probe set (rows)

	11	10	9	8	7	6	5	4	3	2	1	0
11	67	56	46	37	29	22	16	11	7	4	2	1
10		68	57	47	38	30	23	17	12	8	5	3
9			69	58	48	39	31	24	18	13	9	6
8				70	59	49	40	32	25	19	14	10
7					71	60	50	41	33	26	20	15
6						72	61	51	42	34	27	21
5							73	62	52	43	35	28
4								74	63	53	44	36
3									75	64	54	45
2										76	65	55
1											77	66
0												78

The numbers of probe sets for each polymorphism score

Number of polymorphic probes per probe set (columns); Number of probes identified in MSM genome per probe set (rows)

	11	10	9	8	7	6	5	4	3	2	1	0
11	8	19	30	63	177	387	748	1484	2508	3977	5200	9590
10		1	1	14	24	70	155	358	598	955	1329	2434
9			1	6	9	24	74	167	296	518	834	1649
8				3	4	13	34	65	128	235	387	842
7					3	2	21	23	71	119	163	458
6						1	3	11	24	52	104	290
5							4	5	16	44	53	198
4								9	12	22	56	151
3									7	16	35	142
2										18	29	174
1											38	163
