# Supplementary material for: Evolutionarily Diverged Regulation of X-chromosomal Genes as a Primal Event in Mouse Reproductive Isolation
Source: PLoS Genet. 2014 Apr 17;10(4):e1004301. doi: 10.1371/journal.pgen.1004301 (PMC3990516; doi:10.1371/journal.pgen.1004301)

Figure S11

7 dpp non-restored B6-Chr1$^{MSM/B6}$XT^{MSM}

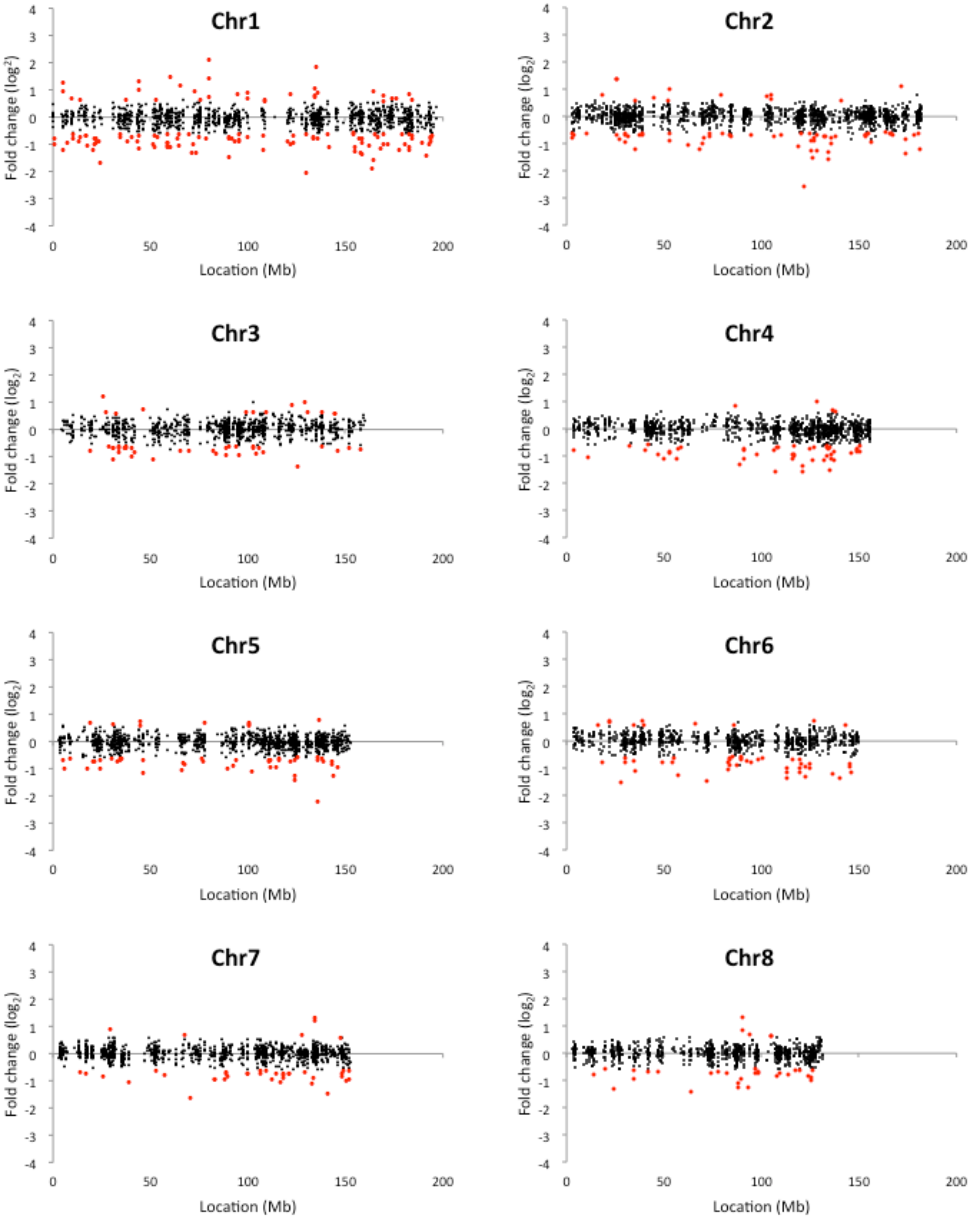

7 dpp non-restored B6-Chr1$^{MSM/B6}$XT^{MSM}

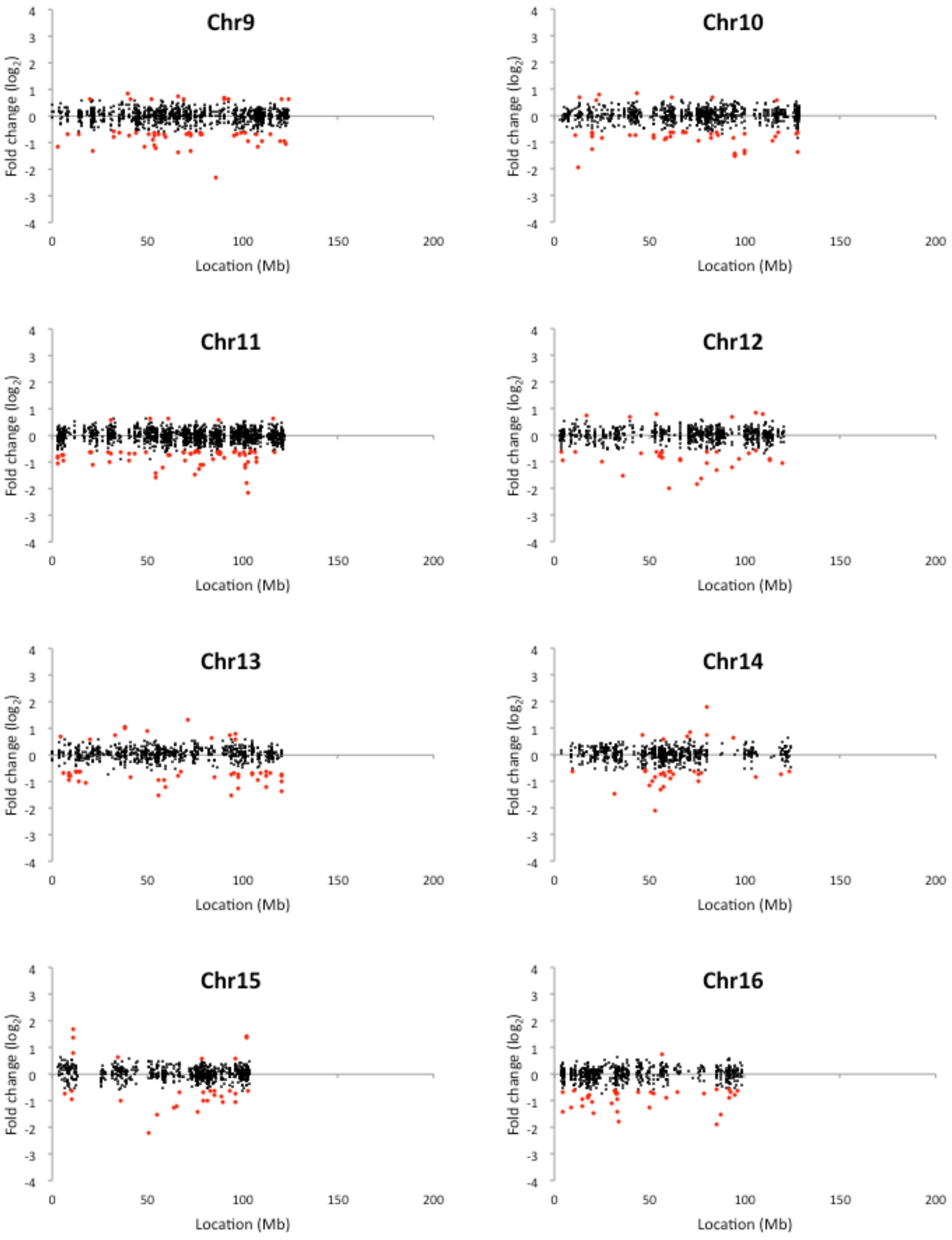

7 dpp non-restored B6-Chr1$^{MSM/B6}$XT^{MSM}

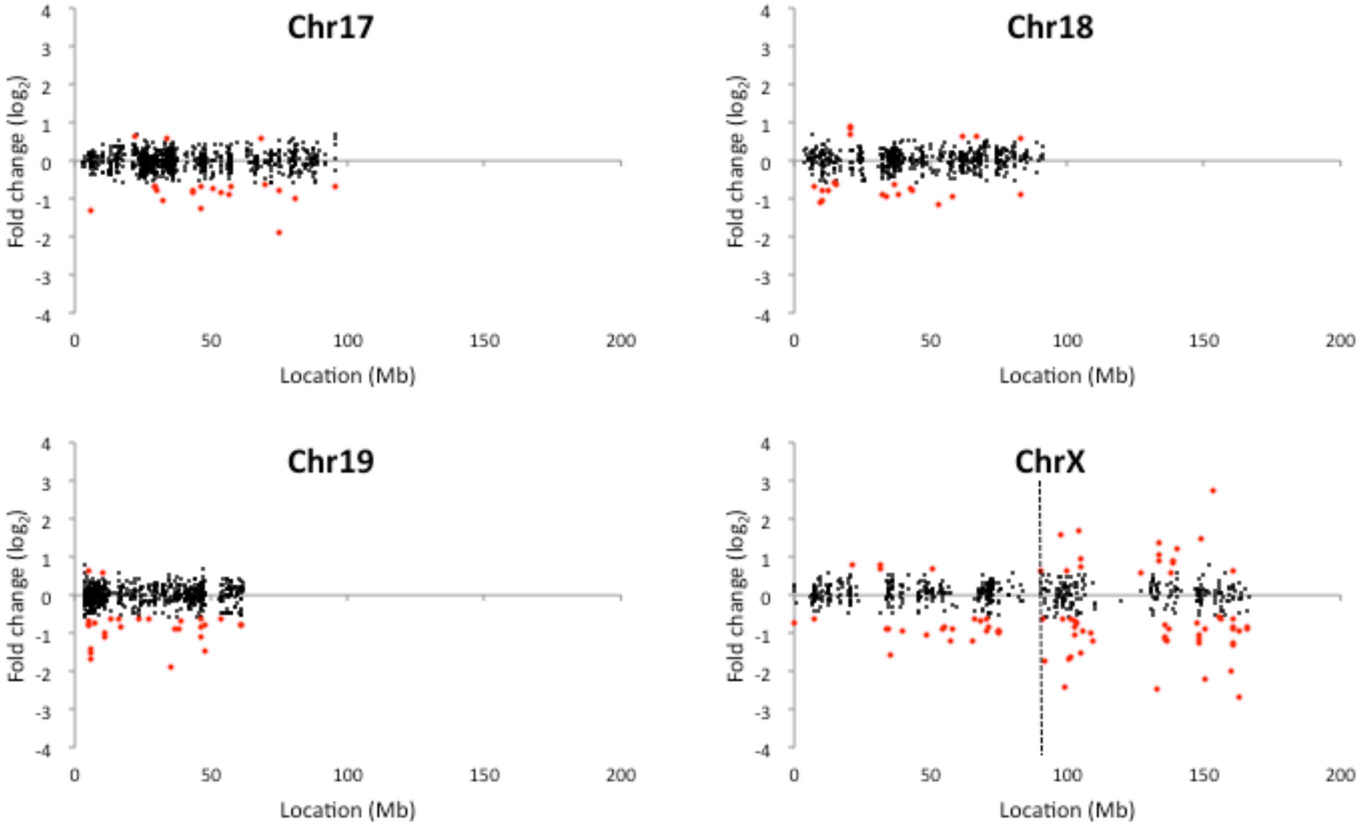

Supplement: Figure S11 — Gene expression in non-restored B6-Chr1MSM/B6XTMSM testes at 7 dpp. Fold changes of gene expression in B6-Chr1MSM/B6XTMSM relative to that in B6 is indicated in a log2 scale. Transcripts in red show significantly different expression by the Benjamini-Hochberg FDR corrected moderate t-test (P<0.05; fold change ≥1.50). (PDF) [file pgen.1004301.s011.pdf]
