# Supplementary material for: Evolutionarily Diverged Regulation of X-chromosomal Genes as a Primal Event in Mouse Reproductive Isolation
Source: PLoS Genet. 2014 Apr 17;10(4):e1004301. doi: 10.1371/journal.pgen.1004301 (PMC3990516; doi:10.1371/journal.pgen.1004301)

Figure S12

Nxf2

F: 5'-GCCTCTGCATTGAAGGATGT-3'
R: 5'-TTTGCACAGAATAGGGCACA-3'
Product size : 100 bp

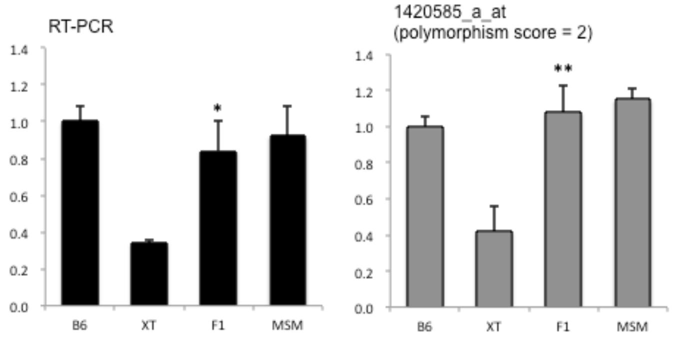

Srpx2

F: 5'-ATGGAATGCTGCTTGACTCC-3'
R: 5'-TCACGAGAGTGAGGACAACG-3'
Product size : 163 bp

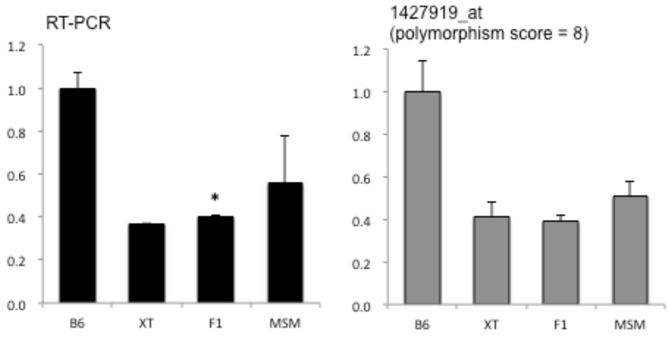

Taf7l

F: 5'-GAAATGCTGCTTGGAAGGAT-3'
R: 5'-TTCCGATAACACAAGGCAGA-3’
product size : 123 bp

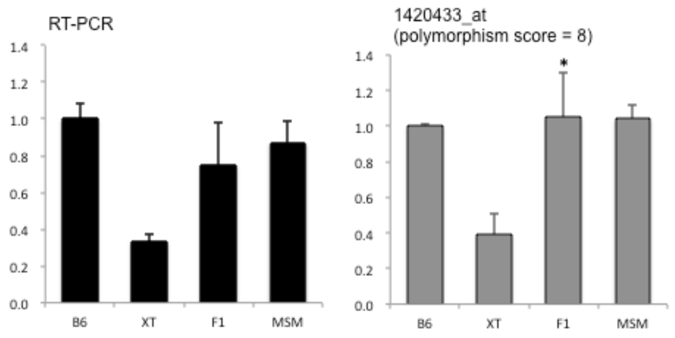

Nxf3

F: 5'-GAAATGGAACCAAACGGATG-3'
R: 5'-ATGCCAAAGGGAATTGTGAC-3'
Product size : 123 bp

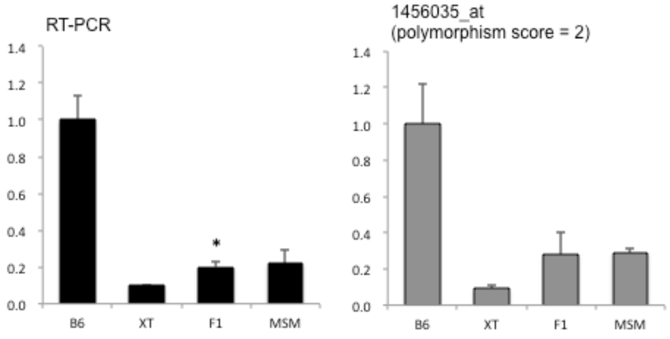

Mum1l1

F: 5'-CCAACAGTCCACCTGACAGA-3'
R: 5'-TTTGATTTGGCACCACAAGA-3'
Product size : 131 bp

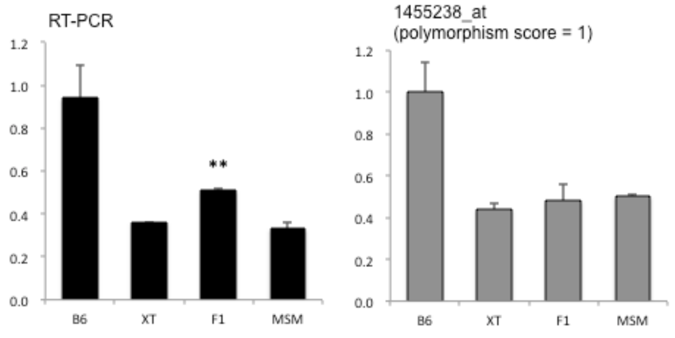

Trap1a

F: 5'-AGAGATGAGCGTGGAAATGG-3'
R: 5'-CAGGAAATTAGGGTCGTGGA-3'
Product size : 139 bp

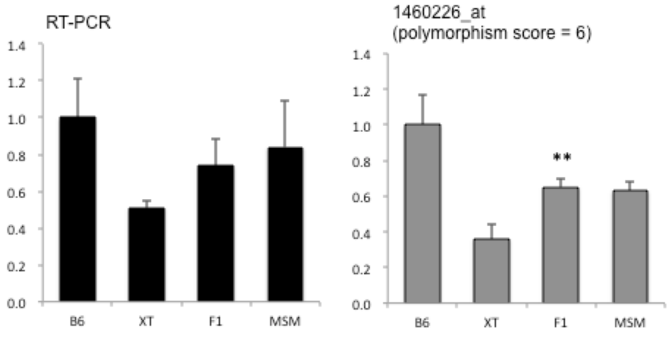

Rnf128

F: 5'-AGAGAGAGGGGCTTCTGGAG-3'
R: 5'-TGACTTGGATGCCTCTTTGA-3'
Product size : 161 bp

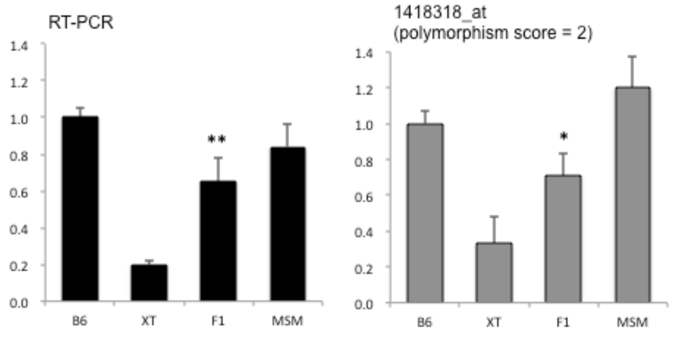

Tsc22d3

F: 5'-GGTGGCCCTAGACAACAAGA-3'
R: 5'-TCTTCTCAAGCAGCTCACGA-3'
Product size : 119 bp

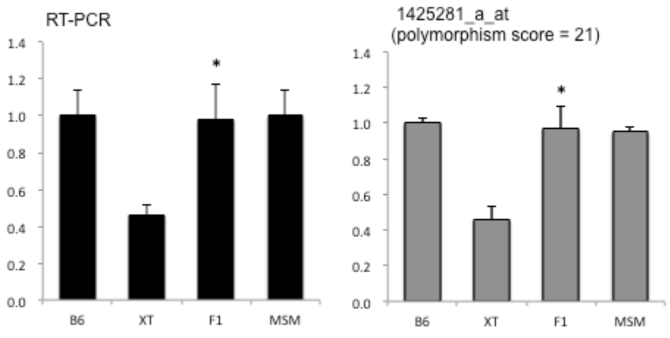

Supplement: Figure S12 — Validation by RT-PCR of eight downregulated X-linked genes. Expression values of B6-ChrXTMSM, B6-Chr1MSM/B6XTMSM, and MSM testes relative to those in B6 controls are shown. Left graphs (black) and right graphs (gray) are results from RT-PCR and microarray experiments, respectively. Values of gene expression in RT-PCR analysis were normalized to β-actin gene expression. XT and F1 indicate B6-ChrXTMSM and B6-Chr1MSM/B6XTMSM, respectively. Significant restoration of values in B6-Chr1MSM/B6XTMSM testes relative to those in B6-ChrXTMSM testes is indicated by single and double asterisks (two-tailed Student's t-test, *P<0.05; **P<0.01). Primers for RT-PCR are indicated above graphs for each gene. Primer for β-actin; F, 5′-ATG ACG ATA TCG CTG CGC TGG T-3′; R, 5′-ATA GGA GTC CTT CTG ACC CAT TCC-3′; Cano DA et al, Development 2004;131(14):3457-67. (PDF) [file pgen.1004301.s012.pdf]
